# Synthesis, Antifungal Activity, and Plant Systemic Mobility of Amino Acid Conjugates of a Thiasporine A-Derived Phenylthiazole Scaffold

**DOI:** 10.3390/molecules31183241

**Published:** 2026-09-14

**Authors:** Li Li, Shunshun Chen, Panpan Yin, Xintao Huang, Jing Li, Ya’xuan Xiao, Changxu Lu, Lin Li, Yang Wang, Xiang Zhu, Linhua Yu, Junkai Li

**Affiliations:** 1 Hubei Key Laboratory of Waterlogging Disaster and Agricultural Use of Wetland, College of Agriculture, Yangtze University, Jingmi Road 88, Jingzhou 434025, China; 2 Institute of Pesticides, Yangtze University, Jingmi Road 88, Jingzhou 434025, China; 3 State Key Laboratory of Green Pesticide, Guizhou University, Guiyang 550025, China

**Keywords:** thiasporine a, amino acid, synthesis, antifungal activities, mobility

## Abstract

To develop fungicidal candidates with both high antifungal activity and phloem mobility, a total of 34 2-[2-(trifluoromethoxy)phenyl]thiazole-4-carbonyl amino acid and amino acid ester conjugates were designed and synthesized. Their structures were characterized by ^1^H NMR, ^13^C NMR, and HRMS. Their in vitro antifungal activities against five phytopathogenic fungi and their phloem and xylem mobility in castor bean seedlings were subsequently evaluated. The results showed that the amino acid conjugates **9a-9p** generally exhibited higher antifungal activity than the corresponding amino acid ester conjugates. Compounds **9m** and **9n** showed good inhibitory activity against *Rhizoctonia solani*, *Sclerotinia sclerotiorum*, *Phoma citricarpa*, and *Phytophthora parasitica*. Notably, compounds **9f**, **9g**, **9l**, and **9n** showed strong inhibitory activity against *P. citricarpa* at 200 μmol/L, with inhibition rates of 82.82%, 89.86%, 80.45%, and 91.55%, respectively, all higher than that of thifluzamide (29.30%). Mobility assays revealed configuration-related differences in the conjugate-derived material detected in phloem and xylem exudates, with differential hydrolysis to compound 3 contributing substantially to the observed L/D profiles In addition, some L-amino acid conjugates were hydrolyzed in castor bean seedlings to release the unconjugated carboxylic acid precursor (compound 3). These results indicate that appropriate amino acid conjugation can enhance antifungal activity and modulate systemic mobility in plants, providing a basis for the structural design of systemic fungicides.

## 1. Introduction

**Figure 1:**
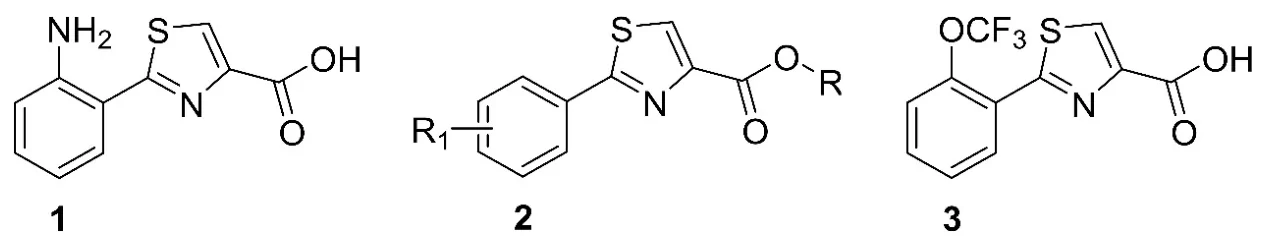
Thiasporine A and its derivatives.

Natural products have long served as important sources of pesticide lead compounds because of their structural diversity, novel biological targets, and unique modes of action. Structural modification of natural products to improve their biological activities and physicochemical properties is an important strategy for the discovery and development of novel pesticides [1,2]. Thiasporine A (Figure 1, **compound 1**), a natural product containing a phenylthiazole scaffold, was isolated in 2015 from metabolites of the marine actinomycete *Actinomycetospora chlora* SNC-032. Previous studies showed that thiasporine A exhibited moderate cytotoxicity against the H2122 lung cancer cell line and has therefore attracted some attention in the pharmaceutical field. However, its potential agricultural applications have rarely been investigated [3,4,5,6,7]. In our previous study, the phenyl ring and carboxyl group of thiasporine A were structurally modified (Figure 1, **compound 2**), and the antifungal activities of the resulting derivatives were evaluated. Several derivatives exhibited promising antifungal activity. In particular, 2-[2-(trifluoromethoxy)phenyl]thiazole-4-carboxylic acid (Figure 1, **compound 3**) showed inhibition rates of more than 70% against *Sclerotium rolfsii* and *Phytophthora parasitica* at 200 μmol/L [8].

Phloem-mobile fungicides can be transported from treated foliage to other parts of the plant, enabling the control of diseases affecting roots or vascular tissues. Accordingly, the development of pesticides with systemic mobility has attracted increasing attention [9]. Current strategies for improving fungicide systemicity mainly include structural modification, optimization of physicochemical properties, and the introduction of endogenous substances capable of directional transport within plants [10,11,12]. Amino acids are important endogenous substances in plants and are transported by specific carrier systems. Therefore, they have been widely used as carrier groups in the design of systemic pesticides. Our previous studies have shown that conjugation with amino acids can improve the systemic mobility of bioactive compounds while maintaining or enhancing their biological activity [13,14,15,16,17].

**Figure 2:**
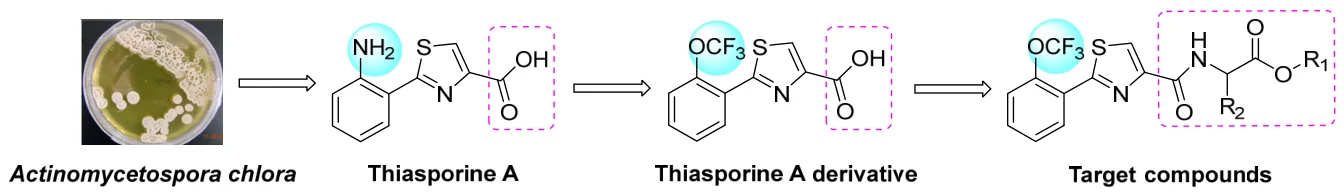
Design strategy of amino acid and amino acid ester conjugates based on the thiasporine A-derived phenylthiazole scaffold.

Based on these findings, 2-[2-(trifluoromethoxy)phenyl]thiazole-4-carboxylic acid was selected as the lead scaffold and coupled with L- or D-amino acid esters through amide bond formation. Subsequent hydrolysis afforded the corresponding amino acid conjugates, yielding a total of 34 previously unreported 2-[2-(trifluoromethoxy)phenyl]thiazole-4-carbonyl amino acid and amino acid ester conjugates. The synthetic strategy is shown in Figure 2. The antifungal activities of the target compounds and their mobility in castor bean (*Ricinus communis* L.) seedlings were subsequently evaluated. The effects of amino acid configuration on antifungal activity, phloem mobility, and xylem mobility were also investigated. This study aimed to identify compounds with favorable in vitro antifungal activity and mobility in a castor bean seedling transport model, and to provide preliminary information for the further design and evaluation of systemic fungicide candidates.

## 2. Results and Discussion

### 2.1. Chemistry

**Figure 3:**
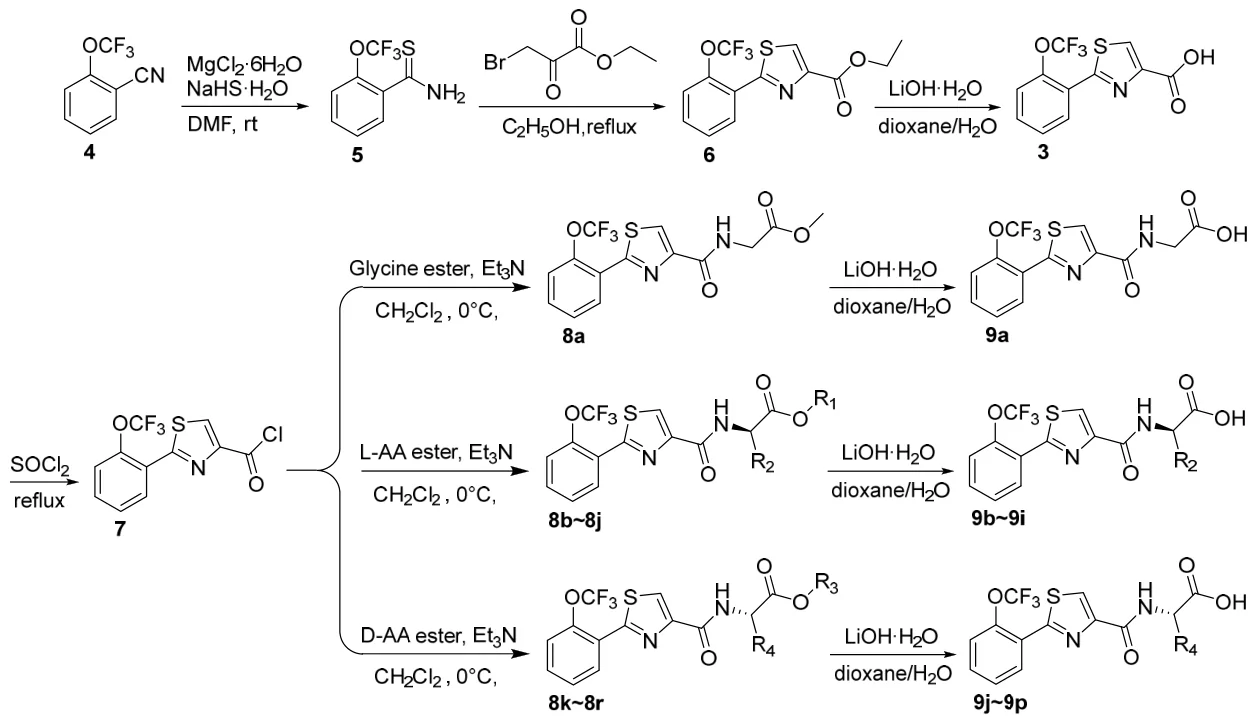
Synthetic routes to compounds **8a-8r** and **9a-9p**.

The synthetic route to compounds **8a-8r** and **9a-9p** is shown in Figure 3. 2-(Trifluoromethoxy)benzonitrile (4) was used as the starting material to prepare 2-[2-(trifluoromethoxy)phenyl]thiazole-4-carboxylic acid (3) through thioamidation, cyclization, and hydrolysis. Compound 3 was then converted to the corresponding acyl chloride with SOCl_2_ and subsequently coupled with amino acid esters to give compounds **8a-8r**. Hydrolysis of compounds **8a-8r** with LiOH afforded compounds **9a-9p**. The first thioamidation reaction was carried out in a sealed vessel to minimize the loss of H_2_S generated from NaHS and thereby ensure sufficient reaction of the starting material. Compounds **8a-8r** were purified by column chromatography, whereas compounds **9a-9p** were purified by recrystallization from ethyl acetate.

All target compounds were characterized by ^1^H NMR, ^13^C NMR, and HRMS. Compound **8b** was selected as a representative example. In its ^1^H NMR spectrum, the thiazole proton appeared at *δ* 8.88, while the amide NH proton was observed at *δ* 8.61. The aromatic protons appeared in the range of *δ* 8.50–7.55. The methine proton adjacent to the ester group was observed at *δ* 4.60, while the methoxy protons gave a singlet at *δ* 3.67 and the methyl protons appeared at *δ* 1.47. In the ^13^C NMR spectrum of compound **8b**, the ester carbonyl carbon appeared at *δ* 173.22, while the three thiazole carbons were observed at *δ* 160.55, 145.78, and 128.38. The amide carbonyl carbon resonated at *δ* 160.41. The six aromatic carbons of the phenyl ring appeared at *δ* 149.48, 132.65, 130.55, 126.83, 125.61, and 121.45. The carbon of the trifluoromethoxy group was observed at *δ* 120.33, while the methoxy carbon appeared at *δ* 52.49. The methine carbon adjacent to the ester group resonated at *δ* 48.17, and the methyl carbon appeared at *δ* 17.39. In the HRMS spectrum of compound **8b**, a protonated molecular ion peak [M + H]^+^ was observed at m/z 375.0621, consistent with the calculated value and further confirming the structure of compound **8b** (Table 1).

### 2.2. Antifungal Activity

The in vitro antifungal activities of compounds **8a–8r** and **9a–9p** against *Rhizoctonia solani*, *Sclerotium rolfsii*, *Phoma citricarpa*, *Sclerotinia sclerotiorum*, and *P. parasitica* were evaluated at 200 μmol/L using the mycelial growth rate method, with thifluzamide as the positive control. The results are presented in Table 2.

As shown in Table 2, most of the target compounds exhibited antifungal activity against the five tested phytopathogenic fungi. Overall, the amino acid ester conjugates **8a-8r** showed relatively weak activity. Among them, compounds **8c**, **8k**, and **8l** inhibited *P. citricarpa* by 51.96%, 53.07%, and 67.06%, respectively. Compound **8a** showed relatively broad-spectrum activity, with inhibition rates of 57.33%, 67.28%, 69.54%, and 50.34% against *R. solani*, *S. rolfsii*, *P. citricarpa*, and *S. sclerotiorum*, respectively. In contrast, the amino acid conjugates **9a-9p** generally exhibited stronger antifungal activity. Among the amino acid conjugates, **9m** and **9n** inhibited *P. parasitica* by more than 65%, whereas **9d**, **9f**, **9g**, **9m**, and **9n** showed approximately 70% or greater inhibition against *R. solani*. Compound **9a** was particularly active against *S. rolfsii* (75.0% inhibition), while compounds **9d**, **9f**, **9g**, **9m**, and **9n** exhibited inhibition rates exceeding 60% against *S. sclerotiorum*.

The strongest activity was observed against *P. citricarpa*. Compounds **9d**, **9f**, **9g**, **9l**, **9m**, and **9n** showed inhibition rates of 79.08%, 82.82%, 89.86%, 80.45%, 76.31%, and 91.55%, respectively, with compound **9n** exhibiting the highest activity. Compared with thiasporine A, most amino acid conjugates showed improved antifungal activity. In addition, several compounds were more active than thifluzamide against *P. citricarpa* and *P. parasitica*. The strongest activity was observed against *P. citricarpa*. Compounds **9d**, **9f**, **9g**, **9l**, **9m**, and **9n** showed inhibition rates of 79.08%, 82.82%, 89.86%, 80.45%, 76.31%, and 91.55%, respectively, with compound **9n** exhibiting the highest activity. Compared with thiasporine A, most amino acid conjugates showed improved antifungal activity. In addition, several compounds were more active than thifluzamide against *P. citricarpa* and *P. parasitica*. Overall, no consistent difference in antifungal activity was observed between the corresponding L- and D-amino acid derivatives, suggesting that stereochemical configuration had only a limited effect within the present dataset. However, this observation was not confirmed by paired statistical analysis. From a preliminary SAR perspective, the amino acid conjugates generally showed higher activity than their corresponding ester derivatives, indicating that the free carboxyl group may be beneficial for antifungal activity. Nevertheless, the improved activity cannot be attributed solely to the free carboxyl group, because conversion from the ester derivatives to the corresponding carboxylic acid conjugates may also affect ionization state, lipophilicity, aqueous solubility, membrane permeability, and metabolic stability. Therefore, the antifungal activity of this series is likely influenced by the combined effects of the acid/ester form, amino acid side chain, and overall physicochemical profile.

Based on the preliminary screening, compounds with inhibition rates above 60% against all three pathogens, *R. solani*, *P. citricarpa*, and *S. sclerotiorum*, at 200 μmol/L were selected for EC_50_ determination (Table 3). This criterion was applied to focus the dose–response evaluation on compounds with relatively broad and strong activity. Compound **9g** showed an EC_50_ value of 42.75 μmol/L against *R. solani*, and compound **9n** showed an EC_50_ value of 35.62 μmol/L against *S. sclerotiorum*. Against *P. citricarpa*, compounds **9m** and **9n** showed EC_50_ values of 59.69 and 54.97 μmol/L, respectively, both lower than that of thifluzamide (443.14 μmol/L).

### 2.3. Phloem and Xylem Mobility in Castor Bean Seedlings

#### 2.3.1. Phloem Mobility

The concentrations of thiasporine A and compounds **9a-9p** in the phloem exudates of castor bean seedlings were determined using the method described previously, and the results are presented in Table 4. When the compounds were supplied at 200 μmol/L, most of the amino acid conjugates were detected in the phloem exudates, whereas the tryptophan conjugates were not detected, indicating that most of these conjugates were capable of phloem translocation. In addition, compound 3 was detected following treatment with the glycine conjugate and several L-amino-acid-derived conjugates **(9a-9h)**, which is consistent with hydrolysis of these conjugates in planta. However, the present experiments do not establish where or when the cleavage occurred, and the enzymatic mechanism responsible for the hydrolysis remains to be determined. Compounds **9a**, **9c**, **9d**, **9j**, and **9l** showed relatively high phloem mobility in castor bean seedlings, with total concentrations of 54.66, 40.33, 41.16, 39.10, and 36.12 μmol/L, respectively. After treatment with compounds **9b** and **9e**, the highest levels of total conjugate-derived material were detected in the phloem exudates, reaching 79.54 and 88.46 μmol/L, respectively. However, most of the recovered material corresponded to compound 3 rather than the intact conjugates. Thiasporine A also showed high phloem mobility, with a concentration of 124.02 μmol/L.

#### 2.3.2. Kleier Model Prediction of Phloem Mobility

**Figure 4:**
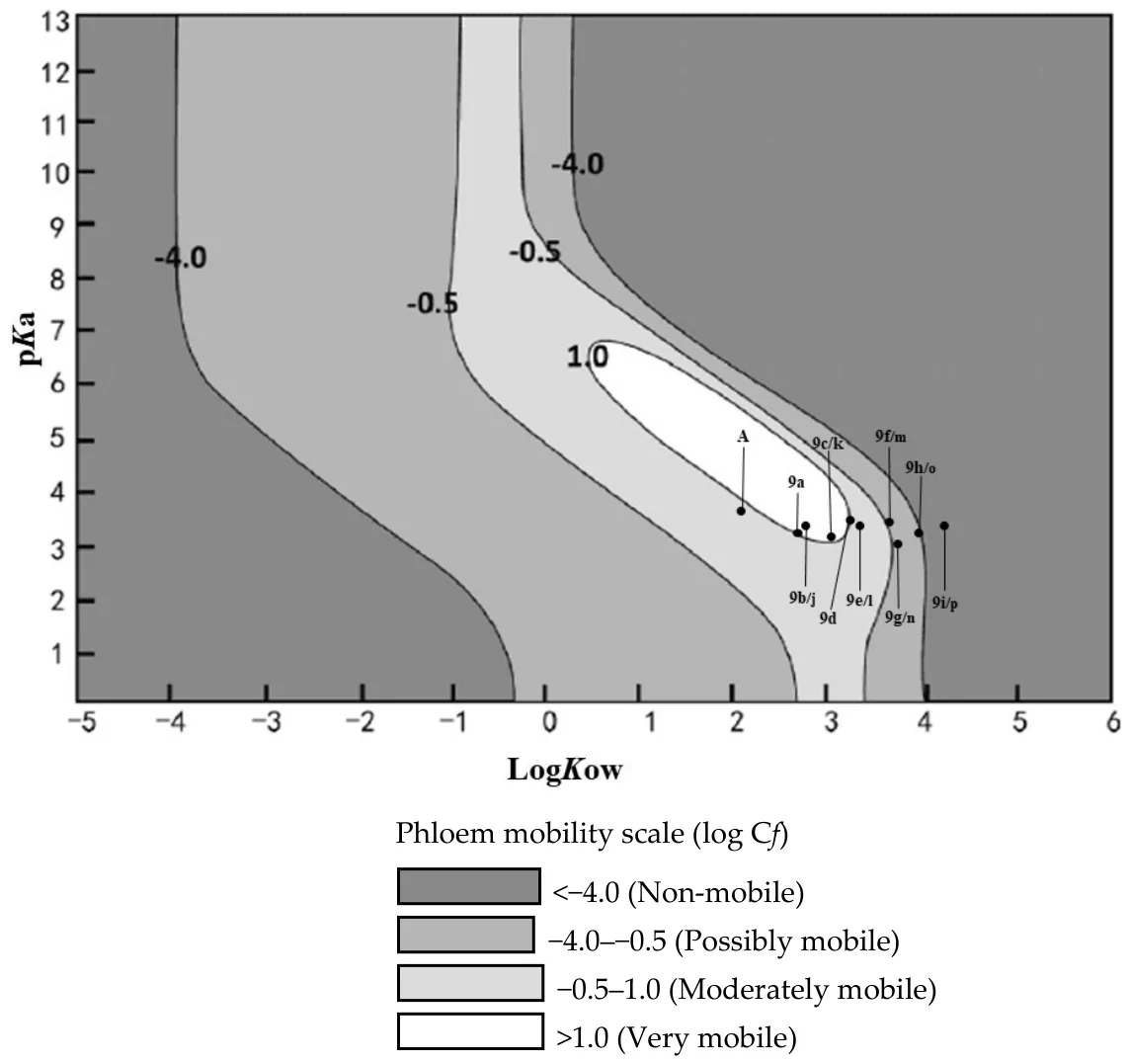
Prediction of the phloem mobility of the tested compounds using the Kleier model.

The phloem transport of exogenous compounds in plants mainly involves two mechanisms: passive diffusion and carrier-mediated transport. Compounds with suitable physicochemical properties, particularly appropriate octanol/water partition coefficients (LogKow) and acid dissociation constants (pKa), can enter plant cells through passive diffusion. Kleier et al. established a predictive model for phloem mobility based on the LogKow and pKa values of exogenous compounds, which has been widely applied in studies of phloem translocation [18,19,20]. Besides passive diffusion, certain exogenous compounds may also be recognized by specific transporters in plants and translocated through carrier-mediated transport [21,22,23,24]. To further investigate the possible mechanism underlying phloem transport, the LogKow and pKa values of the target compounds were calculated using ALOGPS 2.1 and ACD Log D v 6.00, respectively, and are summarized in Table 5. These parameters were then used in the Kleier model to predict phloem mobility, as shown in Figure 4, where A denotes thiasporine A.

According to the Kleier model, compounds **9i** and **9p** were classified as non-mobile, compounds **9f**, **9g**, **9h**, **9m**, **9n**, and **9o** as possibly mobile, compounds **9d** and **9l** as moderately mobile, and compound **9b** as very mobile. The predictions were partly consistent with the experimental results in castor bean seedlings, but several discrepancies were observed. For example, thiasporine A and compound **9e** were predicted to be moderately mobile but showed relatively high phloem concentrations, whereas compounds **9a**, **9c**, **9j**, and **9k** were predicted to be very mobile but showed lower experimental concentrations. In addition, corresponding L- and D-isomers had identical calculated LogKow and pKa values and were assigned to the same mobility category, but some of them showed different measured phloem concentrations. These results suggest that LogKow- and pKa-based passive diffusion alone may not fully explain the phloem mobility behavior of these compounds. However, the discrepancies should not be interpreted as direct evidence for transporter-mediated uptake, because other factors, such as chemical stability, hydrolysis, membrane permeability, ionization state, binding or sequestration, and metabolism, may also affect the detected concentrations in phloem exudates. Therefore, the possible involvement of endogenous amino acid transport systems remains hypothetical and requires further experimental validation.

#### 2.3.3. Xylem Mobility

Xylem translocation of thiasporine A and compounds **9a-9p** was evaluated by analyzing their concentrations in the xylem exudates of castor bean seedlings (Table 6). After treatment with 200 μmol/L of each compound, compound-derived material was detected in the xylem exudates for most treatments, whereas several compounds, including the tryptophan conjugates, were not detected. Compound 3 was detected after treatment with the glycine conjugate and several L-amino-acid-derived conjugates, suggesting that these compounds were hydrolyzed to compound 3 in planta. A broadly similar recovery pattern was observed in the phloem and xylem exudates. However, the current experiments cannot determine whether hydrolysis to compound 3 occurred before, during, or after vascular translocation, nor can they identify the enzymes responsible. After treatment with compounds **9a**, **9b**, **9c**, **9d**, **9e**, **9f**, **9j**, and **9l**, the total concentrations of intact conjugate plus compound 3 detected in the xylem exudates were higher than that of thiasporine A. Among the tested compounds, **9f** was detected as the major intact conjugate in xylem sap, with an intact conjugate concentration of 19.15 μmol/L and a total concentration of 30.34 μmol/L.

### 2.4. Preliminary Analysis of Structure–Activity and Mobility Relationships

**Figure 5:**
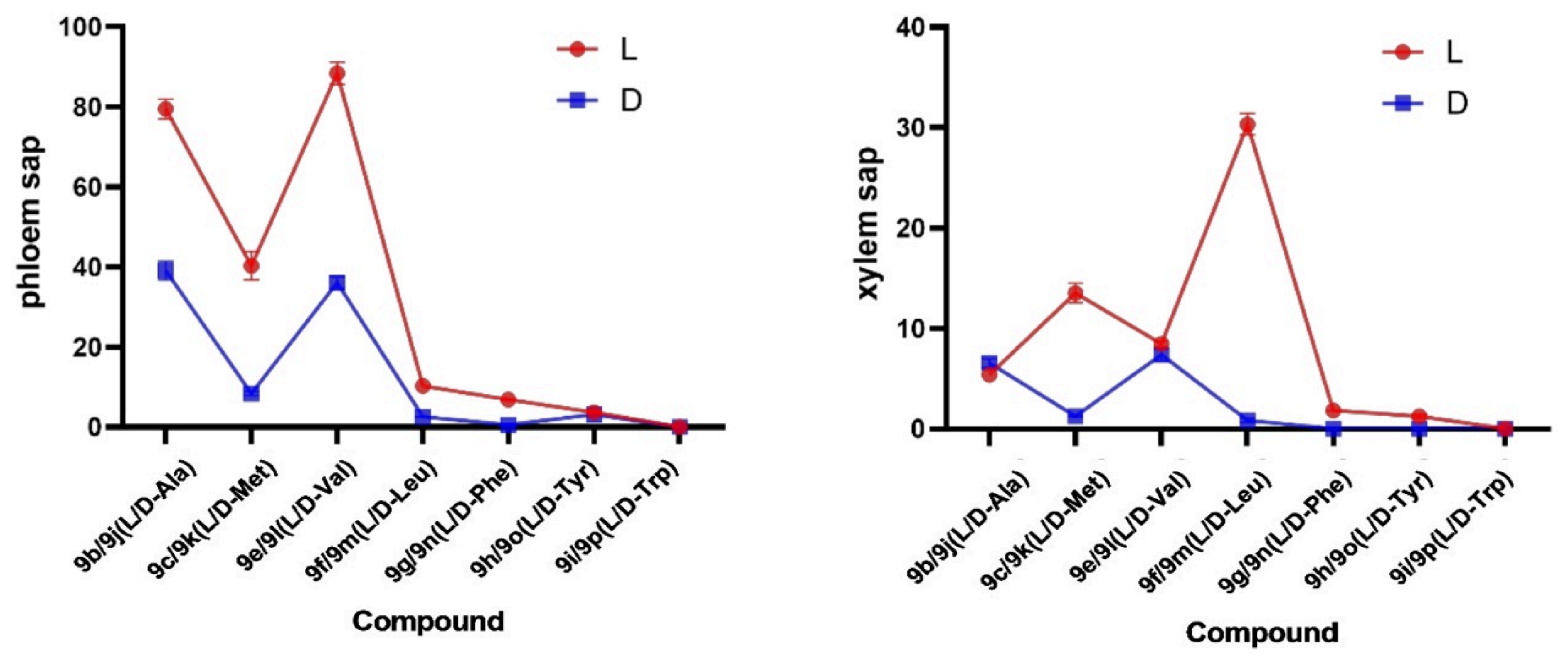
Sum of intact conjugate and compound 3 concentrations in phloem and xylem sap after treatment with selected L- and D-amino acid conjugates.

The in vitro antifungal assays indicated that stereochemical configuration had only a limited effect on antifungal activity within the present dataset. The concentrations obtained after treatment with the corresponding L- and D-amino acid conjugates were further compared in phloem and xylem exudates of castor bean seedlings. As summarized in Figure 5, several L-amino acid conjugate treatments showed higher summed concentrations of intact conjugate and compound 3 than their corresponding D-amino acid conjugate treatments. However, the values in Figure 5 represent the sum of the intact conjugate and compound 3 detected in the sap, rather than the concentration of the intact conjugate alone. Therefore, these L/D differences should be interpreted as configuration-related trends in total detected material, not as direct evidence for greater transport of intact L-conjugates. In addition, hydrolysis to compound 3, particularly for the glycine conjugate and several L-amino-acid-derived conjugates, may contribute substantially to the measured sap concentrations. The present experiments cannot determine whether hydrolysis to compound 3 occurred before, during, or after vascular translocation.

From a preliminary structure–activity and mobility relationship perspective, the activity–mobility profile of this series appears to be associated with multiple factors, including amino acid side chain, acid/ester form, lipophilicity, stereochemical configuration, and hydrolysis behavior. The amino acid conjugates generally showed stronger antifungal activity than their corresponding ester derivatives, suggesting that the free carboxyl group may be beneficial for activity. However, this effect cannot be attributed solely to the free carboxyl group, because conversion from ester derivatives to the corresponding carboxylic acid conjugates may also affect ionization state, lipophilicity, aqueous solubility, membrane permeability, and metabolic stability. Derivatives bearing branched aliphatic or aromatic side chains, such as Ile, Leu, Val, and Phe conjugates, tended to show stronger activity against several tested fungi, especially *P. citricarpa*. Nevertheless, higher lipophilicity did not necessarily lead to better mobility, as the Trp conjugates had relatively high calculated LogKow values but were not detected in phloem or xylem sap. Overall, the antifungal activity and plant mobility of this series appear to be influenced by a combination of structural and physicochemical factors rather than by a single parameter. The possible contribution of endogenous amino acid transport systems remains hypothetical and requires further experimental validation.

## 3. Materials and Methods

### 3.1. Materials and Instruments

Thifluzamide (98% purity) was provided by the Institute of Pesticides, Yangtze University. 2-(Trifluoromethoxy)benzonitrile, ethyl tribromopyruvate, L- and D-amino acid ester derivatives, and other reagents were commercially available and used without further purification. The commercially obtained L- and D-amino acid ester derivatives had chemical purities of ≥98% and were used as stereochemically defined starting materials. In the coupling reaction, the amino acid carboxyl group remained ester-protected, while the carboxyl group of the thiazole scaffold was activated as the corresponding acid chloride.

The main instruments used in this study were a WRR melting point apparatus (Shanghai Jingke Industrial Co., Ltd., Shanghai, China), a Bruker Avance DPX400 NMR spectrometer (Bruker, Rheinstetten, Germany) with TMS as the internal standard and DMSO-*d*_6_ as the solvent, a Q Exactive high-resolution mass spectrometer with an ESI source (Thermo Scientific, Bremen, Germany), an SW-CJ-2FD clean bench (Suzhou Antai Airtech Co., Ltd., Suzhou, China), a BXM-30R/YXQ autoclave and a BIC-400 light incubator (Shanghai Boxun Medical Biological Instrument Corp., Shanghai, China), an LC7000 high performance liquid chromatograph (Shandong Wooking Instrument Co., Ltd., Qingdao, China), and a TU-1901 double beam UV visible spectrophotometer (Beijing Purkinje General Instrument Co., Ltd., Beijing, China).

### 3.2. Bacterial and Fungal Strains

The plant pathogens *R. solani*, *S. rolfsii*, *P. citricarpa*, *S. sclerotiorum*, and *P. parasitica* were provided by the School of Agriculture, Yangtze University. Seeds of castor bean were obtained from the Zibo Academy of Agricultural Sciences, Shandong, China.

### 3.3. Chemical Synthesis

#### 3.3.1. General Procedure for the Preparation of Intermediate 5

Intermediate 5 was prepared according to previously reported methods [4,17]. 2-(trifluoromethoxy)benzonitrile (10 mmol), DMF (30 mL), and MgCl_2_·6H_2_O (10 mmol) were placed in a 100 mL single-neck flask at room temperature and stirred until a homogeneous solution was obtained. NaHS·H_2_O (20 mmol) was then added, and the mixture was stirred for approximately 16 h. The reaction was monitored by TLC using petroleum ether and ethyl acetate (3:1, *v*/*v*) as the eluent. Upon completion, the reaction mixture was diluted with water (70 mL) and saturated brine (10 mL), followed by extraction with ethyl acetate (4 × 50 mL). The combined organic phases were dried over anhydrous Na_2_SO_4_ and filtered. Removal of the solvent under reduced pressure afforded intermediate 5, which was used directly in the subsequent reaction without further purification.

#### 3.3.2. General Procedure for the Preparation of Intermediate 6

Intermediate 5 (10 mmol) was dissolved in anhydrous ethanol (30 mL) in a 100 mL single-necked flask, and ethyl 3-bromopyruvate (10 mmol) was added. The reaction mixture was refluxed at 65 °C for approximately 2 h and monitored by TLC using petroleum ether/ethyl acetate (3:1, *v*/*v*) as the eluent. After completion, saturated NaHCO_3_ solution was added dropwise until the mixture became slightly basic. The solvent was removed under reduced pressure, and the residue was taken up in dichloromethane (50 mL) and washed with saturated brine (2 × 50 mL). The organic layer was dried over anhydrous Na_2_SO_4_, filtered, and concentrated. Purification of the crude product by silica gel column chromatography using petroleum ether/ethyl acetate (6:1, *v*/*v*) afforded intermediate 6.

#### 3.3.3. General Procedure for the Preparation of Intermediate 3

Compound 6 (10 mmol) was dissolved in 1,4-dioxane (30 mL), and an aqueous solution of LiOH·H_2_O (10 mmol in 30 mL water) was added slowly. The reaction mixture was refluxed at 65 °C for approximately 2 h and monitored by TLC using petroleum ether/ethyl acetate (1:1, *v*/*v*). After the reaction was complete, the mixture was acidified to pH 2–3 with 1 M HCl to a slightly acidic pH and stirred at room temperature to induce precipitation. The resulting solid was collected by vacuum filtration and dried to give compound 3 as a white solid.

#### 3.3.4. General Procedure for the Preparation of Target Compounds **8a-8r**

2-[2-(Trifluoromethoxy)phenyl]thiazole-4-carboxylic acid (3, 10 mmol) was treated with SOCl_2_ (15 mL) in dichloromethane (30 mL) in the presence of a catalytic amount of DMF (2 drops). The mixture was refluxed at 40 °C for 2 h, and the reaction was monitored by TLC using petroleum ether/ethyl acetate (3:1, *v*/*v*). After completion, the volatile components, including excess SOCl_2_, were removed under reduced pressure. The resulting acid chloride 7 was redissolved in dichloromethane (30 mL) and used directly in the subsequent coupling reaction. The corresponding amino acid ester hydrochloride (10 mmol) and triethylamine (20 mmol) were stirred in CH_2_Cl_2_ (30 mL) under ice-bath cooling for 2 h. A CH_2_Cl_2_ solution of acid chloride 7 was then added dropwise to the reaction mixture under ice-bath cooling. The reaction was monitored by TLC using petroleum ether and ethyl acetate (3:1, *v*/*v*) as the eluent. Upon completion, the mixture was diluted with CH_2_Cl_2_ (50 mL) and washed with saturated brine (2 × 100 mL). The organic layer was dried over anhydrous Na_2_SO_4_, filtered, and concentrated under reduced pressure. Purification of the resulting residue by silica gel column chromatography using petroleum ether and ethyl acetate (6:1, *v*/*v*), followed by drying, afforded the target compounds **8a-8r**.

#### 3.3.5. General Procedure for the Preparation of Target Compounds **9a-9p**

Compounds **8a-8r** (10 mmol) were dissolved in 1,4-dioxane (30 mL) in a 100 mL single-necked flask. LiOH·H_2_O (10 mmol) was separately dissolved in water (30 mL), and the resulting aqueous solution was slowly added to the reaction mixture. The mixture was heated to 65 °C and refluxed for approximately 2 h. The reaction was monitored by TLC using petroleum ether/ethyl acetate (1:1, *v*/*v*) as the eluent. Upon completion, 1 M HCl was added dropwise until the mixture became slightly acidic, and the mixture was then stirred at room temperature to induce precipitation. The resulting solid was collected by vacuum filtration and dried to afford the target compounds **9a-9p**. The structures of the target compounds were confirmed by ^1^H NMR, ^13^C NMR, and HRMS. The spectroscopic data for compounds **8a-8r** and **9a-9p** are provided in the Supplementary Materials. The L/D designations of the final conjugates were assigned according to the configurations of the corresponding commercially obtained L- or D-amino acid ester starting materials. However, the enantiomeric purity of the final conjugates was not directly determined by chiral HPLC or other chiral analytical methods. Although no intentional racemization step was introduced, partial epimerization during the coupling and/or hydrolysis steps cannot be completely excluded.

### 3.4. In Vitro Antifungal Activity

The in vitro antifungal activities of compounds **8a-8r** and **9a-9p** against five phytopathogenic fungi were determined by the mycelial growth rate method [17]. Each compound was dissolved in DMSO and subsequently diluted with sterile water containing 0.1% Tween 80 to prepare a 2000 μmol/L stock solution. The stock solution (5 mL) was thoroughly mixed with 45 mL of PDA medium to give a final test concentration of 200 μmol/L.

A 7 mm mycelial disk was excised from the actively growing edge of each fungal colony and placed in the center of the PDA plate. The inoculated plates were incubated at 28 °C. The corresponding solvent treatment served as the negative control, whereas thifluzamide (200 μmol/L) was used as the positive control. Each treatment consisted of three separate replicate plates within the same experimental run. For each plate, colony diameters were measured along two perpendicular axes, and the mean of the two measurements was used as the colony diameter for that plate. The results are presented as the mean ± SD of the three replicate plates. The inhibition of mycelial growth was calculated using Equation (1):

Inhibition rate (%) = [(CK − T)/(CK − 7)] × 100(1)

where CK is the mean colony diameter of the untreated control, T is the mean colony diameter after treatment, and 7 mm is the diameter of the inoculated mycelial plug.

Compounds showing more than 60% inhibition against *R. solani*, *P. citricarpa*, and *S. sclerotiorum* at 200 μmol/L were further evaluated for EC_50_ values. For the selected target compounds, EC_50_ values were determined using final concentrations of 12.5, 25, 50, 100, and 200 μmol/L. For thifluzamide, pathogen-specific concentration ranges were selected based on preliminary antifungal assays to ensure that the 50% inhibition response was bracketed by the tested concentrations. Lower concentration ranges were used for *R. solani* and *S. sclerotiorum*, whereas a higher concentration range was used for *P. citricarpa*. EC_50_ values were determined by regression analysis of the probit-transformed inhibition rates against the logarithm of compound concentration (DPS).

### 3.5. Determination of Phloem and Xylem Mobility in Castor Bean Seedlings

#### 3.5.1. Determination of Phloem Mobility

For both phloem and xylem mobility experiments, each treatment consisted of three independent biological replicates. Each replicate contained 12 castor bean seedlings, and the exudates collected from the 12 seedlings were pooled to obtain one biological replicate sample for HPLC analysis. Thus, a total of 36 seedlings were used for each treatment (*n* = 3 biological replicates). Following the method reported in Ref. [17], test solutions were prepared in phosphate buffer at pH 5.8, with each compound at a final concentration of 200 μmol/L. Each compound was first dissolved in 0.5 mL DMSO and then diluted to 100 mL with 0.2 mol/L NaH_2_PO_2_ and Na_2_HPO_4_ buffer.

Uniformly germinated castor bean seeds were transferred to the potting substrate after approximately 2 d of germination and grown for an additional 6 d. Seedlings of similar size and developmental stage were selected, gently washed, and the endosperm was carefully removed. For phloem uptake experiments, the cotyledons were exposed to the test solution, while the roots were immersed in 0.5 mmol/L CaCl_2_. After 2 h, the seedlings were cut at the hypocotyl hook below the cotyledons. Phloem exudates produced between 2 and 4 h after treatment were collected with capillary tubes for subsequent analysis.

#### 3.5.2. Determination of Xylem Mobility

To evaluate xylem mobility, castor bean seedlings were exposed to 200 μmol/L solutions of the test compounds through the roots. After 1 h, the shoots were excised approximately 1 cm above the root system. The cut surface was rinsed with 1 mmol/L CaCl_2_ solution, and the initial exudate was removed after 1 min. Xylem exudates produced during the following 1–4 h were collected with capillary tubes for subsequent analysis.

#### 3.5.3. Analysis of Phloem and Xylem Exudates from Castor Bean Seedlings

The collected phloem and xylem exudates were appropriately diluted with purified water and filtered through a 0.22 μm membrane filter before HPLC analysis. Separation was performed on a C18 reversed-phase column (4.6 mm × 150 mm, 5 μm) maintained at 35 °C, with methanol and 0.1% aqueous formic acid as the mobile phases at a flow rate of 1.0 mL/min. The injection volume was 10 μL. UV detection was carried out at 356 nm for thiasporine A and at 288 nm for the other test compounds. A gradient elution program was used, and the detailed conditions are listed in Table 7.

Standard solutions of the test compounds ranging from 0.5 to 200 μmol/L were analyzed under the HPLC conditions described above. Calibration curves were established based on the relationship between compound concentration and peak area and subsequently used to quantify the compounds in the phloem and xylem exudates of castor bean seedlings. The linear regression equations for the individual compounds are presented in Table 8. It should be noted that the HPLC-UV method used in this study was not subjected to a full matrix-specific validation for phloem and xylem exudates. Matrix-specific limits of detection (LOD), limits of quantification (LOQ), recovery, and matrix-effect studies were not performed. In addition, compound assignments in the plant exudate samples were mainly based on the retention times of authentic standards under the same HPLC conditions and were not further confirmed by LC-MS or HRMS in the exudate matrices. Therefore, the reported concentrations in phloem and xylem sap should be interpreted as preliminary HPLC-based estimates.

## 4. Conclusions

A total of 34 amino acid and amino acid ester conjugates based on a thiasporine A-derived phenylthiazole scaffold were synthesized and evaluated for their antifungal activity and systemic mobility. The amino acid conjugates **9a-9p** generally exhibited stronger antifungal activity than their corresponding ester derivatives **8a–8r**, suggesting that the free carboxyl group, together with associated changes in physicochemical properties, may contribute to improved activity. In the mobility assays, both intact conjugates and the unconjugated carbxylic acid precursor (compound 3) were detected in plant sap, and the total values were therefore described as total conjugate-derived material. Because the present experiments cannot distinguish whether the intact conjugates were transported before hydrolysis or hydrolyzed before/during uptake and translocation, the mobility data should be interpreted cautiously. These results suggest that amino acid conjugation can modulate the amount and form of compound-derived material recovered from plant sap, while the mobility of intact conjugates requires further investigation.

## Figures and Tables

**Table 1:** Structure and melting point of target compounds **8a-8r** and **9a-9p**.

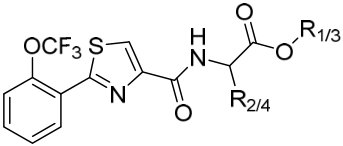				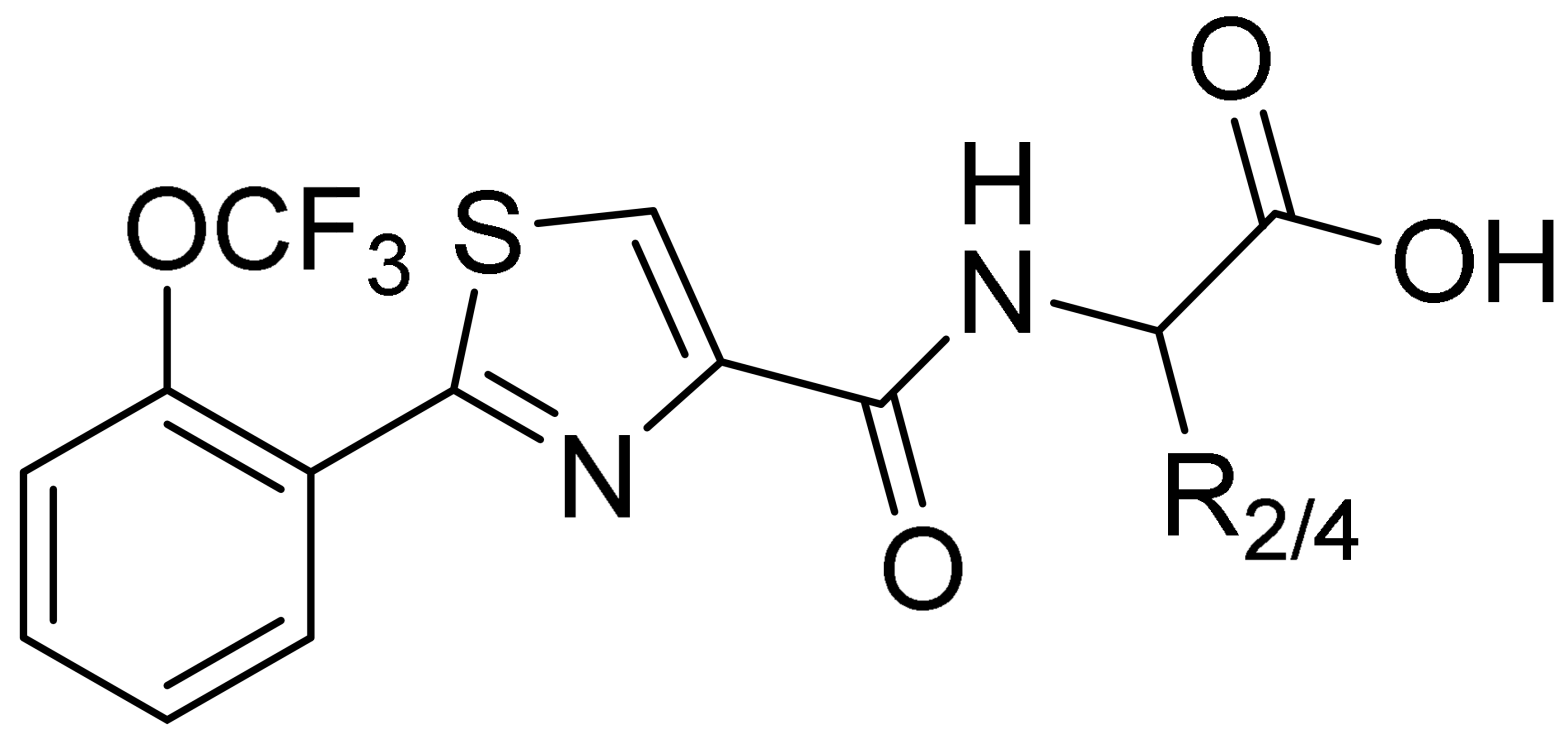		
Compd.	R_1/3_	R_2/4_	M.p (°C)	Compd.	R_2/4_	M.p (°C)
**8a**	CH_3_	H	NT	**9a**	H	>280 °C
**8b**	CH_3_	CH_3_	98.8~100.3 °C	**9b**	CH_3_	156.4~160 °C
**8c**	C_2_H_5_	CH_3_	75.8~78.1 °C	**9c**	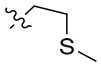	143.2~145.9 °C
**8d**	CH_3_	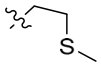	91~92.8 °C	**9d**	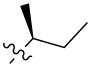	185.3~187.1 °C
**8e**	CH_3_	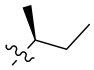	101.2~103.4 °C	**9e**	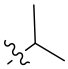	148.5~149.3 °C
**8f**	CH_3_	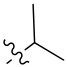	98.5~102.1 °C	**9f**	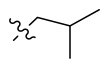	150.7~152.5 °C
**8g**	CH_3_	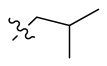	102.2~104.8 °C	**9g**	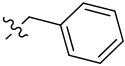	161.8~163.8 °C
**8h**	CH_3_	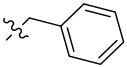	108.2~111.7 °C	**9h**	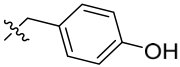	269.7~272.3 °C
**8i**	CH_3_	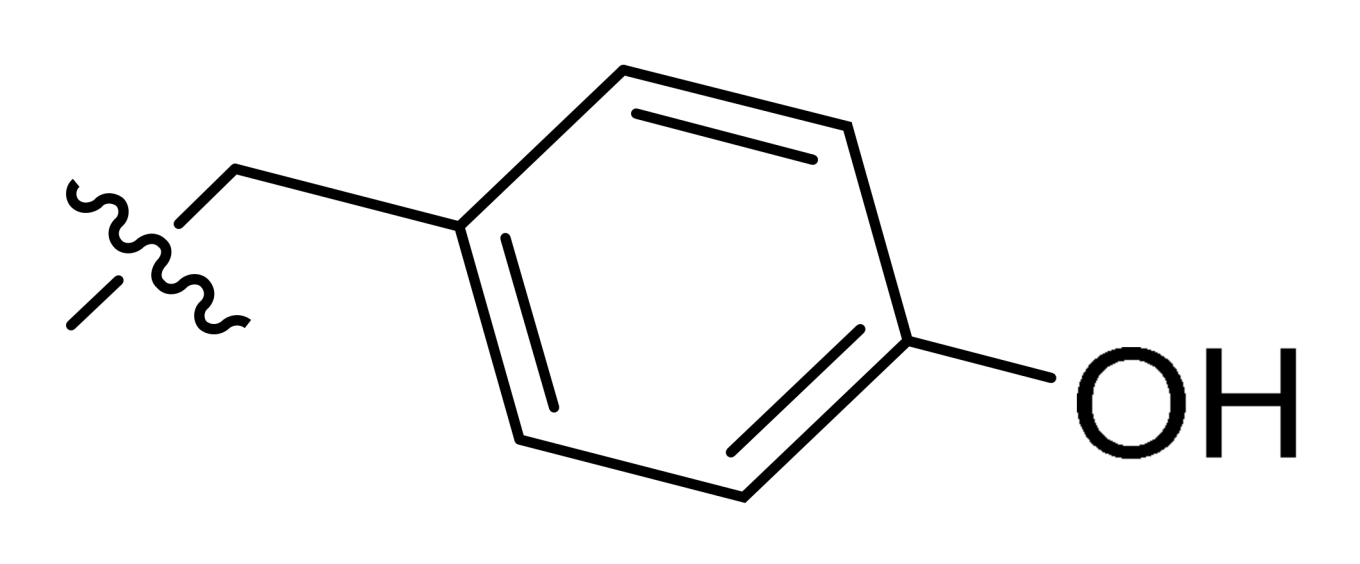	144.4~147.1 °C	**9i**	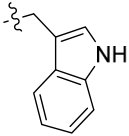	146.8~147.8 °C
**8j**	CH_3_	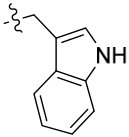	140.3~142.3 °C	**9j**	CH_3_	161.7~163.4 °C
**8k**	CH_3_	CH_3_	110.3~113.8 °C	**9k**	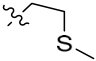	145.6~147.3 °C
**8l**	C_2_H_5_	CH_3_	97.6~99.7 °C	**9l**	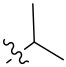	171.2~173.2 °C
**8m**	CH_3_	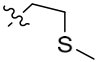	81.5~83.2 °C	**9m**	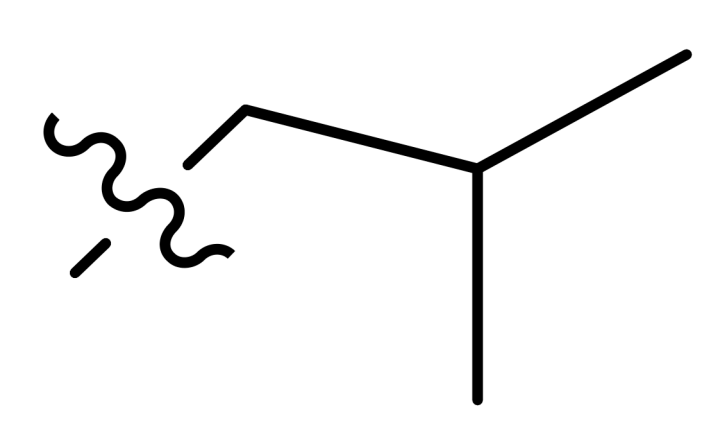	141.0~144.2 °C
**8n**	CH_3_	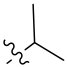	93.2~95.1 °C	**9n**	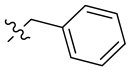	159.6~162.5 °C
**8o**	CH_3_	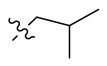	105.1~107.5 °C	**9o**	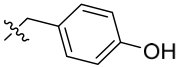	159.5~160.7 °C
**8p**	CH_3_	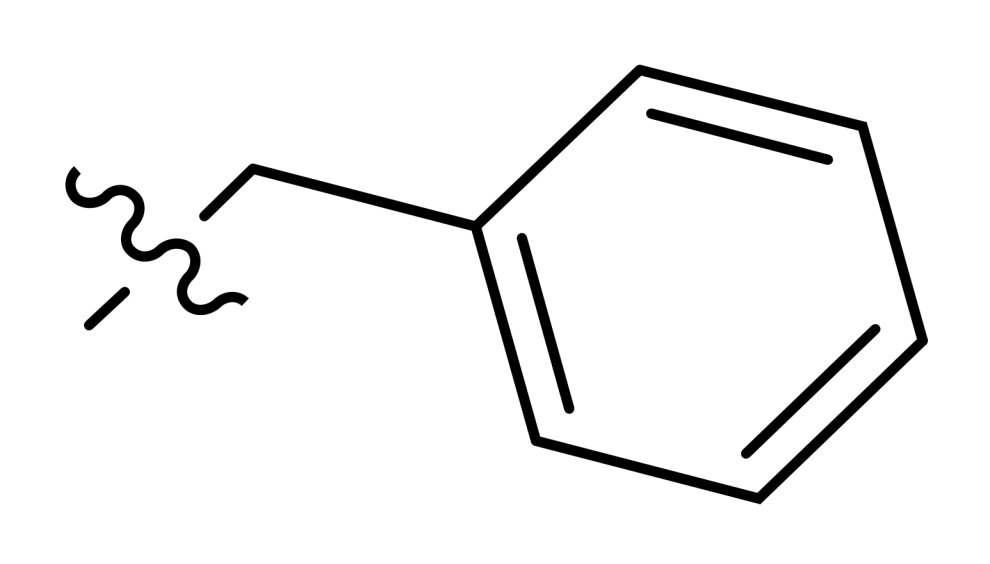	111.4~113.9 °C	**9p**	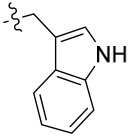	179.9~181.4 °C
**8q**	CH_3_	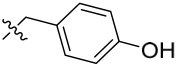	146.3~148.1 °C			
**8r**	CH_3_	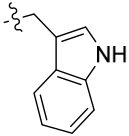	91~92.8 °C			

**Table 2:** Antifungal activities of target compounds **8a-8r** and **9a-9p** against five fungi.

Compound	Conjugated Amino Acid	Inhibitory Ratio Under 200 μmol/L (%)				
		*R. solani*	*S. rolfsii*	*P. citricarpa*	*S. sclerotiorum*	*P. parasitica*
**8a**	Gly methyl ester	57.33 ± 0.01	67.28 ± 0.02	69.54 ± 0.01	50.34 ± 0.01	NA
**8b**	L-Ala methyl ester	19.72 ± 0.03	29.73 ± 0.01	41.34 ± 0.01	27.45 ± 0.00	0.73 ± 0.01
**8c**	L-Ala ethyl ester	43.66 ± 0.01	26.01 ± 0.01	51.96 ± 0.01	31.37 ± 0.01	NA
**8d**	L-Met methyl ester	35.22 ± 0.01	NA	23.94 ± 0.03	13.04 ± 0.01	16.12 ± 0.01
**8e**	L-Ile methyl ester	17.33 ± 0.01	18.75 ± 0.01	30.77 ± 0.01	29.05 ± 0.02	0.35 ± 0.01
**8f**	L-Val methyl ester	38.03 ± 0.03	15.88 ± 0.03	16.2 ± 0.02	5.56 ± 0.01	6.18 ± 0.01
**8g**	L-Leu methyl ester	36.56 ± 0.01	22.85 ± 0.01	32.11 ± 0.01	15.38 ± 0.01	12.17 ± 0.01
**8h**	L-Phe methyl ester	18.01 ± 0.01	NA	16.34 ± 0.01	7.36 ± 0.01	NA
**8i**	L-Tyr methyl ester	12.67 ± 0.02	NA	4.62 ± 0.02	4.73 ± 0.03	14.6 ± 0.01
**8j**	L-Trp methyl ester	1.00 ± 0.01	6.25 ± 0.01	11.38 ± 0.01	11.15 ± 0.01	NA
**8k**	D-Ala methyl ester	31.69 ± 0.01	33.78 ± 0.02	53.07 ± 0.01	25.16 ± 0.01	10.91 ± 0.01
**8l**	D-Ala ethyl ester	37.32 ± 0.01	35.47 ± 0.01	67.06 ± 0.02	33.33 ± 0.01	8.36 ± 0.01
**8m**	D-Met methyl ester	36.29 ± 0.01	6.39 ± 0.00	32.28 ± 0.01	26.42 ± 0.02	14.47 ± 0.02
**8n**	D-Val methyl ester	32.39 ± 0.01	10.81 ± 0.01	15.36 ± 0.01	22.55 ± 0.01	8.00 ± 0.01
**8o**	D-Leu methyl ester	43.01 ± 0.02	10.58 ± 0.01	46.2 ± 0.01	18.39 ± 0.01	7.24 ± 0.01
**8p**	D-Phe methyl ester	21.77 ± 0.01	4.74 ± 0.03	23.94 ± 0.00	13.04 ± 0.01	8.22 ± 0.01
**8q**	D-Tyr methyl ester	28.67 ± 0.01	5.15 ± 0.01	9.54 ± 0.02	18.92 ± 0.02	4.51 ± 0.03
**8r**	D-Trp methyl ester	29.67 ± 0.01	6.99 ± 0.01	14.46 ± 0.01	4.39 ± 0.01	NA
**9a**	Gly	10.33 ± 0.02	75.00 ± 0.01	22.46 ± 0.01	13.51 ± 0.01	21.18 ± 0.01
**9b**	L-Ala	19.72 ± 0.01	41.89 ± 0.03	39.66 ± 0.01	16.34 ± 0.02	16.36 ± 0.02
**9c**	L-Met	40.33 ± 0.01	20.96 ± 0.01	43.08 ± 0.02	22.3 ± 0.01	8.33 ± 0.01
**9d**	L-Ile	66.33 ± 0.01	50.74 ± 0.01	79.08 ± 0.01	60.47 ± 0.01	40.28 ± 0.01
**9e**	L-Val	57.04 ± 0.01	NA	62.85 ± 0.01	38.24 ± 0.02	21.09 ± 0.01
**9f**	L-Leu	73.66 ± 0.02	14.23 ± 0.01	82.82 ± 0.03	65.82 ± 0.01	40.13 ± 0.00
**9g**	L-Phe	69.35 ± 0.01	23.72 ± 0.02	89.86 ± 0.01	68.56 ± 0.00	41.45 ± 0.01
**9h**	L-Tyr	29.58 ± 0.01	38.18 ± 0.01	47.77 ± 0.01	17.97 ± 0.01	26.18 ± 0.01
**9i**	L-Trp	29.67 ± 0.03	12.87 ± 0.01	51.69 ± 0.01	32.09 ± 0.01	48.96 ± 0.01
**9j**	D-Ala	6.34 ± 0.01	36.15 ± 0.01	45.81 ± 0.01	10.78 ± 0.02	45.82 ± 0.03
**9k**	D-Met	26.00 ± 0.01	14.34 ± 0.02	71.38 ± 0.02	43.24 ± 0.01	39.93 ± 0.01
**9l**	D-Val	41.55 ± 0.01	27.03 ± 0.01	80.45 ± 0.01	41.18 ± 0.01	56.73 ± 0.01
**9m**	D-Leu	71.77 ± 0.01	13.5 ± 0.01	76.31 ± 0.00	63.88 ± 0.01	66.78 ± 0.01
**9n**	D-Phe	74.46 ± 0.02	20.07 ± 0.02	91.55 ± 0.01	68.56 ± 0.02	66.12 ± 0.01
**9o**	D-Tyr	18.31 ± 0.01	32.43 ± 0.01	44.97 ± 0.01	24.51 ± 0.01	21.82 ± 0.02
**9p**	D-Trp	38.33 ± 0.02	NA	49.85 ± 0.01	41.22 ± 0.01	37.5 ± 0.01
Thiasporine A		39.78 ± 0.01	15.69 ± 0.01	32.56 ± 0.02	6.69 ± 0.00	35.86 ± 0.01
Thifluzamide		91.40 ± 0.01	95.62 ± 0.01	29.30 ± 0.01	78.60 ± 0.02	9.87 ± 0.01

Note: Values are the mean ± SD of three replicates. NA indicates a negative calculated inhibition rate, the colony diameter of the treatment group was greater than that of the corresponding control group under the tested conditions.

**Table 3:** The EC_50_ values of compounds against *R. solani*, *P. citricarpa* and *S. sclerotiorum* (μmol/L).

Compound	Conjugated Amino Acid	*R. solani*	*P. citricarpa*	*S. sclerotiorum*
**9d**	L-Ile	62.44 ± 0.89	69.56 ± 3.74	59.42 ± 1.06
**9f**	L-Leu	78.96 ± 3.90	98.69 ± 5.58	54.94 ± 1.44
**9g**	L-Phe	42.75 ± 0.90	72.42 ± 1.31	58.68 ± 0.47
**9m**	D-Leu	47.59 ± 2.39	59.69 ± 1.68	76.01 ± 0.87
**9n**	D-Phe	46.87 ± 2.59	54.97 ± 1.37	35.62 ± 0.92
Thifluzamide		0.47 ± 0.34	443.14 ± 4.23	0.11 ± 0.02

Note: Values are the mean ± SD of three replicates.

**Table 4:** Concentration of compounds in the phloem sap of castor bean.

Conjugate	Conjugated Amino Acid	Concentration in Phloem Sap (μmol/L)		
		Conjugate	Compound 3	Total Conjugate- Derived Material
**9a**	Gly	16.63 ± 1.59	38.03 ± 2.15	54.66 ± 3.74
**9b**	L-Ala	1.25 ± 0.03	78.29 ± 2.40	79.54 ± 2.43
**9c**	L-Met	3.77 ± 0.93	36.56 ± 2.62	40.33 ± 3.55
**9d**	L-Ile	2.63 ± 0.21	38.53 ± 1.90	41.16 ± 2.11
**9e**	L-Val	1.66 ± 0.05	86.8 ± 2.74	88.46 ± 2.79
**9f**	L-Leu	1.14 ± 0.15	9.13 ± 0.29	10.27 ± 0.44
**9g**	L-Phe	1.22 ± 0.02	5.65 ± 0.21	6.87 ± 0.23
**9h**	L-Tyr	-	3.73 ± 0.25	3.73 ± 0.25
**9i**	L-Trp	-	-	-
**9j**	D-Ala	39.10 ± 1.32	-	39.10 ± 1.32
**9k**	D-Met	8.41 ± 0.51	-	8.41 ± 0.51
**9l**	D-Val	36.12 ± 0.43	-	36.12 ± 0.43
**9m**	D-Leu	2.60 ± 0.08	-	2.60 ± 0.08
**9n**	D-Phe	0.57 ± 0.03	-	0.57 ± 0.03
**9o**	D-Tyr	3.15 ± 0.13	-	3.15 ± 0.13
**9p**	D-Trp	-	-	-
Thiasporine A		124.02 ± 1.92	-	124.02 ± 1.93

Note: Values are the mean ± SD of three replicates. “-” indicates that no corresponding chromatographic peak was detected under the analytical conditions used. **Compound 3** refers to the unconjugated carboxylic acid precursor. Total conjugate-derived material represents the sum of the intact conjugate and **compound 3** detected in the sap and should not be interpreted as the concentration of the intact conjugate.

**Table 5:** Predicted p*K*a and Log*K*ow values of compounds.

Conjugate	Conjugated Amino Acid	p*K*a	Log*K*ow	Conjugate	Conjugated Amino Acid	p*K*a	Log*K*ow
**9a**	Gly	3.18	2.63	**9j**	D-Ala	3.23	2.72
**9b**	L-Ala	3.23	2.72	**9k**	D-Met	3.05	3.01
**9c**	L-Met	3.05	3.01	**9l**	D-Val	3.24	3.26
**9d**	L-Ile	3.26	3.21	**9m**	D-Leu	3.25	3.5
**9e**	L-Val	3.24	3.26	**9n**	D-Phe	3.03	3.62
**9f**	L-Leu	3.25	3.5	**9o**	D-Tyr	3.18	3.96
**9g**	L-Phe	3.03	3.62	**9p**	D-Trp	3.21	4.22
**9h**	L-Tyr	3.18	3.96	**Thiasporine A**		3.41	2.09
**9i**	L-Trp	3.21	4.22				

**Table 6:** Concentration of compounds in xylem sap of castor bean.

Conjugate	Conjugated Amino Acid	Concentration in Xylem Sap (μmol/L)		
		Conjugate	Intermediate 3	Total Concentration
**9a**	Gly	-	10.78 ± 0.02	10.78 ± 0.02
**9b**	L-Ala	-	5.38 ± 0.06	5.38 ± 0.06
**9c**	L-Met	-	13.52 ± 2.97	13.52 ± 2.97
**9d**	L-Ile	-	7.97 ± 0.13	7.97 ± 0.13
**9e**	L-Val	-	8.46 ± 0.25	8.46 ± 0.25
**9f**	L-Leu	19.15 ± 0.56	11.19 ± 0.49	30.34 ± 1.05
**9g**	L-Phe	-	1.82 ± 0.02	1.82 ± 0.02
**9h**	L-Tyr	-	1.24 ± 0.20	1.24 ± 0.20
**9i**	L-Trp	-	-	-
**9j**	D-Ala	6.55 ± 1.42	-	6.55 ± 1.42
**9k**	D-Met	1.25 ± 0.06	-	1.25 ± 0.06
**9l**	D-Val	7.38 ± 0.61	-	7.38 ± 0.61
**9m**	D-Leu	0.85 ± 0.21	-	0.85 ± 0.21
**9n**	D-Phe	-	-	-
**9o**	D-Tyr	-	-	-
**9p**	D-Trp	-	-	-
Thiasporine A		3.53 ± 0.48	-	3.53 ± 0.48

Note: Values are the mean ± SD of three replicates. “-” indicates that no corresponding chromatographic peak was detected under the analytical conditions used. **Compound 3** refers to the unconjugated carboxylic acid precursor.

**Table 7:** Gradient elution conditions for HPLC analysis.

Thiasporine A			Compounds		
Time/Min	CH_3_OH/%	H_2_O ^a^/%	Time/Min	CH_3_OH/%	H_2_O ^a^/%
0	50	50	0	65	35
6	50	50	6	65	35
8	60	40	8	70	30
12	65	35	12	85	15
14	65	35	14	85	15
14.1	60	40	14.1	75	25
20	60	40	20	75	25

Note: “^a^” contains 0.1% HCOOH.

**Table 8:** Calibration curves and retention times of the tested compounds.

Compound	Retention Time (Min)	Linear Equation	R^2^
**9a**	9.503	*y* = 10,302*x* + 15.33	0.9983
**9b**	12.71	*y* = 10,017*x* − 3.3479	0.9999
**9c**	15.33	*y* = 9157.3*x* + 5.5987	0.9992
**9d**	17.198	*y* = 6661.4*x* + 8.3178	0.9992
**9e**	15.83	*y* = 8839.6*x* + 9.1969	0.9998
**9f**	16.972	*y* = 9938.0*x* + 0.7116	0.9997
**9g**	16.65	*y* = 9551.2*x* + 11.181	0.9994
**9h**	12.63	*y* = 8589.4*x* + 7.8567	0.9998
**9i**	15.93	*y* = 13,412*x* + 25.765	0.9998
**9j**	12.733	*y* = 9439.5*x* + 11.424	0.9998
**9k**	15.233	*y* = 8757.8*x* + 12.379	0.9997
**9l**	15.872	*y* = 8123*x* − 10.604	0.9997
**9m**	16.955	*y* = 20,805*x* + 24.5	0.9993
**9n**	16.657	*y* = 9570.4*x* + 6.1934	0.9999
**9o**	12.607	*y* = 9739.6*x* + 17.592	0.9997
**9p**	15.983	*y* = 11,227*x* − 1.5881	0.9999
**Intermediate 3**	11.303	*y* = 7559.2*x* + 7.0273	0.9991
**Thiasporine A**	12.842	*y* = 4154.3*x* + 5.0639	0.9999

## Data Availability

The original contributions presented in this study are included in the article/Supplementary Material. Further inquiries can be directed to the corresponding authors.

## Supplementary Materials

The following supporting information can be downloaded at: https://www.mdpi.com/article/10.3390/molecules31183241/s1.

## References

[1] Dayan F.E., Cantrell C.L., Duke S.O. (2009). Natural products in crop protection. Bioorg. Med. Chem..

[2] Cragg G.M., Newman D.J. (2013). Natural products: A continuing source of novel drug leads. Biochim. Biophys. Acta BBA-Gen. Subj..

[3] Fu P., MacMillan J.B. (2015). Thiasporines A-C, thiazine and thiazole derivatives from a marine-derived *Actinomycetospora chlora*. J. Nat. Prod..

[4] Seitz T., Fu P., Haut F.L., Adam L., Habicht M., Lentz D., MacMillan J.B., Christmann M. (2016). One-pot synthesis of 5-hydroxy-4H-1,3-thiazin-4-ones: Structure revision, synthesis, and NMR shift dependence of Thiasporine A. Org. Lett..

[5] Vaaland I.C., Lindbäck E., Sydnes M.O. (2019). Total synthesis of anithiactins A-C and Thiasporine A. Tetrahedron Lett..

[6] Xu X., Deng L., Nie L., Chen Y., Liu Y., Xie R., Li Z. (2019). Discovery of 2-phenylthiazole-4-carboxylic acid, a novel and potent scaffold as xanthine oxidase inhibitors. Bioorg. Med. Chem. Lett..

[7] Mohammadi-Farani A., Foroumadi A., Kashani M.R., Aliabadi A. (2014). *N*-Phenyl-2-p-tolylthiazole-4-carboxamide derivatives: Synthesis and cytotoxicity evaluation as anticancer agents. Iran. J. Basic Med. Sci..

[8] Chen S.S., Zhu X., Shi J.C., Wang M.M., Hu C.Y., Liao C., Zhang Y., Li J.K. (2022). Design, synthesis and fungicidal activities of Thiasporine A analogues. Chin. J. Pestic. Sci..

[9] Guichard B., Wu H.X., La Camera S., Hu R., Marivingt-Mounir C., Chollet J.F. (2022). Synthesis, phloem mobility and induced plant resistance of synthetic salicylic acid amino acid or glucose conjugates. Pest Manag. Sci..

[10] Lei Z.W., Wang J., Mao G.L., Wen Y.J., Tian Y.Q., Wu H.X., Li Y.F., Xu H.H. (2014). Glucose positions affect the phloem mobility of glucose-fipronil conjugates. J. Agric. Food Chem..

[11] Wang B.F., Yang C., Jiang X.Y., Wen Y.J., Tian Y.Q., Zhao C., Xu H.H. (2022). Design of new glycosyl-O-fipronil conjugates with improved hydrolysis efficiency assisted by molecular simulations. Pest Manag. Sci..

[12] Zheng S.J., Lin X.M., Wu H.X., Zhao C., Xu H.H. (2020). Synthesis, bioactivities and phloem uptake of dipeptide-chlorantraniliprole derivatives. BMC Chem..

[13] Chollet J.F., Delétage C., Faucher M., Miginiac L., Bonnemain J.L. (1997). Synthesis and structure–activity relationships of some pesticides with an α-amino acid function. Biochim. Biophys. Acta (BBA)-Gen. Subj..

[14] Jiang D.X., Lu X.L., Shan H., Zhang X.B., Xu H.H. (2009). A new derivative of fipronil: Effect of adding a glycinyl group to the 5-amine of pyrazole on phloem mobility and insecticidal activity. Pestic. Biochem. Physiol..

[15] Xie Y., Zhao J.L., Wang C.W., Yu A.X., Liu N., Chen L., Lin F., Xu H.H. (2016). Glycinergic-fipronil uptake is mediated by an amino acid carrier system and induces the expression of amino acid transporter genes in Ricinus communis seedlings. J. Agric. Food Chem..

[16] Wu H.X., Marhadour S., Lei Z.W., Yang W., Marivingt-Mounir C., Bonnemain J.L., Chollet J.F. (2018). Vectorization of agrochemicals: Amino acid carriers are more efficient than sugar carriers to translocate phenylpyrrole conjugates in the Ricinus system. Environ. Sci. Pollut. Res..

[17] Niu J.F., Nie D.Y., Yu D.Y., Wu Q.L., Yu L.H., Yao Z.L., Du X.Y., Li J.K. (2017). Synthesis, fungicidal activity and phloem mobility of phenazine-1-carboxylic acid-alanine conjugates. Pestic. Biochem. Physiol..

[18] Hsu F.C., Kleier D.A., Melander W.R. (1988). Phloem mobility of xenobiotics: II. Bioassay testing of the unified mathematical model. Plant Physiol..

[19] Kleier D.A. (1988). Phloem mobility of xenobiotics: I. Mathematical model unifying the weak acid and intermediate permeability theories. Plant Physiol..

[20] Hsu F.C., Kleier D.A. (1990). Phloem mobility of xenobiotics. III. Sensitivity of unified model to plant parameters and application to patented chemical hybridizing agents. Weed Sci..

[21] Hart J.J., DiTomaso J.M., Linscott D.L., Kochian L.V. (1992). Transport interactions between paraquat and polyamines in roots of intact maize seedlings. Plant Physiol..

[22] Denis M.H., Delrot S. (1993). Carrier-mediated uptake of glyphosate in broad bean (Vicia faba) via a phosphate transporter. Physiol. Plant..

[23] Byers T.L., Kameji R., Rannels D.E., Pegg A.E. (1987). Multiple pathways for uptake of paraquat, methylglyoxal bis(guanylhydrazone), and polyamines. Am. J. Physiol..

[24] Marhadour S., Wu H.X., Yang W., Marivingt-Mounir C., Bonnemain J.L., Chollet J.F. (2017). Vectorisation of agrochemicals via amino acid carriers: Influence of the spacer arm structure on the phloem mobility of phenylpyrrole conjugates in the *Ricinus* system. Pest Manag. Sci..

