# Supplementary material for: Synthesis, Antifungal Activity, and Plant Systemic Mobility of Amino Acid Conjugates of a Thiasporine A-Derived Phenylthiazole Scaffold
Source: Molecules. 2026 Sep 14;31(18):3241. doi: 10.3390/molecules31183241 (PMC13609501; doi:10.3390/molecules31183241)
Supplement: Supplementary file 1 [file molecules-31-03241-s001.zip › molecules-4550705-supplementary.pdf]

Supplementary Material

Synthesis, Antifungal Activity, and Plant Systemic Mobility of Amino Acid Conjugates of a Thiasporine A-Derived Phenylthiazole Scaffold

Li Li [1,2], Shunshun Chen [3], Panpan Yin [1,2], Xintao Huang [1], Jing Li [1], Ya'xuan Xiao [1], Changxu Lu [1], Lin Li [1], Yang Wang [1], Xiang Zhu [1,2,3], Linhua Yu [1,2,*] and Junkai Li [1,2,*]

[1] Hubei Key Laboratory of Waterlogging Disaster and Agricultural Use of Wetland, College of Agriculture, Yangtze University, Jingmi Road 88, Jingzhou 434025, China

[2] Institute of Pesticides, Yangtze University, Jingmi Road 88, Jingzhou 434025, China

[3] State Key Laboratory of Green Pesticide, Guizhou University, Guiyang 550025, China

* Correspondence: linhuayu531@sina.com (L.Y.); junkaili@sina.com (J.L.)

Table of Contents

1. The structure and data of target compounds **8a~8r** and **9a~9p** (**Figure S1- Figure S102**) **1**

2. The structure and data of target compounds (8a~8r, 9a~9p)

8a: yellow liquid; yield 75%, ^{1}H NMR (400 MHz, DMSO-d_6) δ 9.04 (t, J = 6.0 Hz, 1H), 8.56 (dd, J = 1.6, 8.0 Hz, 1H), 8.50 (s, 1H), 7.71 – 7.64 (m, 1H), 7.64 – 7.56 (m, 2H), 4.09 (d, J = 6.0 Hz, 2H), 3.68 (s, 3H); ^{13}C NMR (101 MHz, DMSO-d_6) δ 170.67, 161.15, 160.42, 149.45, 145.80, 132.69, 130.32, 128.41, 126.77, 125.60, 121.84, 121.48, 52.28, 41.35. HRMS(ESI) calcd for $C_{14}H_{12}F_3N_2O_4S^+$ [M+H]$^+$: 361.0464, found 361.0465.

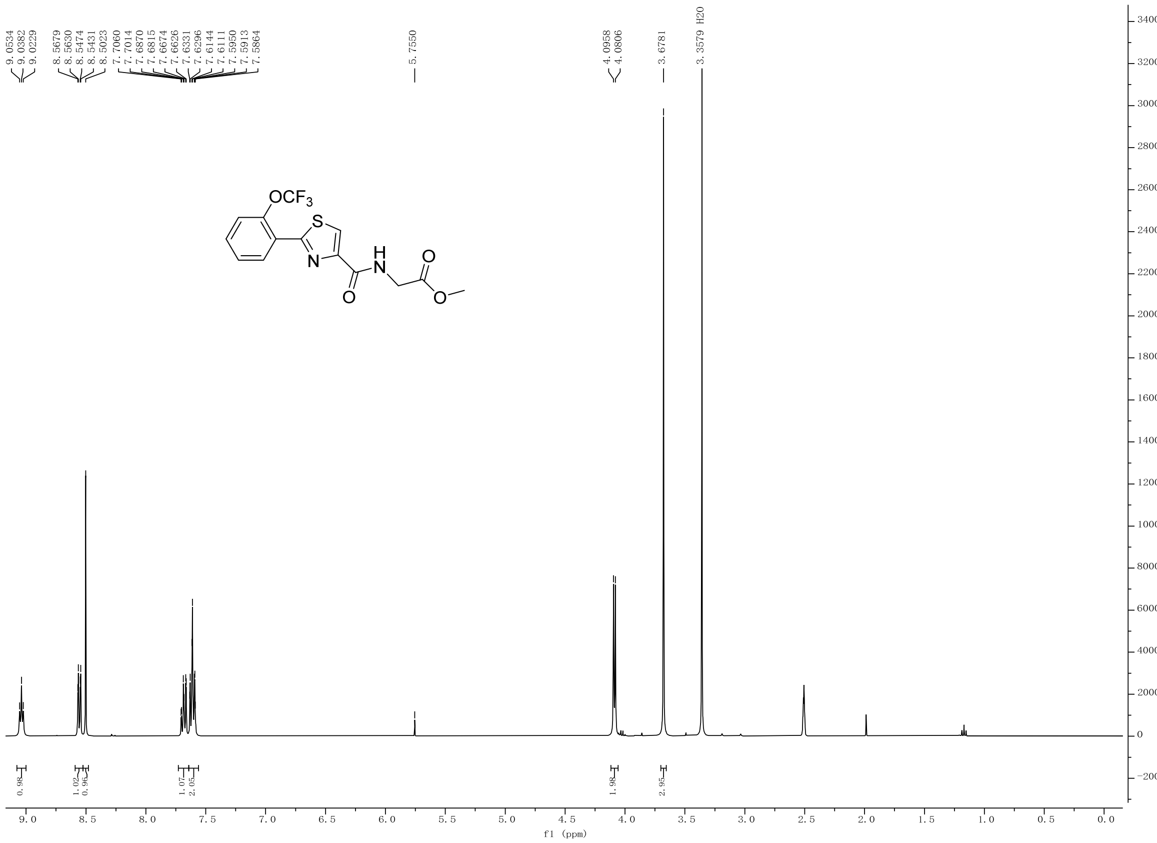

Figure S1. ^{1}H-NMR Spectrum of compound **8a**

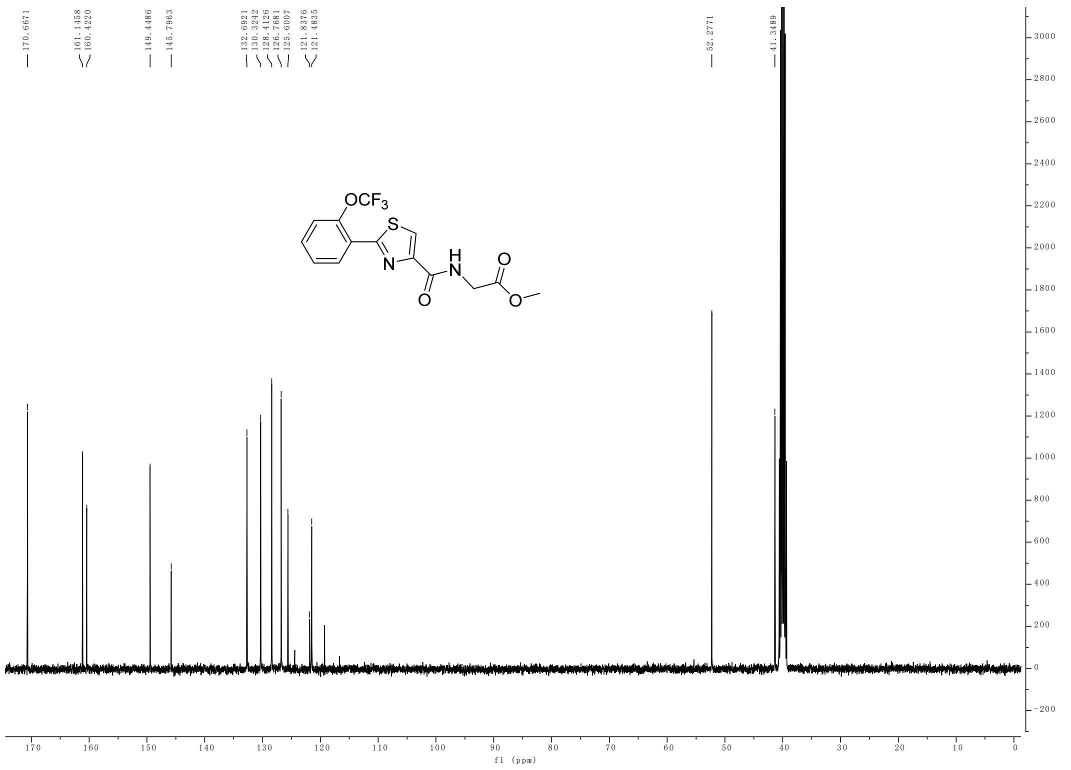

Figure S2. ^{13}C-NMR Spectrum of compound **8a**

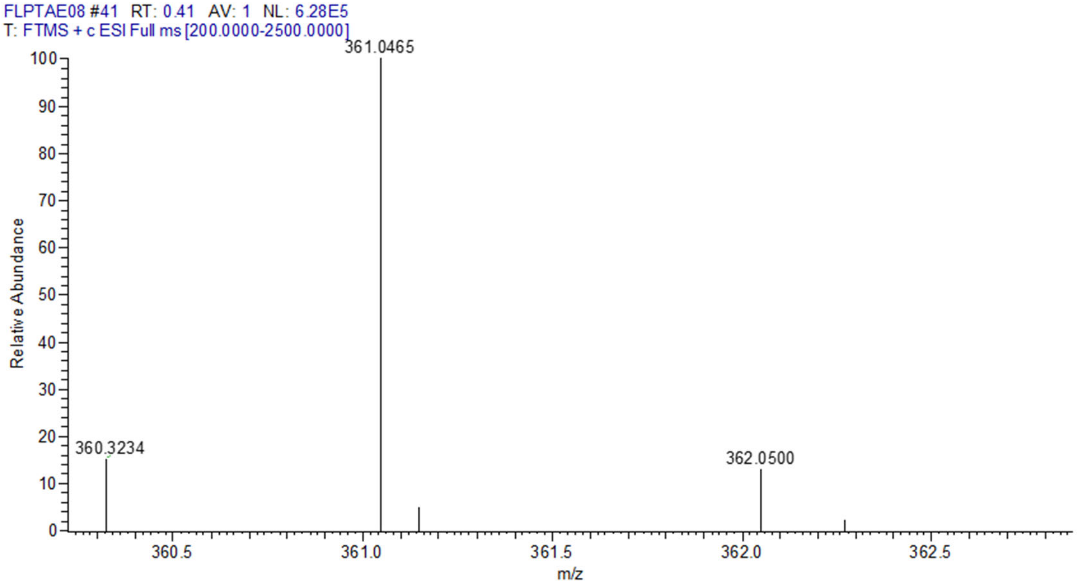

Figure S3. HRMS Spectrum of compound **8a**

8b: white solid; yield 73%, melting point 98.8~100.3 °C; ^{1}H NMR (400 MHz, DMSO-d_6) δ 8.88 (d, J = 7.6 Hz, 1H), 8.66 – 8.57 (m, 1H), 8.50 (s, 1H), 7.74 – 7.65 (m, 1H), 7.65 – 7.56 (m, 2H), 4.66 – 4.53 (m, 1H), 3.67 (s, 3H), 1.47 (d, J = 7.2 Hz, 3H);

^{13}C NMR (101 MHz, DMSO-d_6) δ 173.21, 160.55, 160.41, 149.48, 145.78, 132.64, 130.55, 128.38, 126.83, 125.61, 121.45, 120.33, 52.48, 48.17, 17.39. HRMS(ESI) calcd for $C_{15}H_{14}F_3N_2O_4S^+$ [M+H]$^+$: 375.0621, found 375.0621.

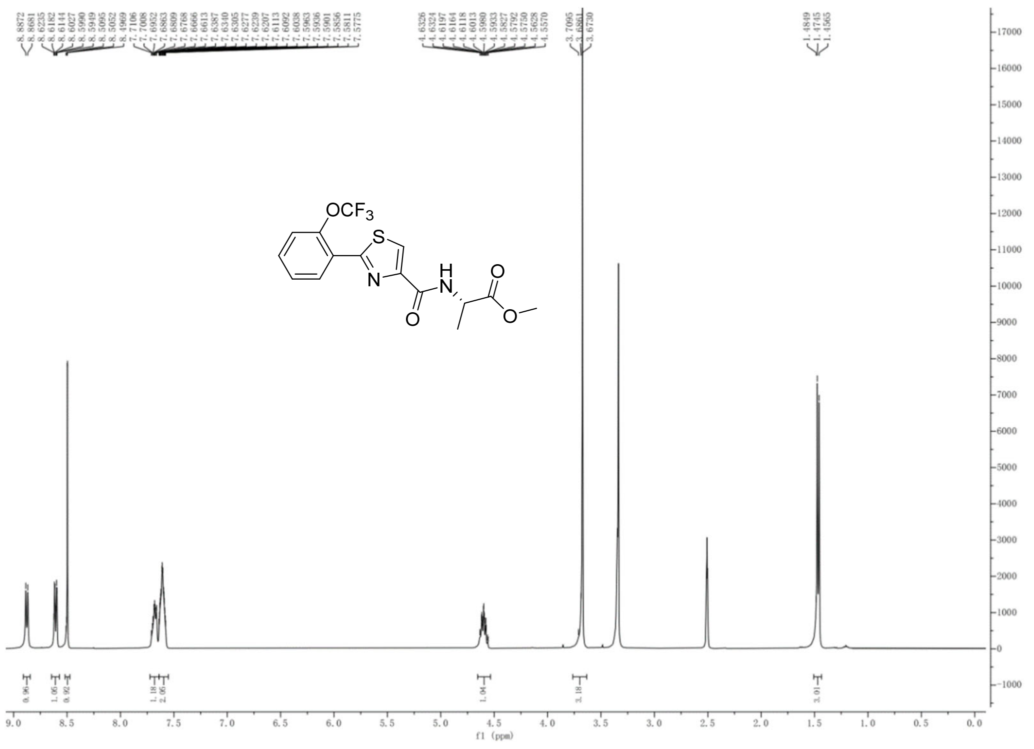

Figure S4. ^1H-NMR Spectrum of compound **8b**

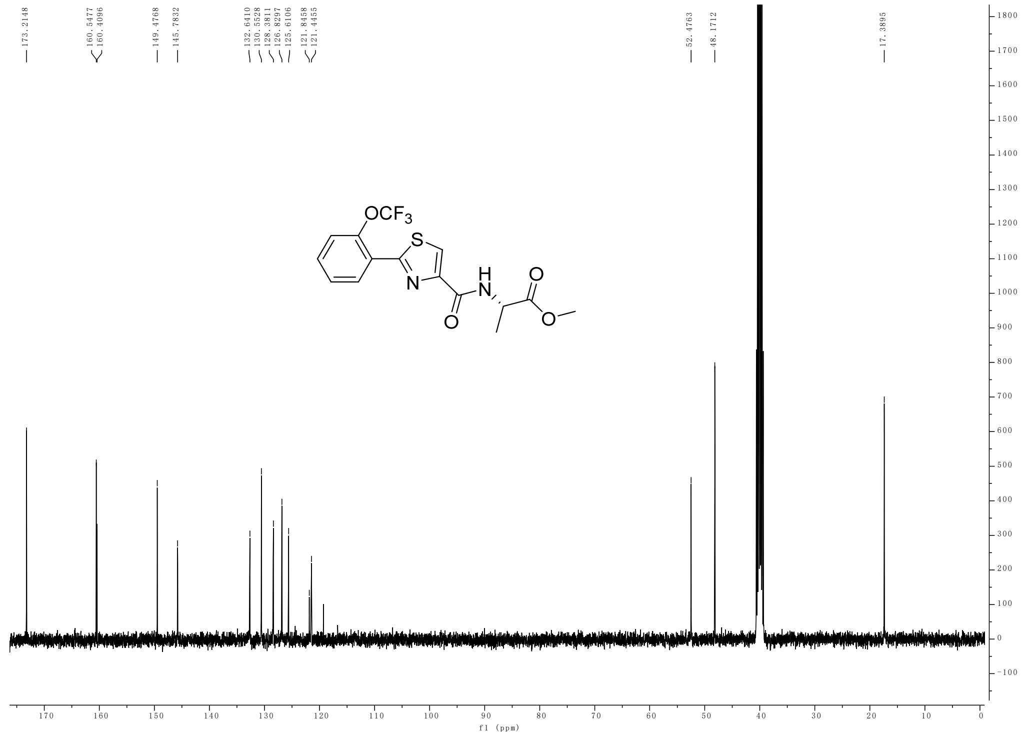

Figure S5. ^{13}C-NMR Spectrum of compound **8b**

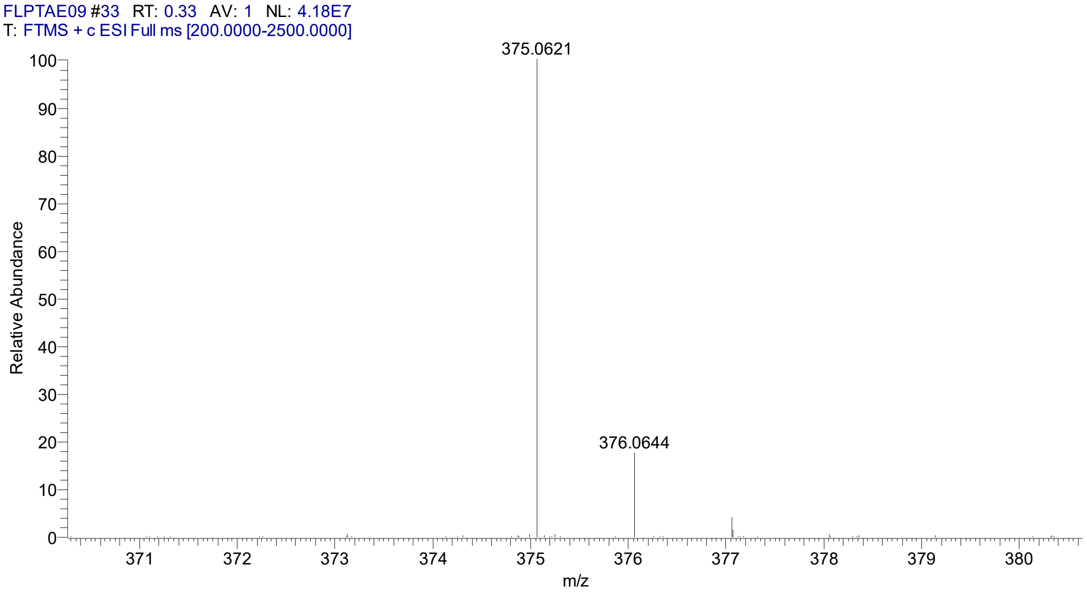

Figure S6. HRMS Spectrum of compound **8b**

8c: white solid; yield 78%, melting point 75.8~78.1°C; ^{1}H NMR (400 MHz, DMSO-*d*6) δ 8.84 (d, J = 7.6 Hz, 1H), 8.62 (dd, J = 1.6, 7.8 Hz, 1H), 8.51 (s, 1H), 7.74 – 7.66 (m, 1H), 7.66 – 7.56 (m, 2H), 4.57 (t, J = 7.4 Hz, 1H), 4.15 (dd, J = 3.6, 7.1 Hz, 2H), 1.48 (d, J = 7.2 Hz, 3H), 1.22 (t, J = 7.2 Hz, 3H); ^{13}C NMR (101 MHz, DMSO-*d*6) δ 172.73, 160.56, 160.41, 149.52, 145.77, 132.66, 130.55, 128.41, 126.79, 125.62, 121.85, 121.47, 61.11, 48.32, 17.39, 14.52. HRMS(ESI) calcd for $C_{16}H_{16}F_3N_2O_4S^+$ [M+H]$^+$: 389.0777, found 389.0772.

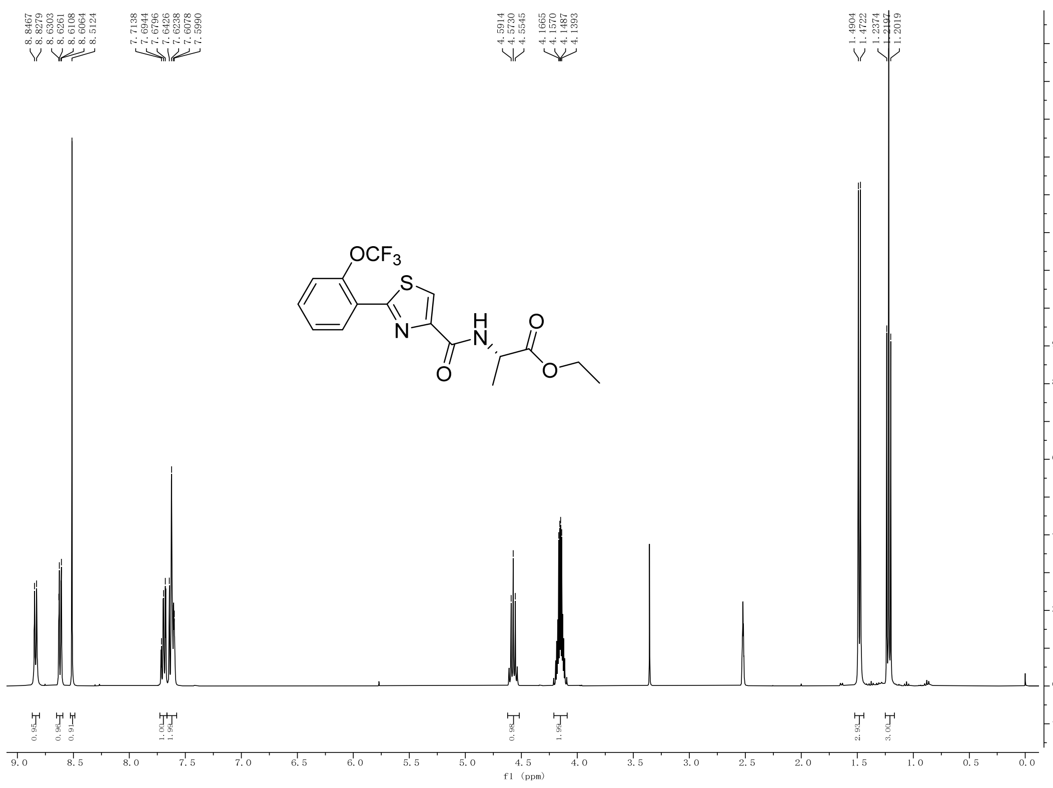

Figure S7. ^{1}H-NMR Spectrum of compound **8c**

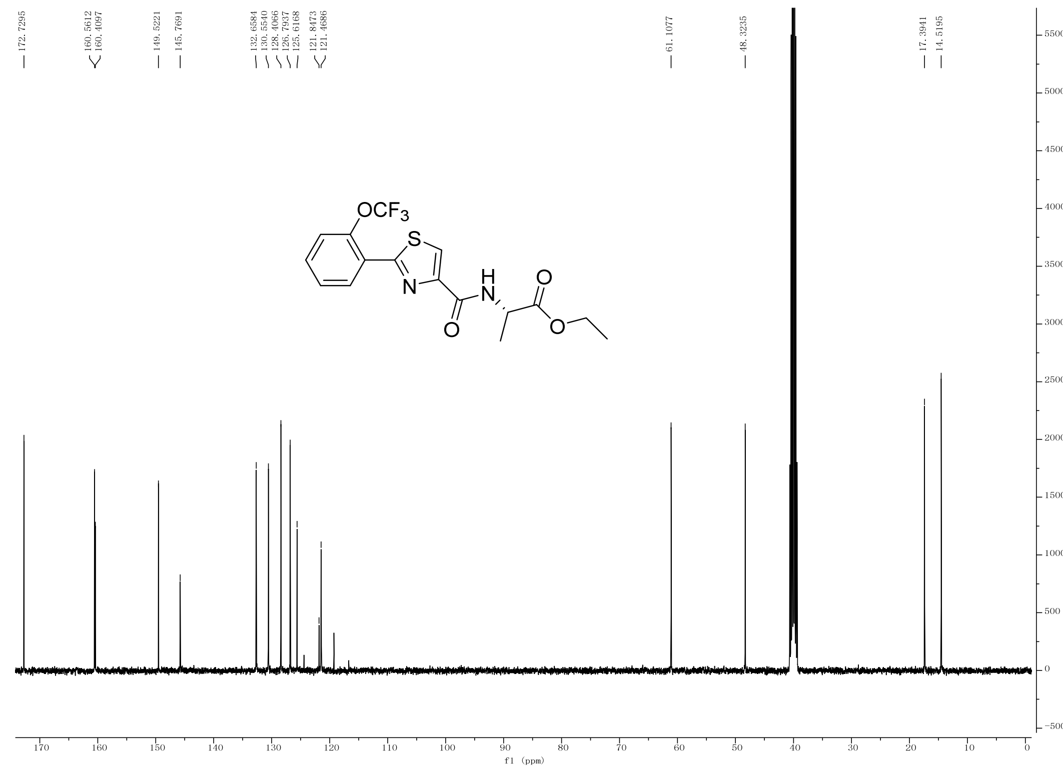

Figure S8. ^{13}C-NMR Spectrum of compound **8c**

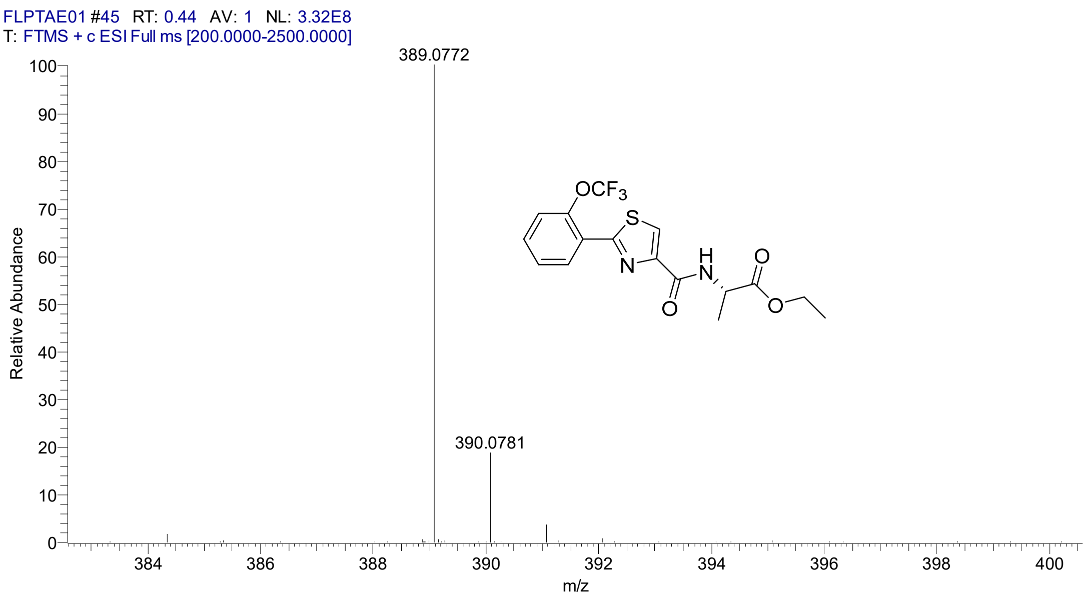

Figure S9. HRMS Spectrum of compound **8c**

8d: white solid; yield 75%, melting point91 ~ 92.8°C; ^{1}H NMR (400 MHz, DMSO-d_6) δ 8.91 (d, J = 8.0 Hz, 1H), 8.62 (dd, J = 1.6, 8.0 Hz, 1H), 8.52 (s, 1H), 7.73 – 7.67 (m, 1H), 7.66 – 7.58 (m, 2H), 4.76 – 4.65 (m, 1H), 3.69 (s, 3H), 2.66 – 2.53 (m, 2H), 2.24 – 2.13 (m, 2H), 2.08 (s, 3H); ^{13}C NMR (101 MHz, DMSO-d_6) δ 172.53, 161.01, 160.44, 149.41, 145.79, 132.70, 130.58, 128.44, 126.99, 125.63, 121.85, 121.51, 52.60, 51.56, 30.55, 30.36, 15.04. HRMS(ESI) calcd for $C_{17}H_{18}F_3N_2O_4S_2^+$ $[M+H]^+$: 435.0655, found 435.0652.

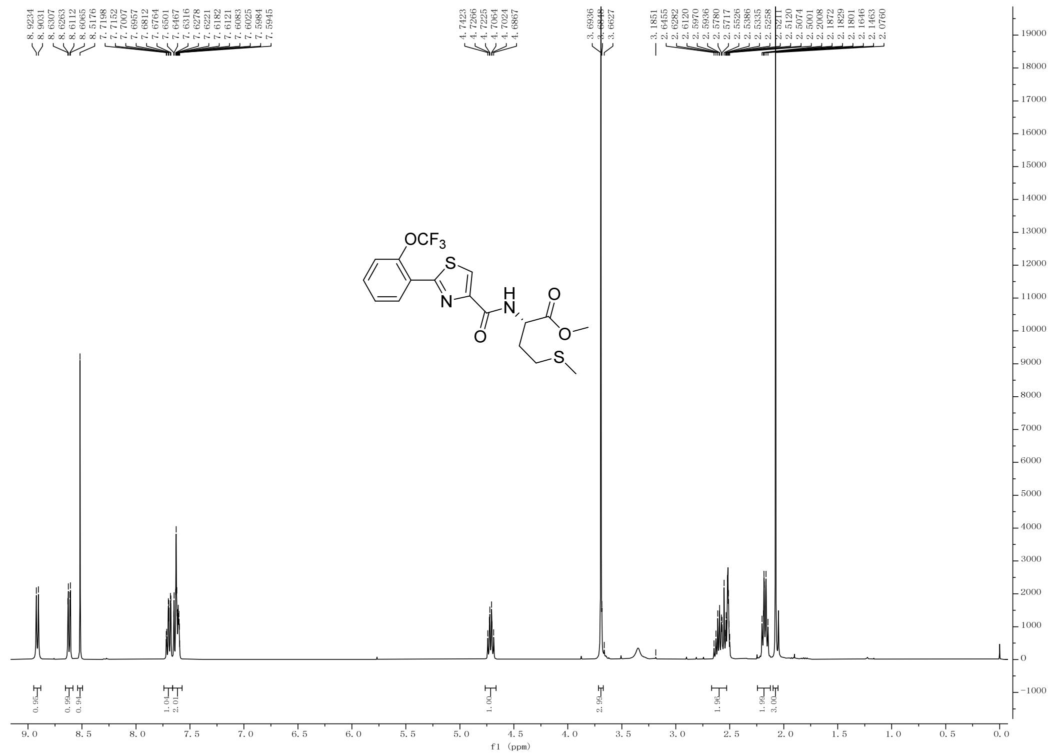

Figure S10. ^{1}H-NMR Spectrum of compound **8d**

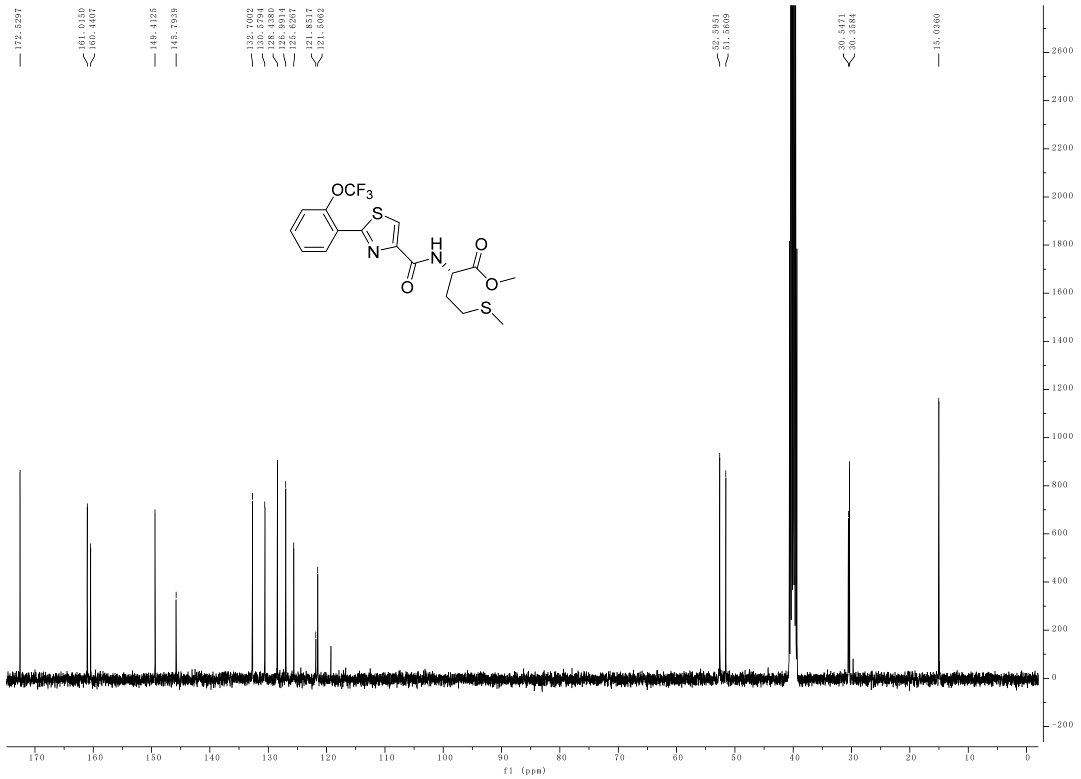

Figure S11. ^{13}C-NMR Spectrum of compound **8d**

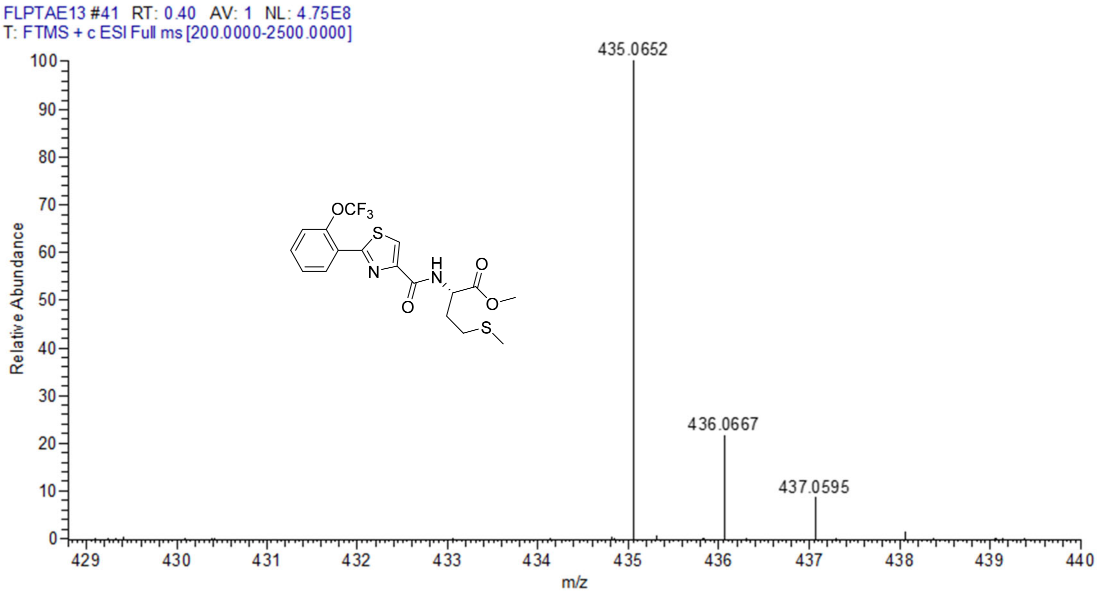

Figure S12. HRMS Spectrum of compound **8d**

8e: white solid; yield 83%, melting point101.2~103.4 °C; ^{1}H NMR (400 MHz, DMSO-d_6) δ 8.55 – 8.49 (m, 2H), 8.41 (d, J = 8.4 Hz, 1H), 7.72 – 7.65 (m, 1H), 7.65 – 7.56 (m, 2H), 4.49 (dd, J = 6.8, 8.4 Hz, 1H), 3.69 (s, 3H), 2.12 – 1.96 (m, 1H), 1.58 –

1.44 (m, 1H), 1.32 – 1.16 (m, 1H), 0.95 – 0.85 (m, 6H); ^{13}C NMR (101 MHz, DMSO-d_6) δ 172.24, 160.84, 160.71, 149.35, 145.69, 132.72, 130.66, 128.57, 126.99, 125.59, 121.86, 121.68, 56.92, 52.35, 36.58, 25.42, 15.90, 11.39. HRMS(ESI) calcd for $C_{18}H_{20}F_3N_2O_4S^+$ [M+H]$^+$: 417.1090, found 417.1085.

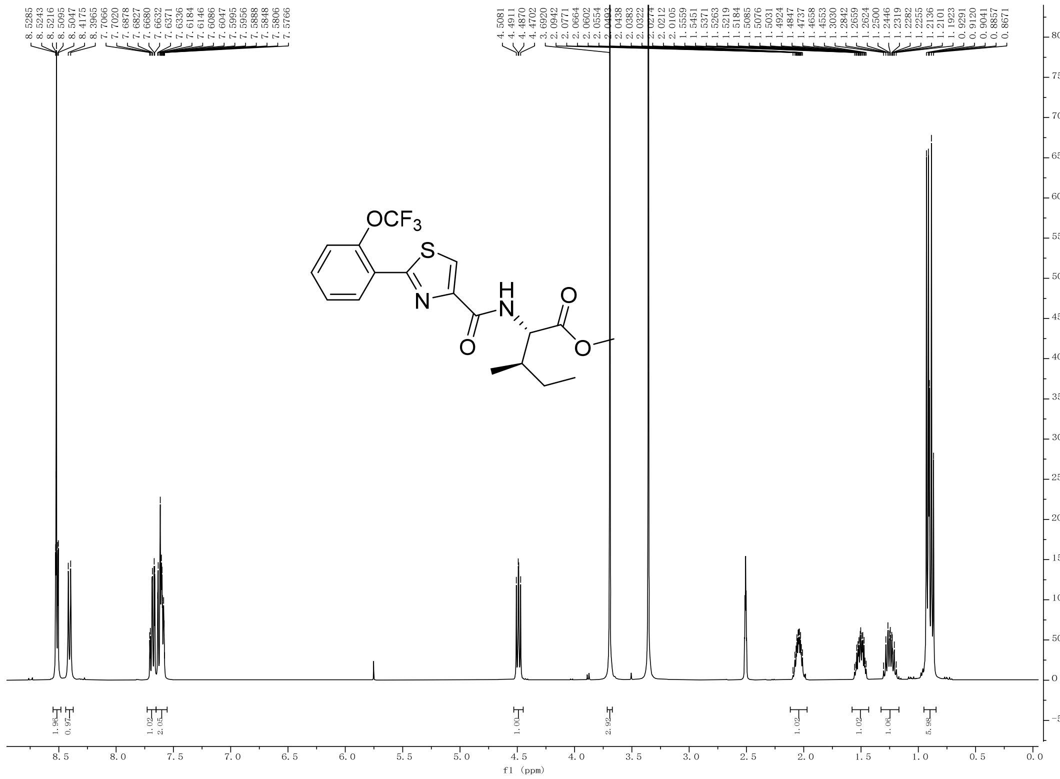

Figure S13. ^{1}H-NMR Spectrum of compound **8e**

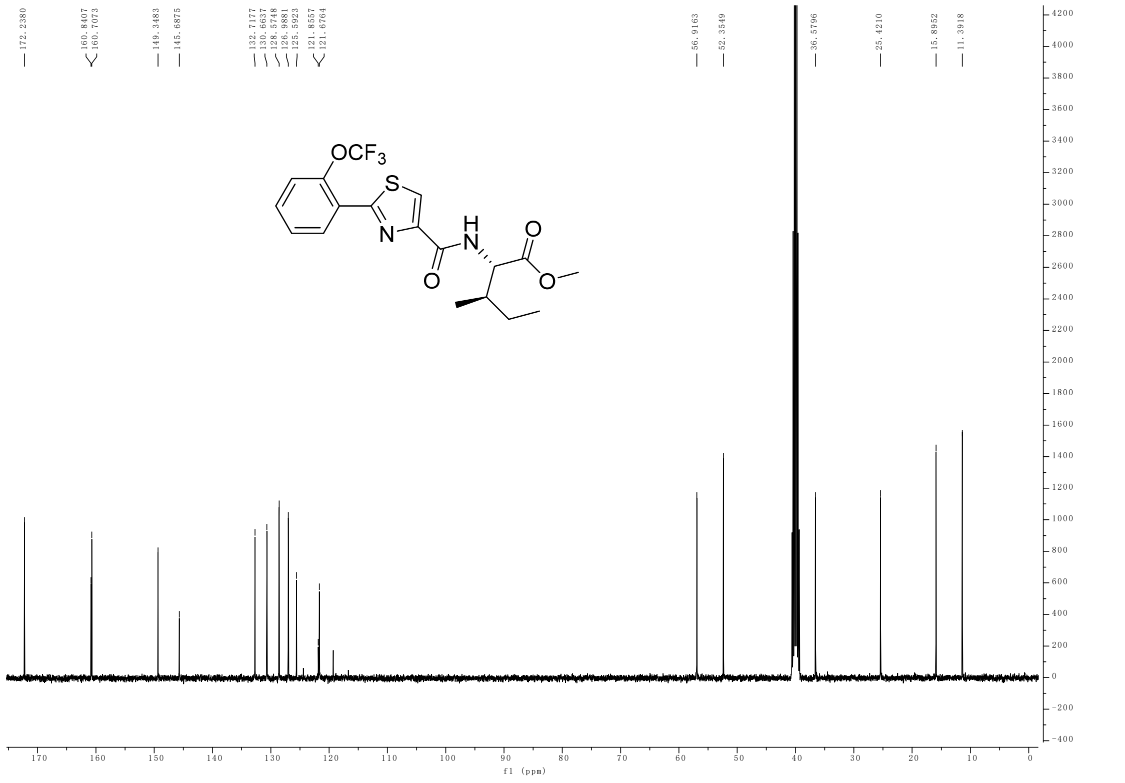

Figure S14. ^{13}C-NMR Spectrum of compound **8e**

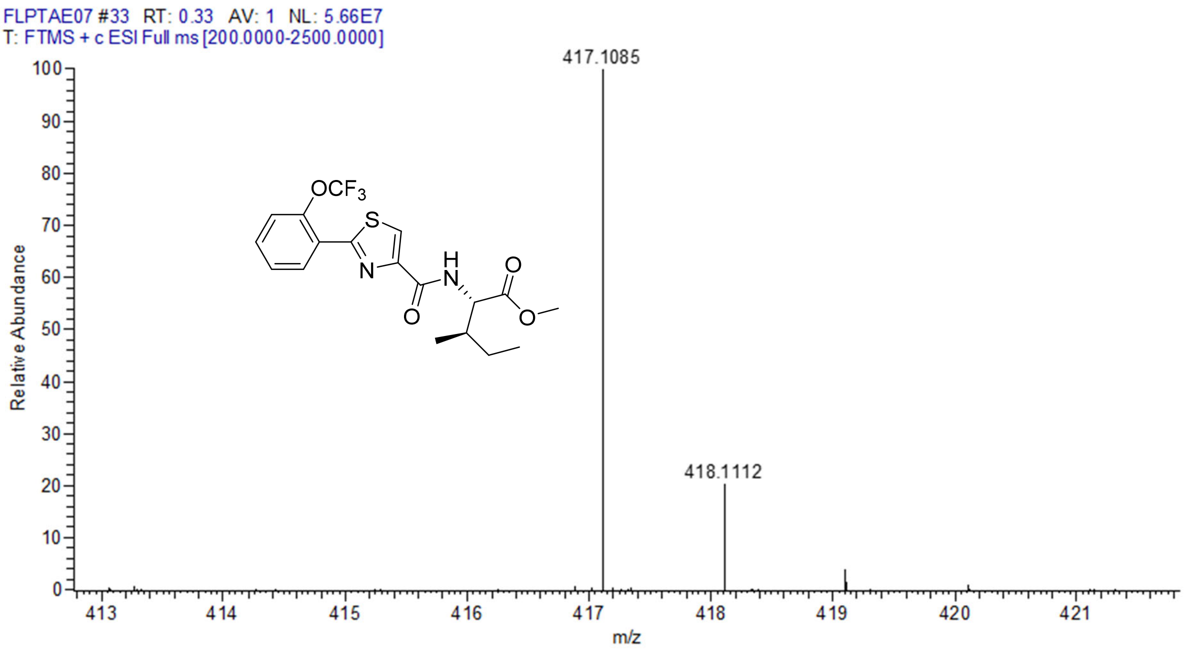

Figure S15. HRMS Spectrum of compound **8e**

8f: white solid; yield 80%, melting point 98.5~102.1 ℃; ^{1}H NMR (400 MHz, DMSO-d_6) δ 8.53 (d, J = 4.8 Hz, 2H), 8.41 (d, J = 8.4 Hz, 1H), 7.73 – 7.67 (m, 1H), 7.63 (m, J = 1.6, J = 7.8 Hz, 2H), 4.42 (dd, J = 6.8, J = 8.4 Hz, 1H), 3.70 (s, 3H), 2.26 (dd, J = 6.8, J = 13.6 Hz, 1H), 0.97 (dd, J = 5.6, J = 6.8 Hz, 6H); ^{13}C NMR (101 MHz,

DMSO-d_6) δ 172.20, 160.80, 149.38, 145.70, 132.75, 130.70, 128.61, 126.98, 125.62, 121.87, 121.72, 119.30, 58.06, 52.40, 30.37, 19.54, 18.94. HRMS(ESI) calcd for $C_{17}H_{18}F_3N_2O_4S^+$ $[M+H]^+$: 403.0934, found 403.0929.

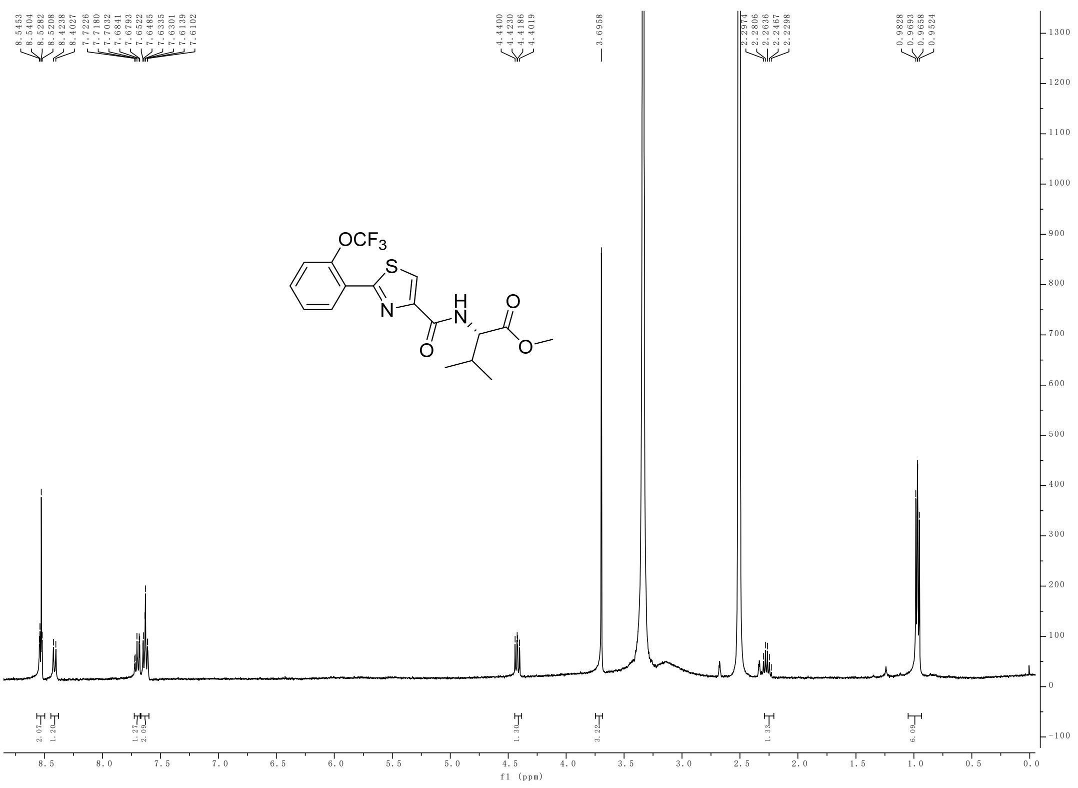

Figure S16. ^{1}H-NMR Spectrum of compound **8f**

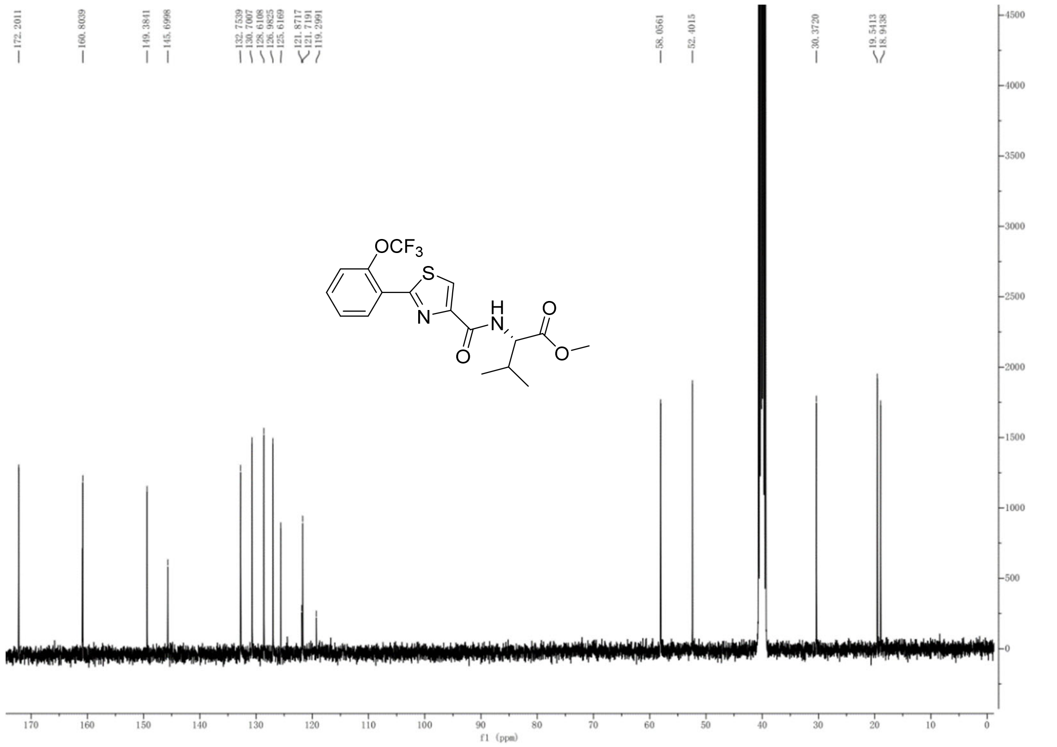

Figure S17. ^{13}C-NMR Spectrum of compound **8f**

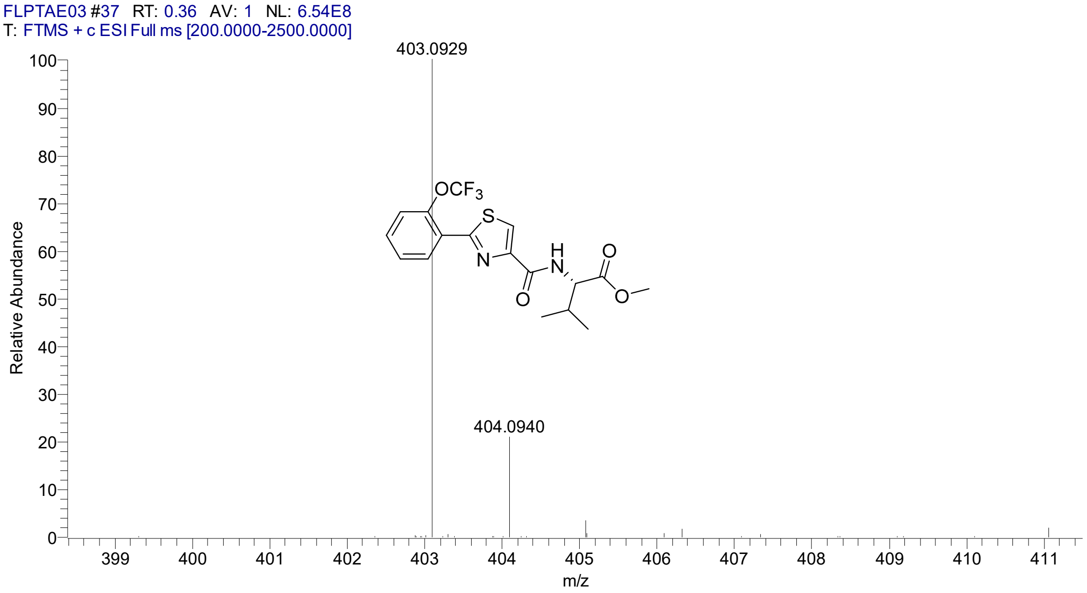

Figure S18. HRMS Spectrum of compound **8f**

8g: white solid; yield 72%, melting point 102.2~104.8°C; ^{1}H NMR (400 MHz, DMSO-*d*6) δ 8.79 (d, J = 8.4 Hz, 1H), 8.62 (dd, J = 1.6, 8.0 Hz, 1H), 8.50 (s, 1H), 7.74 – 7.65 (m, 1H), 7.66 – 7.57 (m, 2H), 4.65 – 4.56 (m, 1H), 3.67 (s, 3H), 1.95 – 1.81 (m, 1H), 1.72 – 1.61 (m, 2H), 0.96 – 0.87 (m, 6H); ^{13}C NMR (101 MHz, DMSO-*d*6) δ 173.16, 160.87, 160.48, 149.45, 145.77, 132.70, 130.64, 128.47, 126.94, 125.63, 121.85, 121.53, 119.28, 52.49, 50.81, 24.86, 23.35, 21.68. HRMS(ESI) calcd for $C_{18}H_{20}F_3N_2O_4S^+$ $[M+H]^+$: 417.1090, found 417.1087.

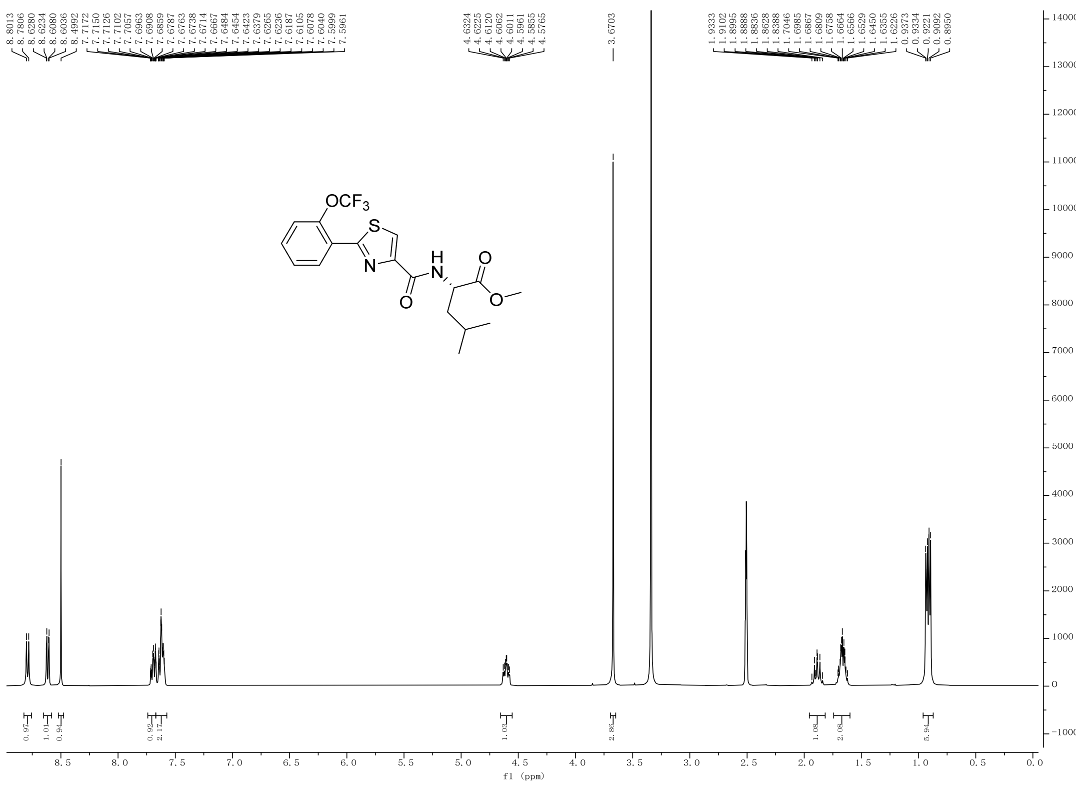

Figure S19. ^{1}H-NMR Spectrum of compound **8g**

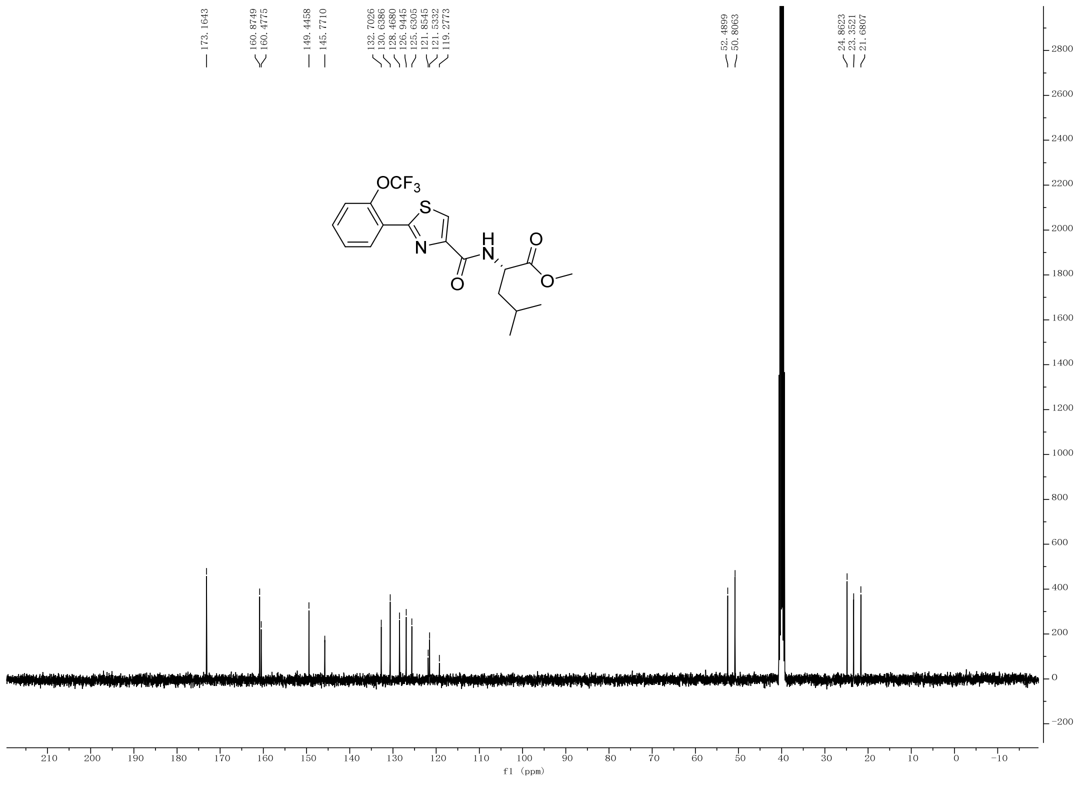

Figure S20. ^{13}C-NMR Spectrum of compound **8g**

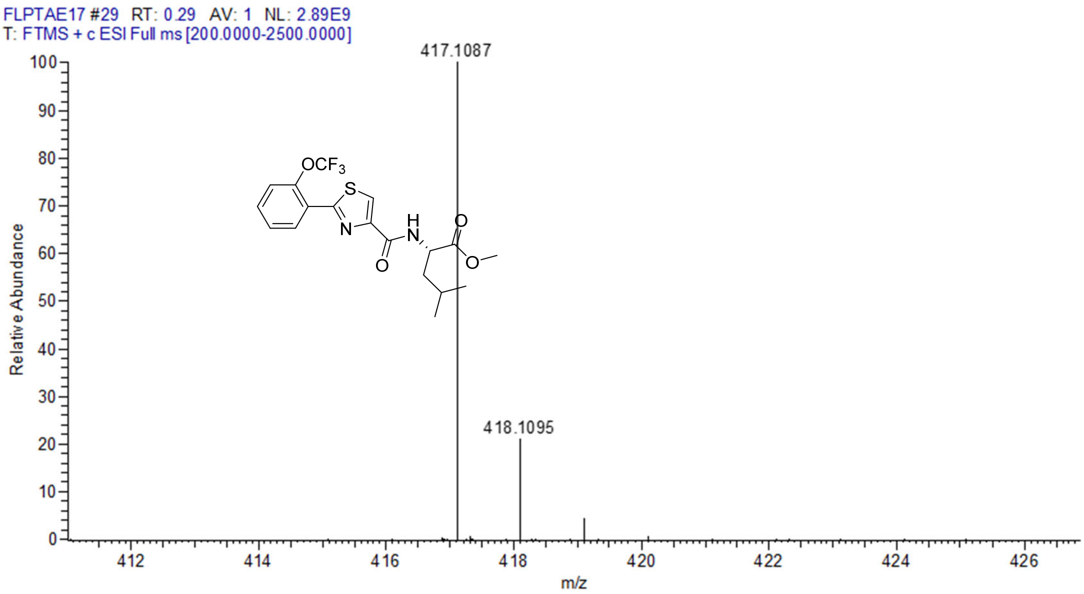

Figure S21. HRMS Spectrum of compound **8g**

8h: white solid; yield 81%, melting point 108.2~111.7 °C; ^{1}H NMR (400 MHz, DMSO-d_6) δ 8.76 (d, J = 8.4 Hz, 1H), 8.54 (dd, J = 2.0, 7.8 Hz, 1H), 8.45 (s, 1H), 7.75 – 7.58 (m, 3H), 7.33 – 7.25 (m, 4H), 7.24 – 7.17 (m, 1H), 4.85 – 4.75 (m, 1H), 3.67 (s, 3H), 3.29 – 3.17 (m, 2H); ^{13}C NMR (101 MHz, DMSO-d_6) δ 172.18, 160.62, 160.47, 149.27, 145.78, 137.82, 132.75, 130.43, 129.58, 128.79, 128.49, 127.07, 126.91, 125.57, 121.55, 119.26, 53.89, 52.57, 36.71. HRMS(ESI) calcd for $C_{21}H_{18}F_3N_2O_4S^+$ $[M+H]^+$: 451.0934, found 451.0930.

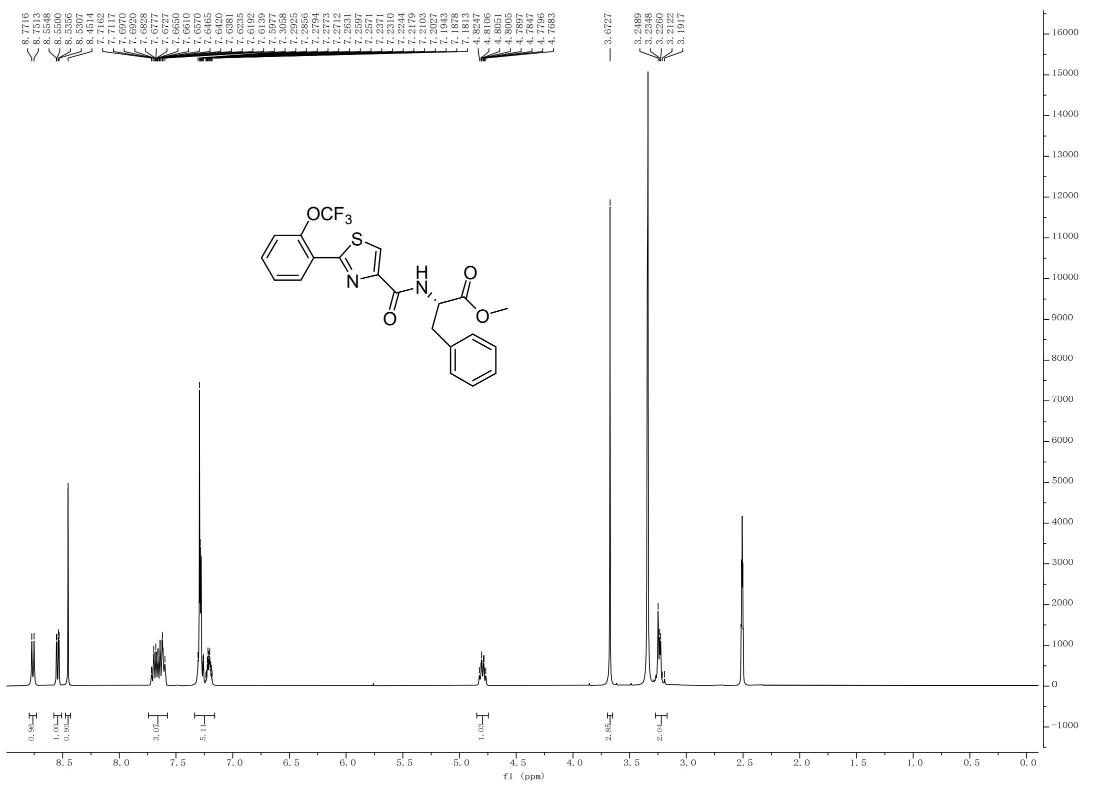

Figure S22. ^{1}H-NMR Spectrum of compound **8h**

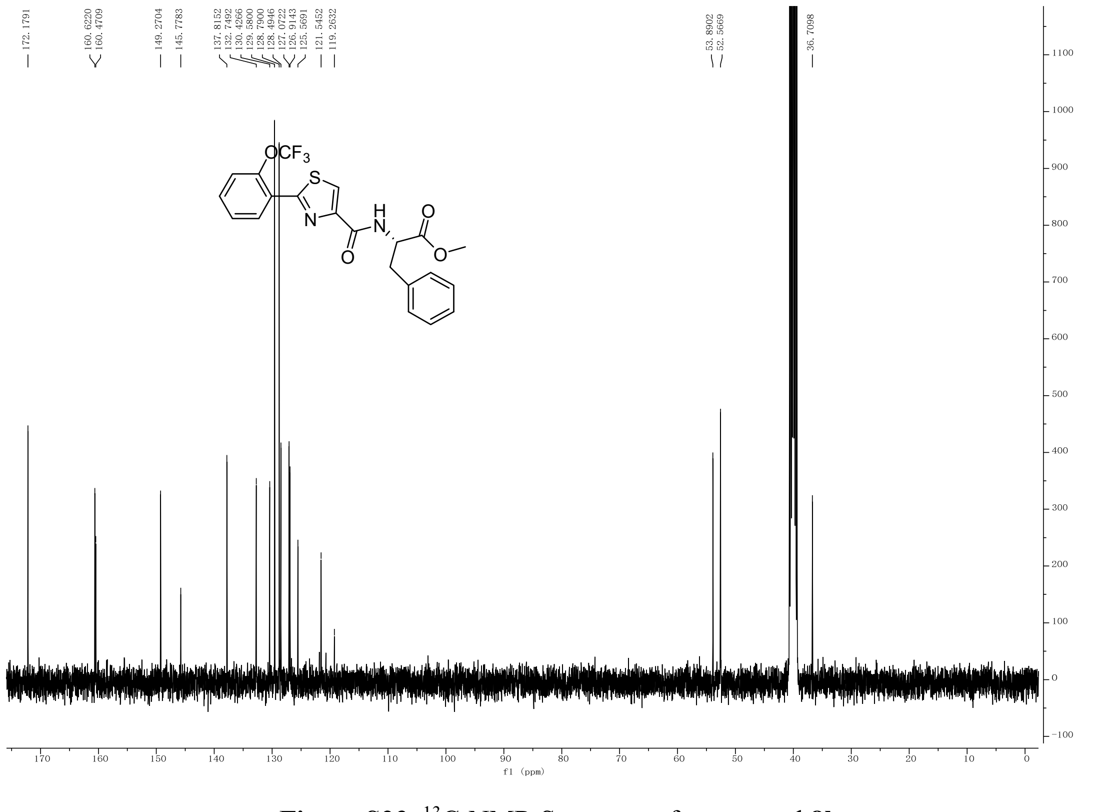

Figure S23. ^{13}C-NMR Spectrum of compound **8h**

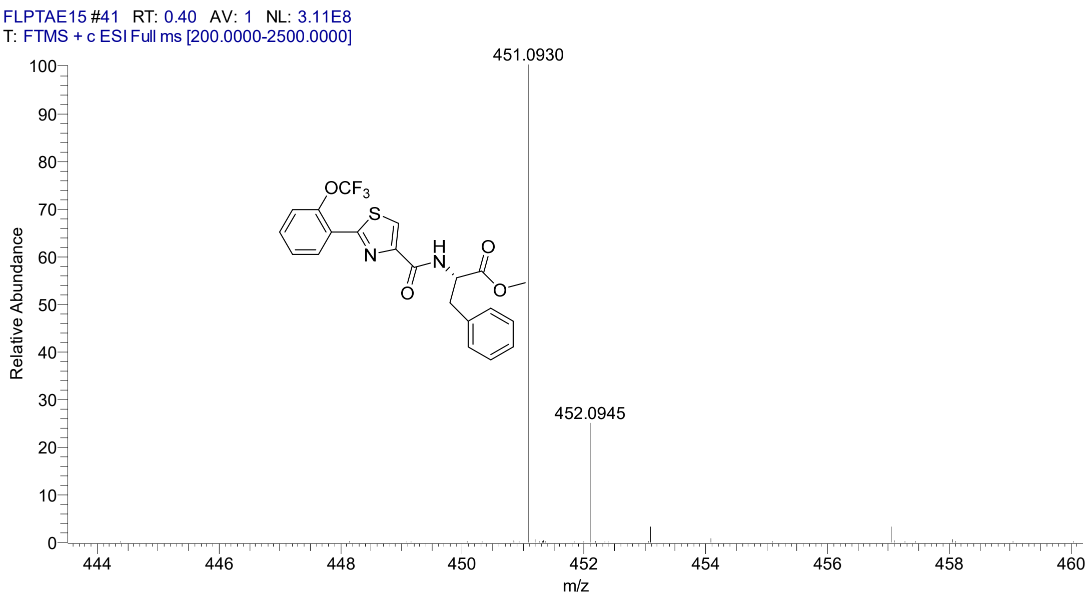

Figure S24. HRMS Spectrum of compound **8h**

8i: white solid; yield 75%, melting point 144.4~147.1 °C; ^{1}H NMR (400 MHz, DMSO-d_6) δ 9.34 – 9.22 (m, 1H), 8.62 (d, J = 8.0 Hz, 1H), 8.53 (dd, J = 2.0, 7.8 Hz, 1H), 8.46 (s, 1H), 7.74 – 7.58 (m, 3H), 7.10 – 7.04 (m, 2H), 6.72 – 6.62 (m, 2H), 4.75 – 4.64 (m, 1H), 3.66 (s, 3H), 3.11 (d, J = 7.2 Hz, 2H); ^{13}C NMR (101 MHz, DMSO-d_6) δ 172.27, 160.55, 160.47, 156.60, 149.28, 145.79, 132.75, 130.53, 130.39, 128.52, 127.51, 126.85, 125.55, 121.84, 121.55, 119.27, 115.63, 55.39, 54.13, 52.50, 36.04. HRMS(ESI) calcd for $C_{21}H_{18}F_3N_2O_5S^+$ [M+H]$^+$: 467.0883, found 467.0882.

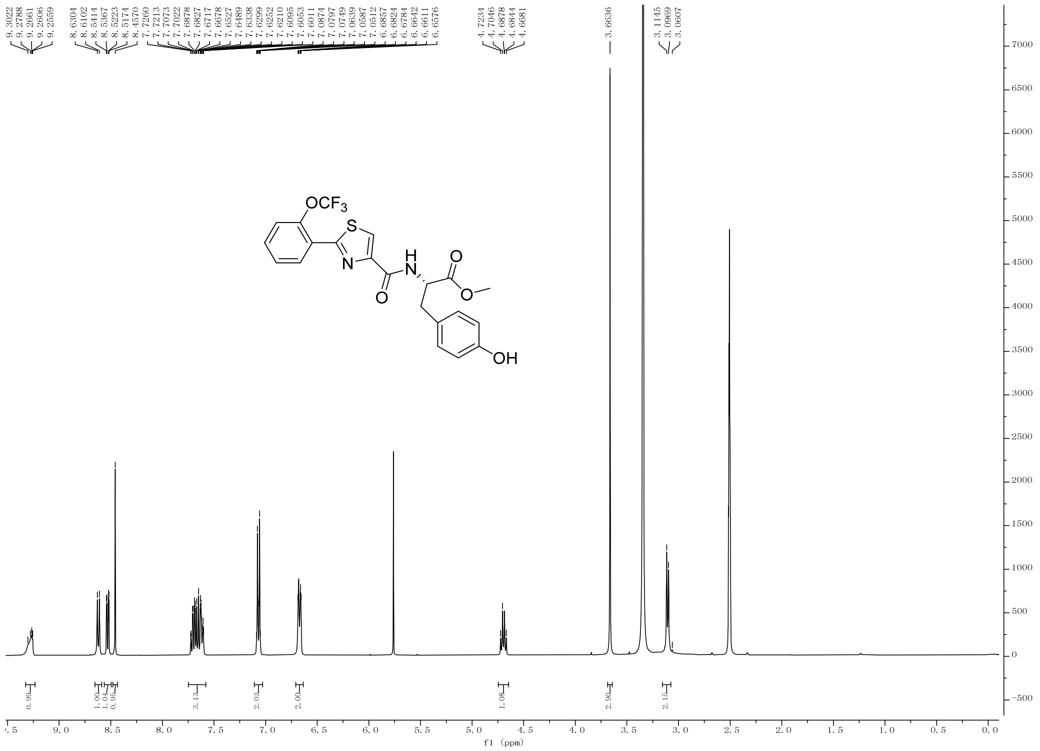

Figure S25. ^{1}H-NMR Spectrum of compound **8i**

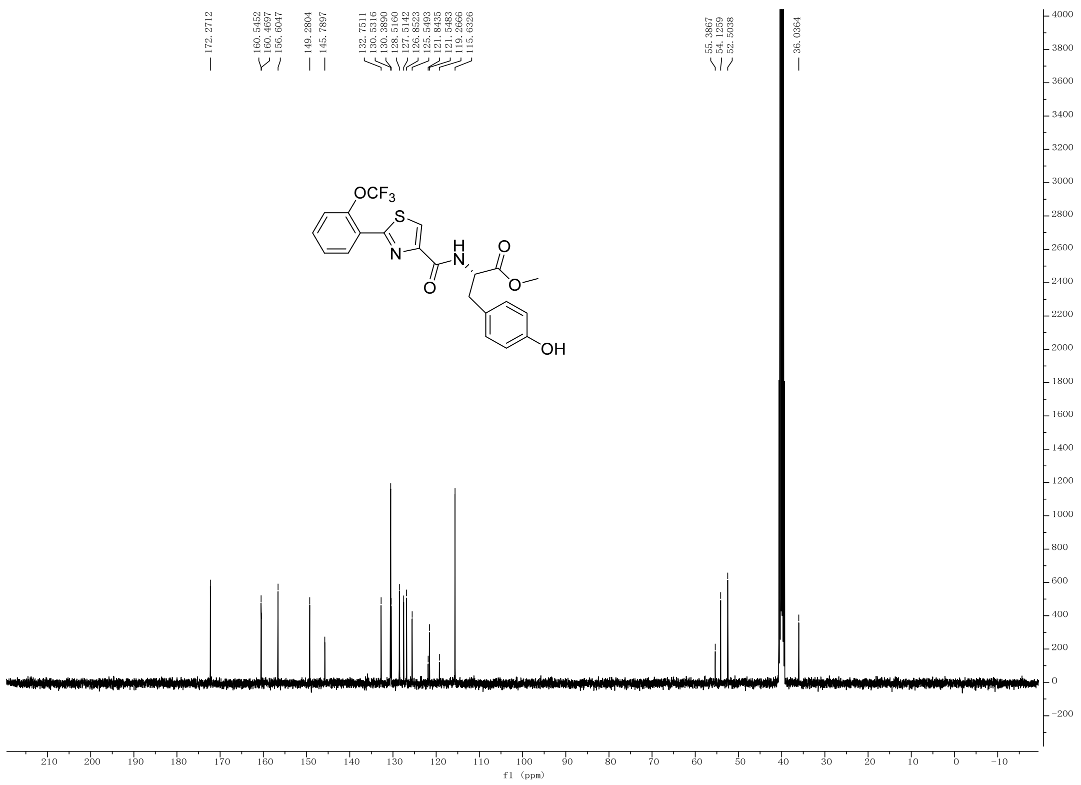

Figure S26. ^{13}C-NMR Spectrum of compound **8i**

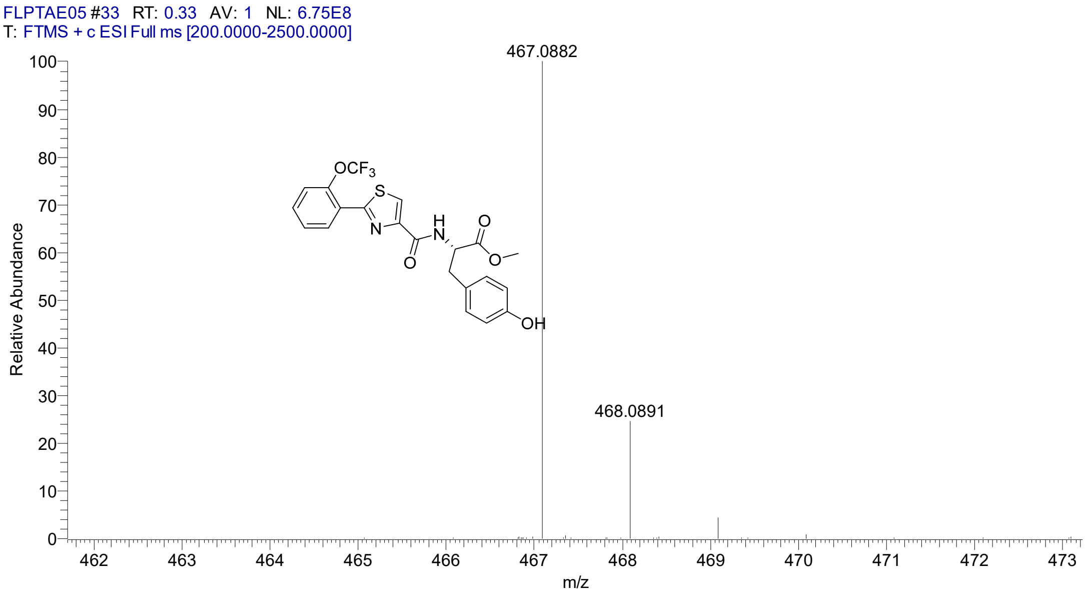

Figure S27. HRMS Spectrum of compound **8i**

8j: white solid; yield 83%, melting point 140.3 ~ 142.3 °C; ^{1}H NMR (400 MHz, DMSO-d_6) δ 10.95 (d, J = 2.4 Hz, 1H), 8.54 (d, J = 8.0 Hz, 1H), 8.47 (s, 1H), 8.37 (dd, J = 1.9, 7.8 Hz, 1H), 7.73 – 7.57 (m, 4H), 7.37 (d, J = 8.1 Hz, 1H), 7.24 (d, J = 2.4 Hz, 1H), 7.14 – 7.06 (m, 1H), 7.03 – 6.95 (m, 1H), 4.87 – 4.76 (m, 1H), 3.69 (s, 3H), 3.38

(d, J = 6.5 Hz, 2H); ^{13}C NMR (101 MHz, DMSO-d_6) δ 172.43, 160.56, 160.43, 149.21, 145.77, 136.61, 132.73, 130.32, 128.50, 127.70, 126.88, 125.45, 124.29, 121.83, 121.54, 119.25, 119.03, 118.56, 111.99, 109.68, 53.58, 52.57, 27.02. HRMS(ESI) calcd for $C_{23}H_{19}F_3N_3O_4S^+$ [M+H]$^+$: 490.1043, found 490.1042.

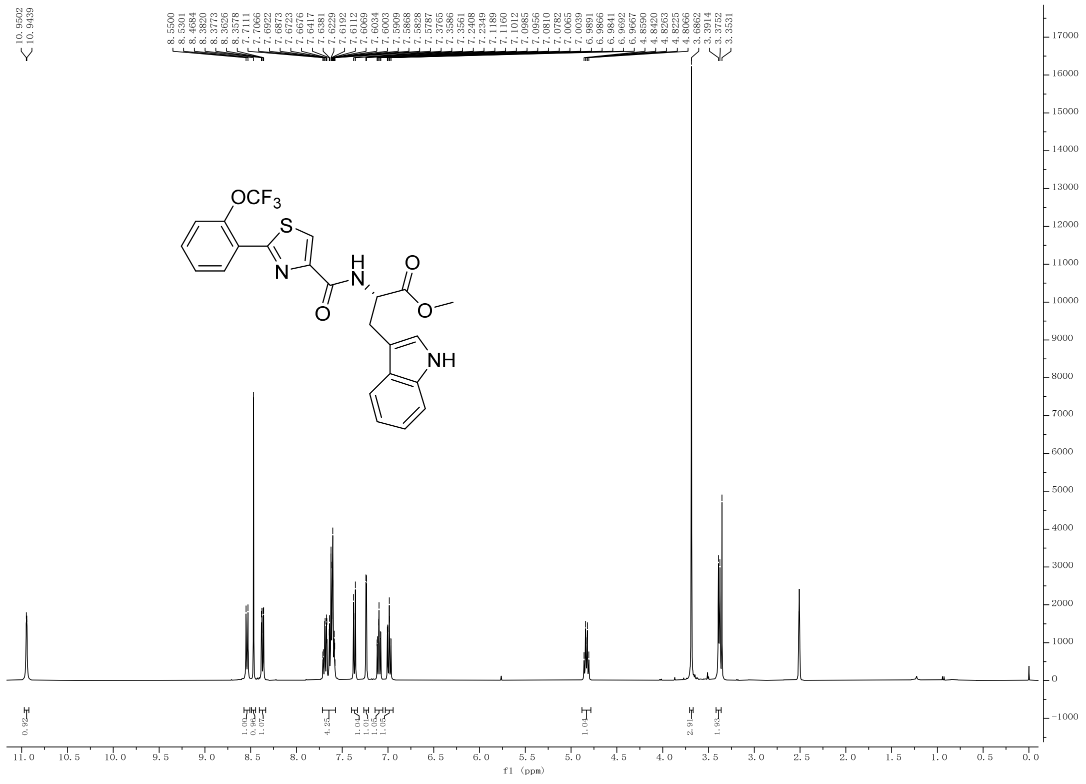

Figure S28. ^1H-NMR Spectrum of compound **8j**

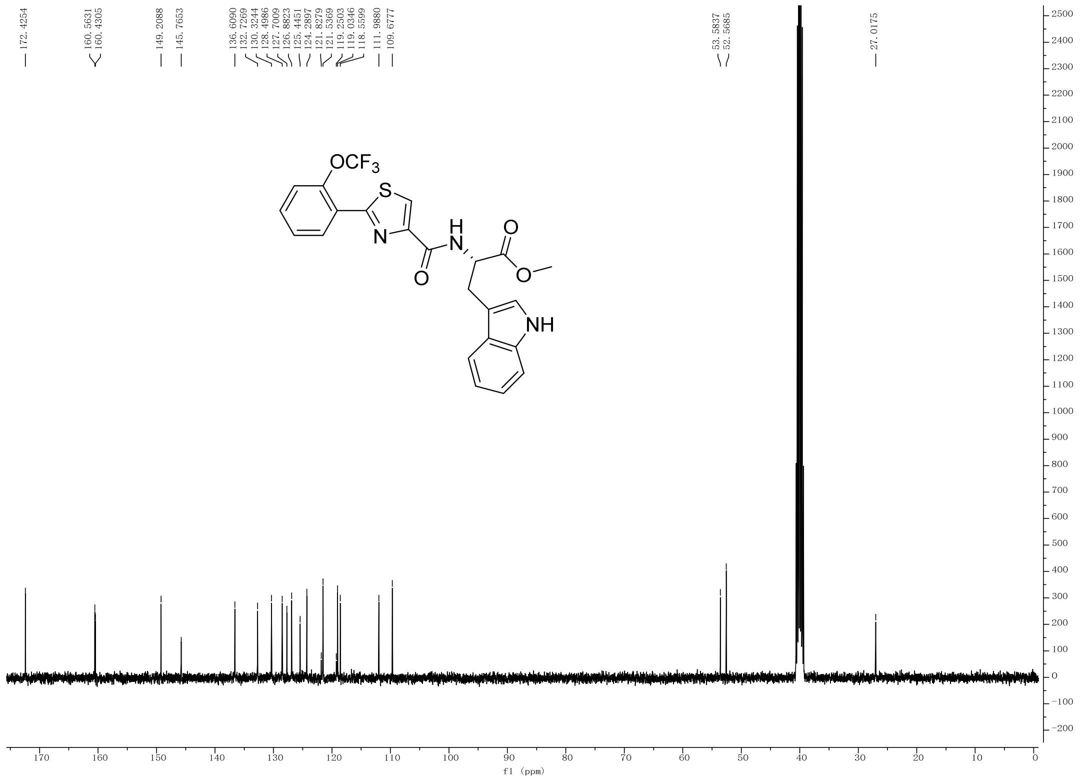

Figure S29. ^{13}C-NMR Spectrum of compound **8j**

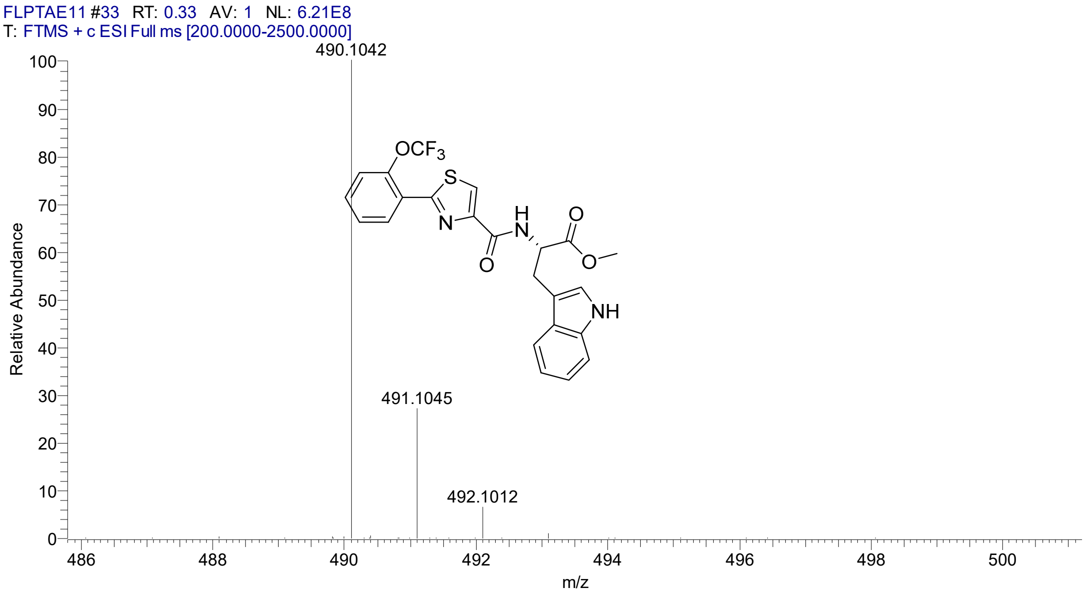

Figure S30. HRMS Spectrum of compound **8j**

8k: white solid; yield 72%, melting point 110.3~113.8 °C; ^{1}H NMR (400 MHz, DMSO-d_6) δ 8.88 (d, J = 7.6 Hz, 1H), 8.61 (dd, J = 2.0, 8.0 Hz, 1H), 8.50 (s, 1H), 7.74 – 7.66 (m, 1H), 7.65 – 7.58 (m, 2H), 4.59 (t, J = 7.4 Hz, 1H), 3.67 (s, 3H), 1.46 (d, J = 7.2 Hz, 3H); ^{13}C NMR (101 MHz, DMSO-d_6) δ 173.21, 160.55, 160.42, 149.48, 145.78,

132.71, 130.57, 128.45, 126.88, 125.63, 121.85, 121.53, 52.51, 48.17, 17.41. HRMS(ESI) calcd for $C_{15}H_{14}F_3N_2O_4S^+$ [M+H]$^+$: 375.0621, found 375.0619.

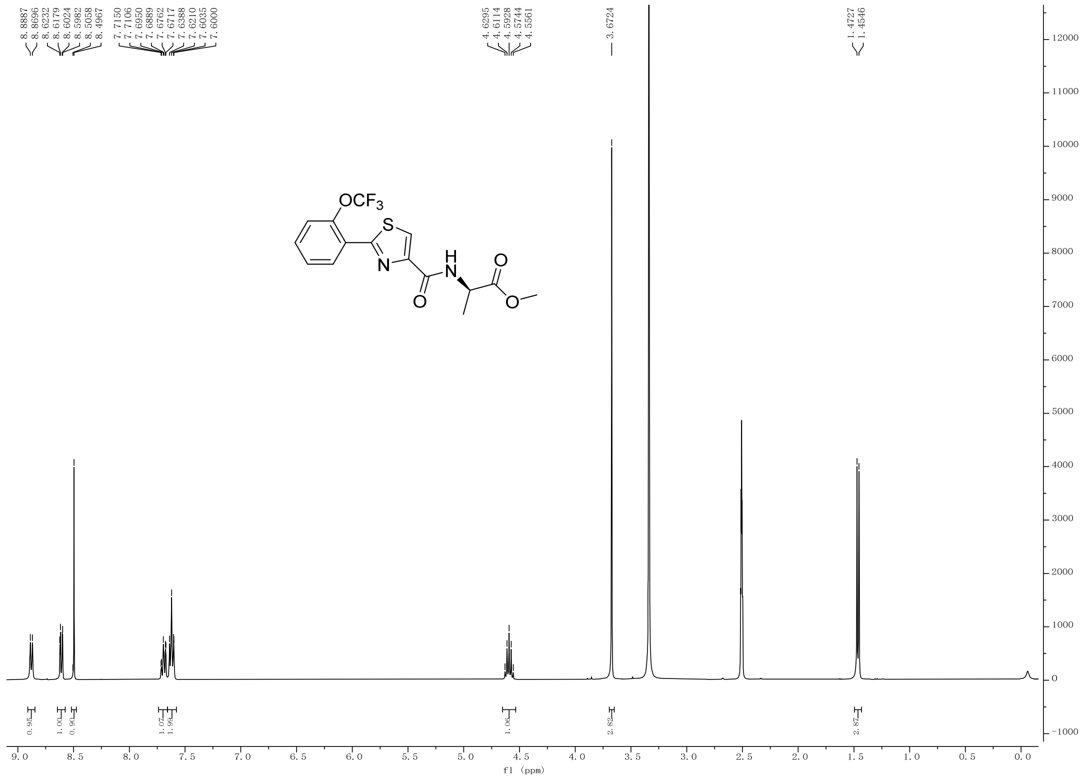

Figure S31. ^{1}H-NMR Spectrum of compound **8k**

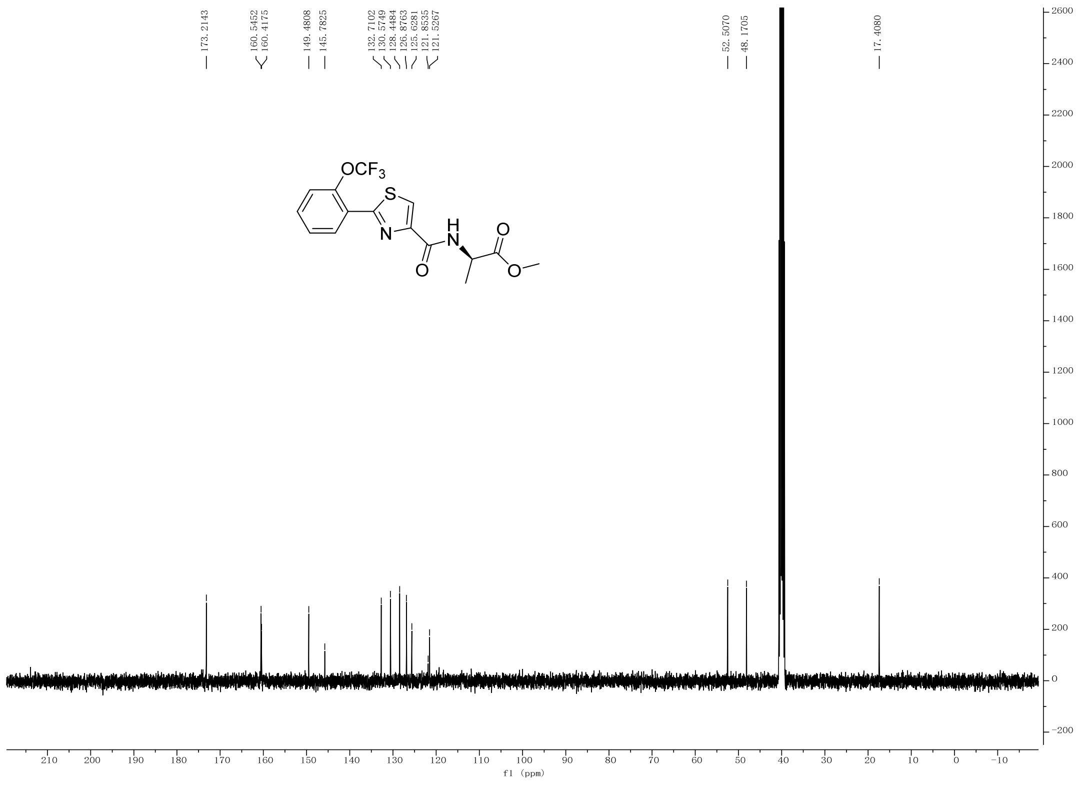

Figure S32. ^{13}C-NMR Spectrum of compound **8k**

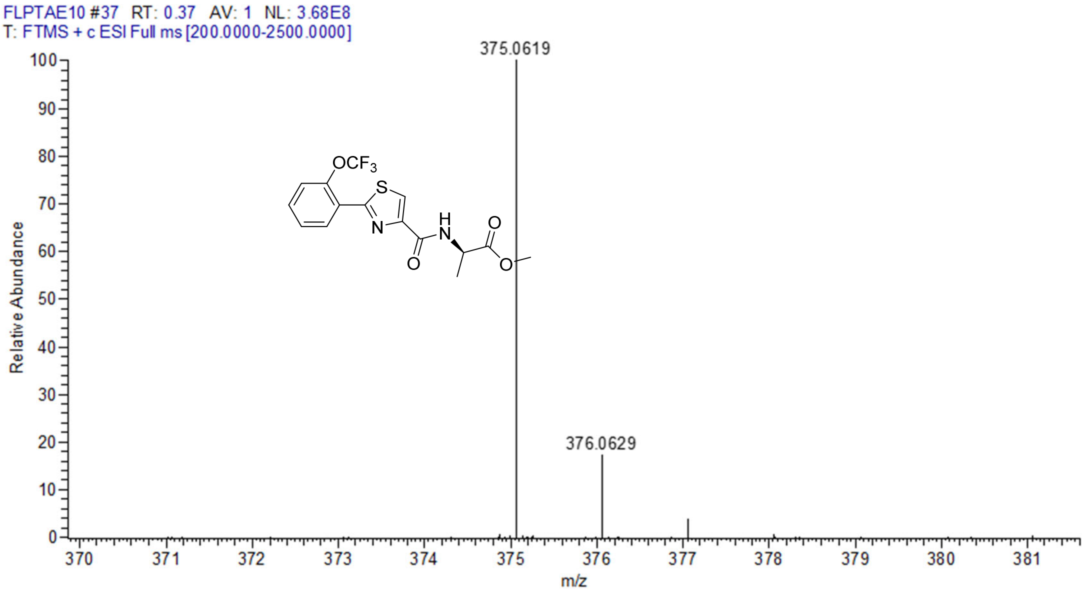

Figure S33. HRMS Spectrum of compound **8k**

8l: white solid; yield 79%, melting point 97.6~99.7 °C; ^{1}H NMR (400 MHz, DMSO-d_6) δ 8.84 (d, J = 7.5 Hz, 1H), 8.62 (dd, J = 1.6, 7.6 Hz, 1H), 8.52 (s, 1H), 7.74 – 7.66 (m, 1H), 7.66 – 7.57 (m, 2H), 4.58 (t, J = 7.6 Hz, 1H), 4.21 – 4.08 (m, 2H), 1.48 (d, J = 7.2 Hz, 3H), 1.22 (t, J = 7.2 Hz, 3H); ^{13}C NMR (101 MHz, DMSO-d_6) δ 172.73, 160.56, 160.41, 149.52, 145.77, 132.65, 130.55, 128.40, 126.79, 125.61, 121.85, 121.46, 61.11, 48.32, 17.39, 14.51. HRMS(ESI) calcd for $C_{16}H_{16}F_3N_2O_4S^+$ [M+H]$^+$: 389.0777, found 389.0777.

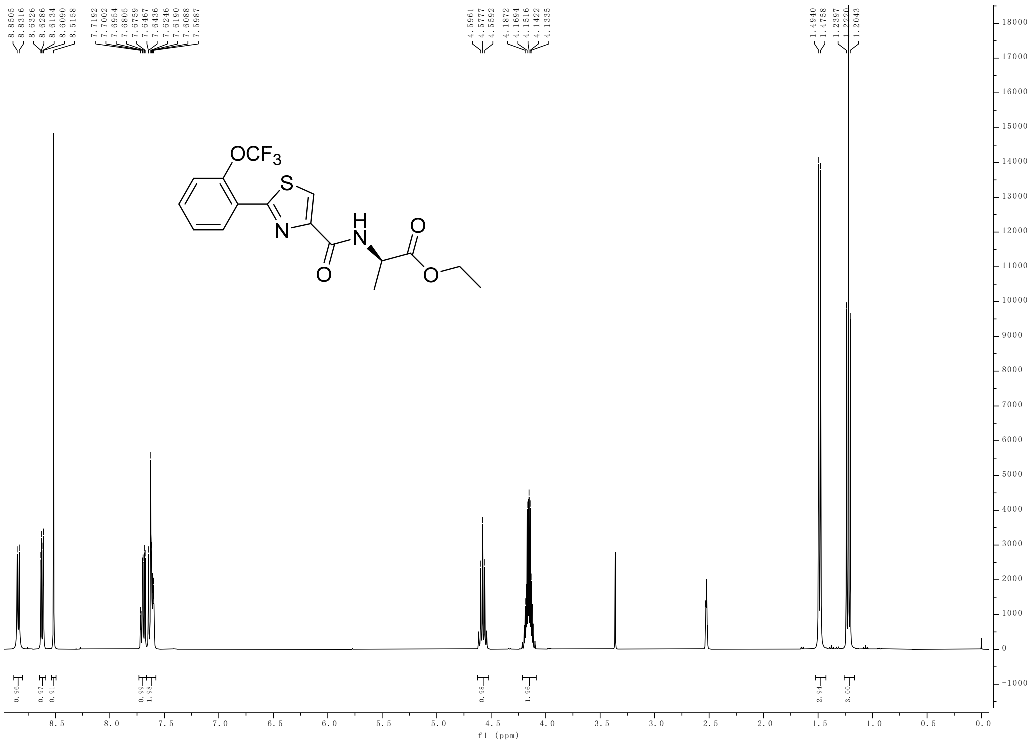

Figure S34. ^{1}H-NMR Spectrum of compound **8l**

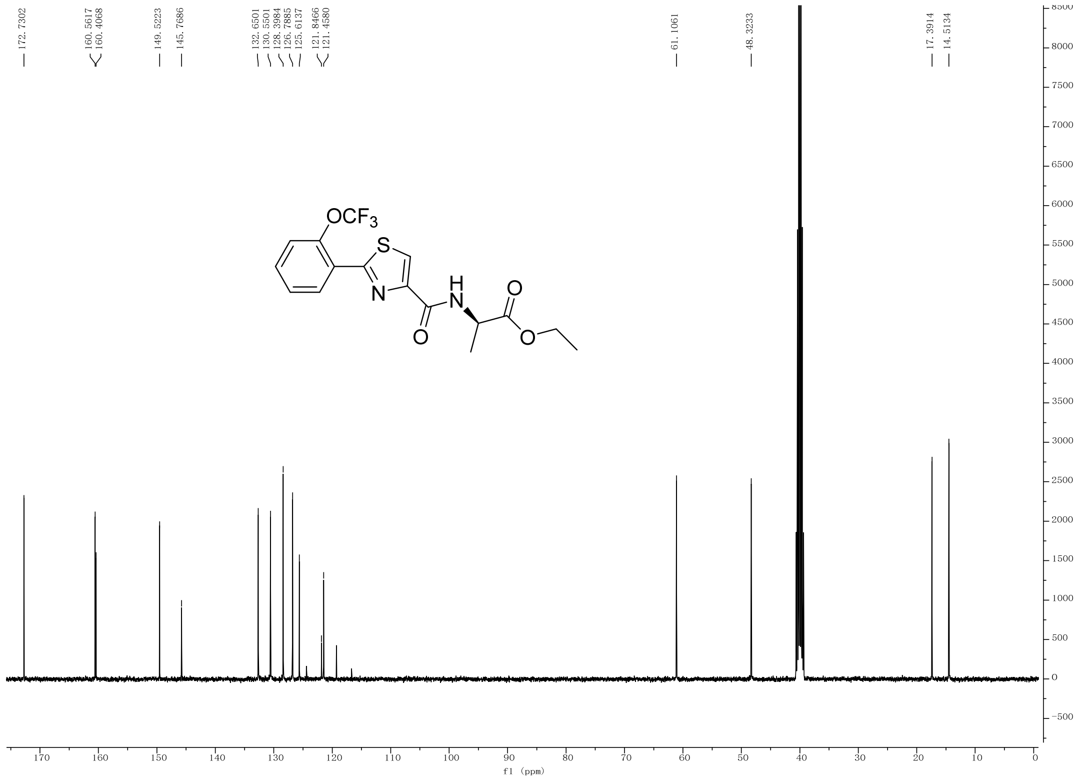

Figure S35. ^{13}C-NMR Spectrum of compound **8l**

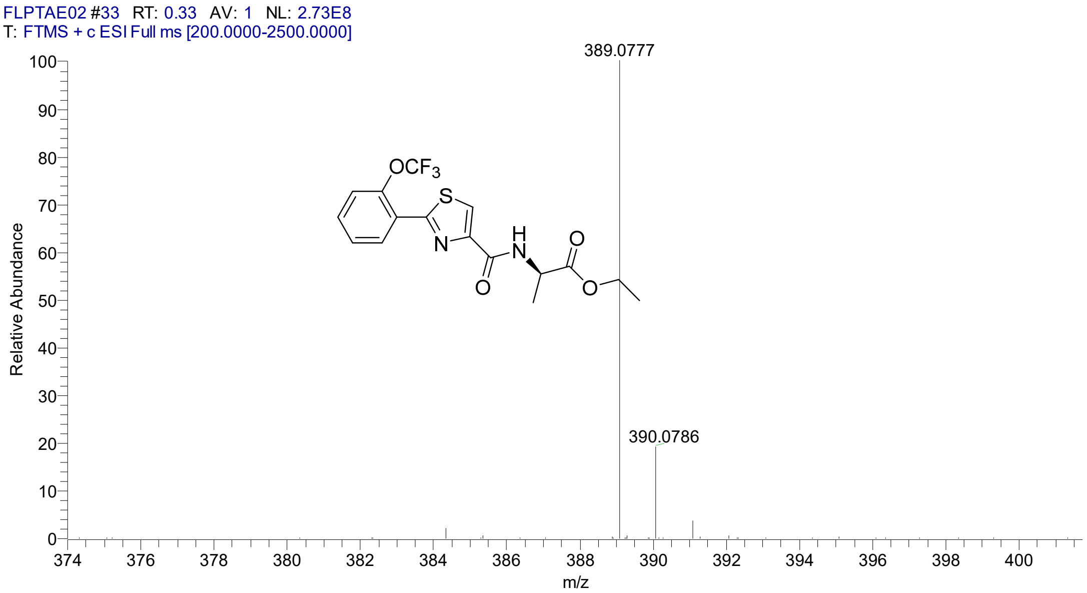

Figure S36. HRMS Spectrum of compound **8l**

8m: white solid; yield 72%, melting point 81.5~83.2°C; ^{1}H NMR (400 MHz, DMSO-d_6) δ 8.91 (d, J = 8.0 Hz, 1H), 8.62 (dd, J = 2.0, 7.6 Hz, 1H), 8.52 (s, 1H), 7.75 – 7.57 (m, 3H), 4.77 – 4.66 (m, 1H), 3.70 (s, 3H), 2.67 – 2.50 (m, 2H), 2.18 (q, J = 7.6 Hz, 2H), 2.08 (s, 3H); ^{13}C NMR (101 MHz, DMSO-d_6) δ 172.53, 161.02, 160.44,

149.41, 145.78, 132.69, 130.57, 128.42, 126.99, 125.62, 121.85, 121.49, 52.59, 51.56, 30.55, 30.36, 15.03. HRMS(ESI) calcd for $C_{17}H_{18}F_3N_2O_4S_2^+$ [M+H]$^+$: 435.0655, found 435.0652.

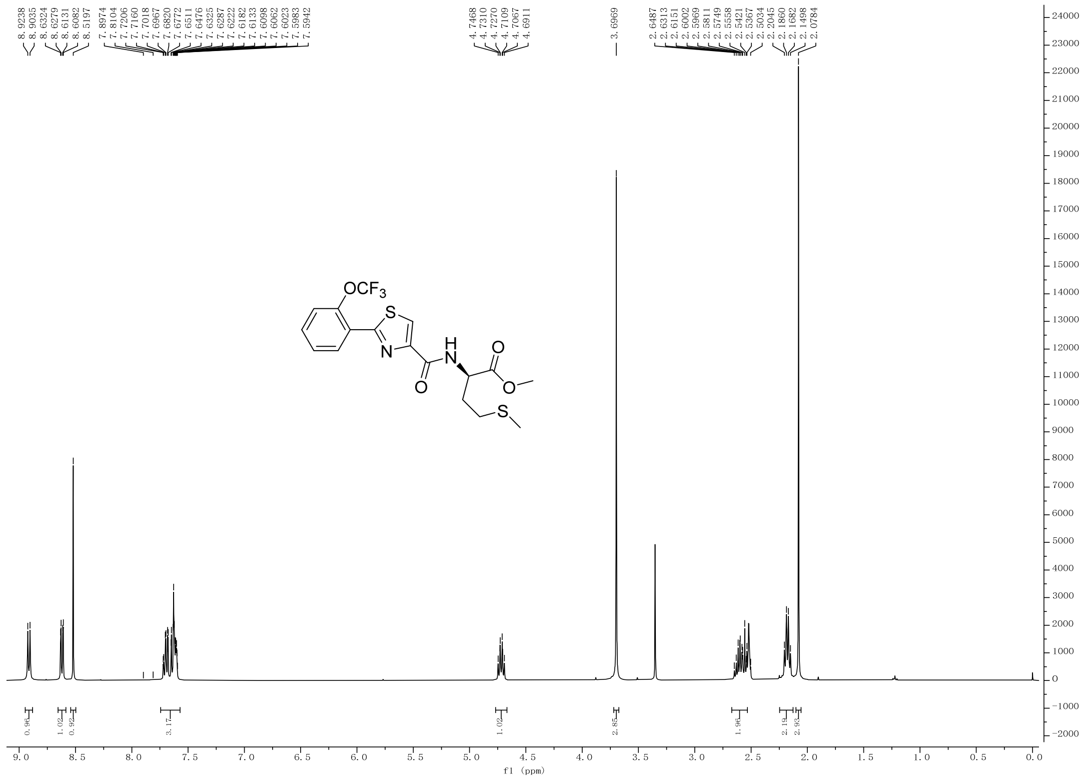

Figure S37. ^{1}H-NMR Spectrum of compound **8m**

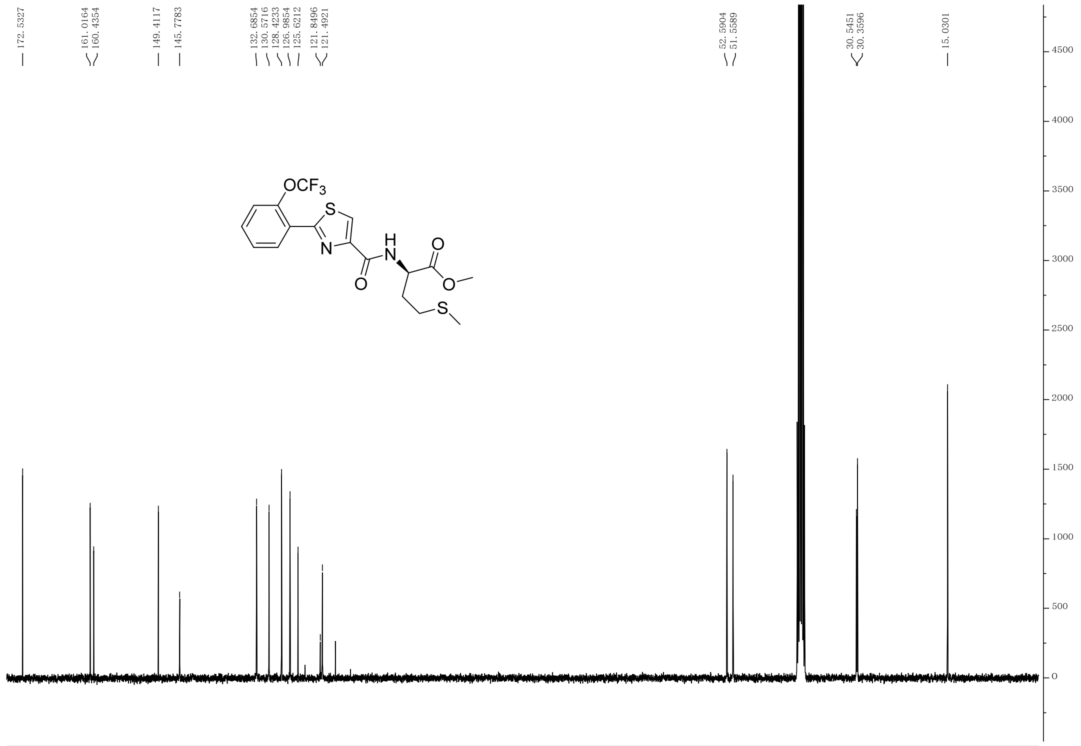

Figure S38. ^{13}C-NMR Spectrum of compound **8m**

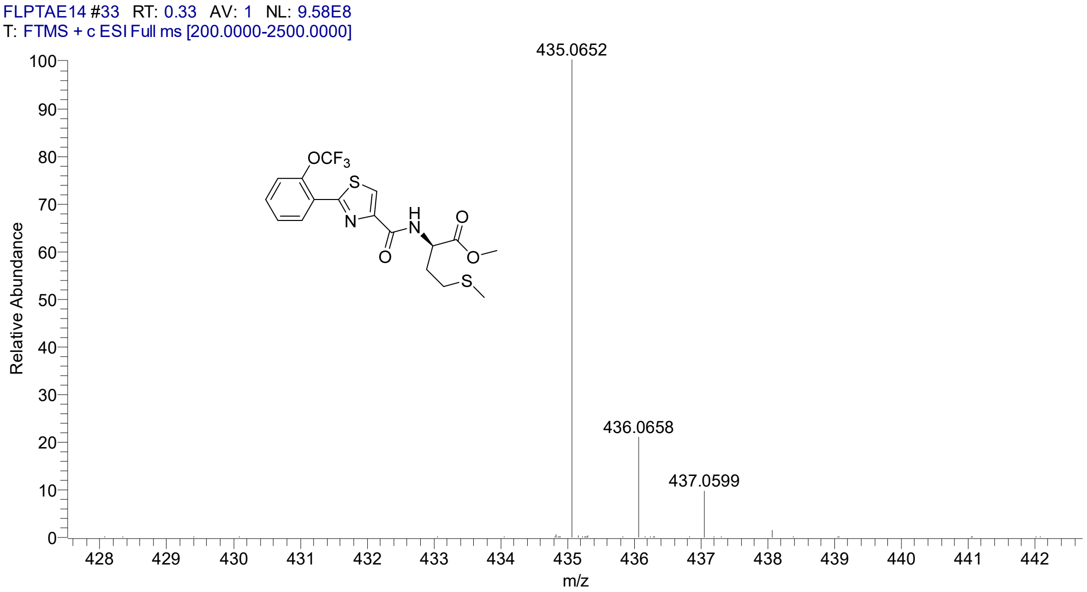

Figure S39. HRMS Spectrum of compound **8m**

8n: white solid; yield 81%, melting point 93.2~95.1 °C; ^{1}H NMR (400 MHz, DMSO-d_6) δ 8.58 – 8.48 (m, 2H), 8.41 (d, J = 8.4 Hz, 1H), 7.74 – 7.58 (m, 3H), 4.42 (dd, J = 6.8, 8.4 Hz, 1H), 3.70 (s, 3H), 2.27 (m, 1H), 0.97 (dd, J = 5.6, 6.8 Hz, 6H); ^{13}C NMR (101 MHz, DMSO-d_6) δ 172.23, 160.81, 149.36, 145.70, 132.75, 130.68, 128.60, 127.01, 125.60, 121.86, 121.71, 119.29, 58.06, 52.40, 30.35, 19.54, 18.96. HRMS(ESI) calcd for $C_{17}H_{18}F_3N_2O_4S^+$ [M+H]$^+$: 403.0934, found 403.0930.

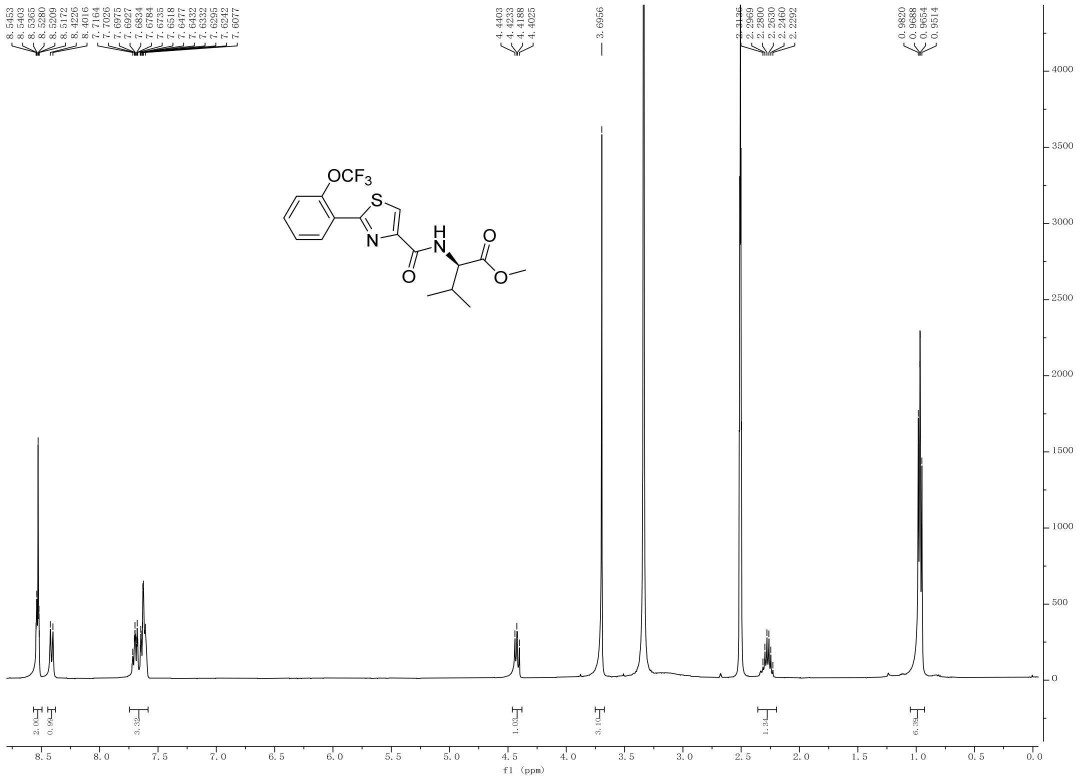

Figure S40. ^{1}H-NMR Spectrum of compound **8n**

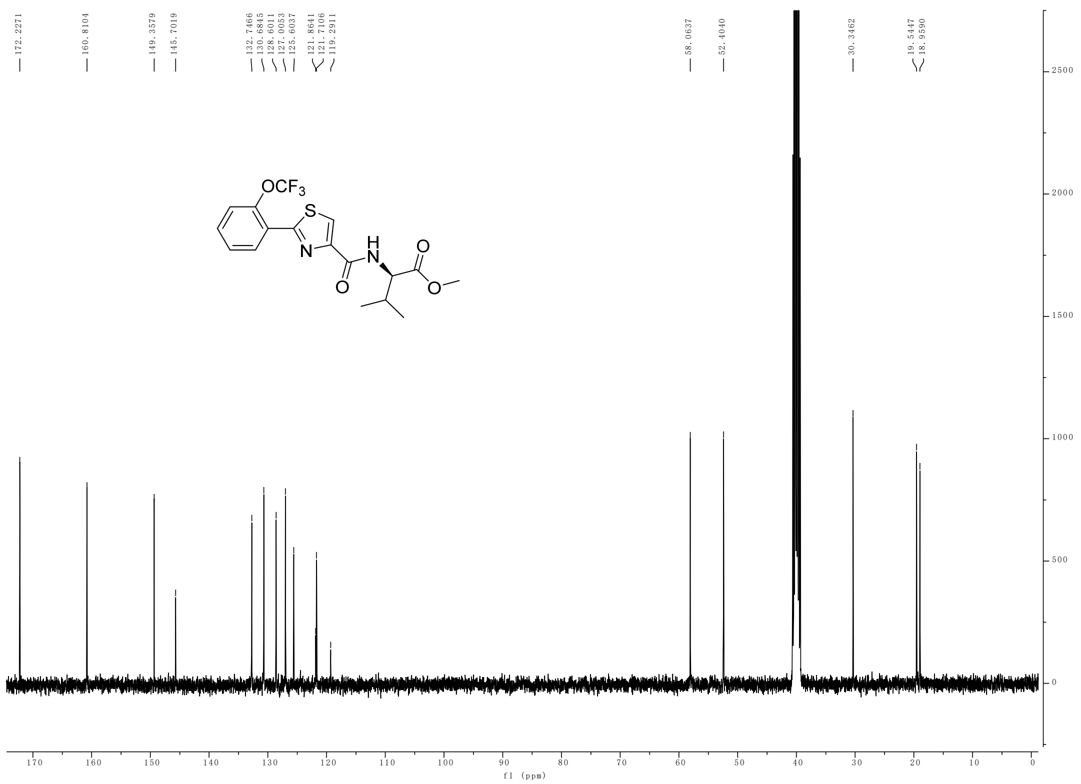

Figure S41. ^{13}C-NMR Spectrum of compound **8n**

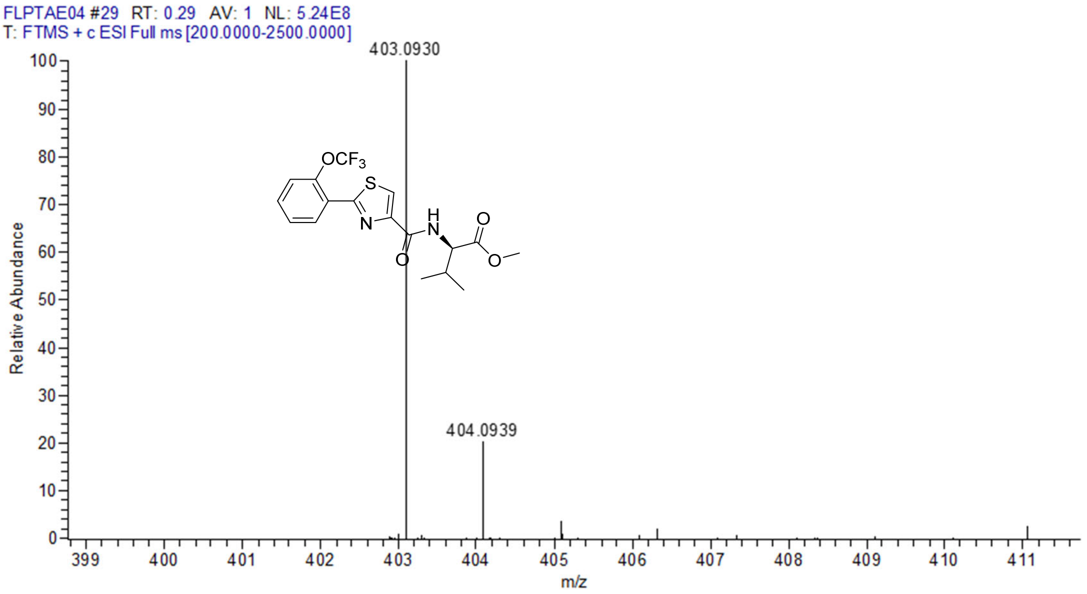

Figure S42. HRMS Spectrum of compound **8n**

8o: white solid; yield 77%, melting point 105.1~107.5 °C; ^{1}H NMR (400 MHz, DMSO-d_6) δ 8.79 (d, J = 8.4 Hz, 1H), 8.61 (dd, J = 2.0, 7.6 Hz, 1H), 8.50 (s, 1H), 7.73 – 7.56 (m, 3H), 4.65 – 4.57 (m, 1H), 3.67 (s, 3H), 1.96 – 1.81 (m, 1H), 1.73 – 1.60 (m, 2H), 0.91 (dd, J = 6.0, 10.4 Hz, 6H); ^{13}C NMR (101 MHz, DMSO-d_6) δ 173.16, 160.88,

160.47, 149.45, 145.76, 132.69, 130.63, 128.45, 126.93, 125.63, 121.85, 121.52, 119.28, 52.48, 50.81, 24.86, 23.34. HRMS(ESI) calcd for $C_{18}H_{20}F_3N_2O_4S^+$ $[M+H]^+$: 417.1090, found 417.1090.

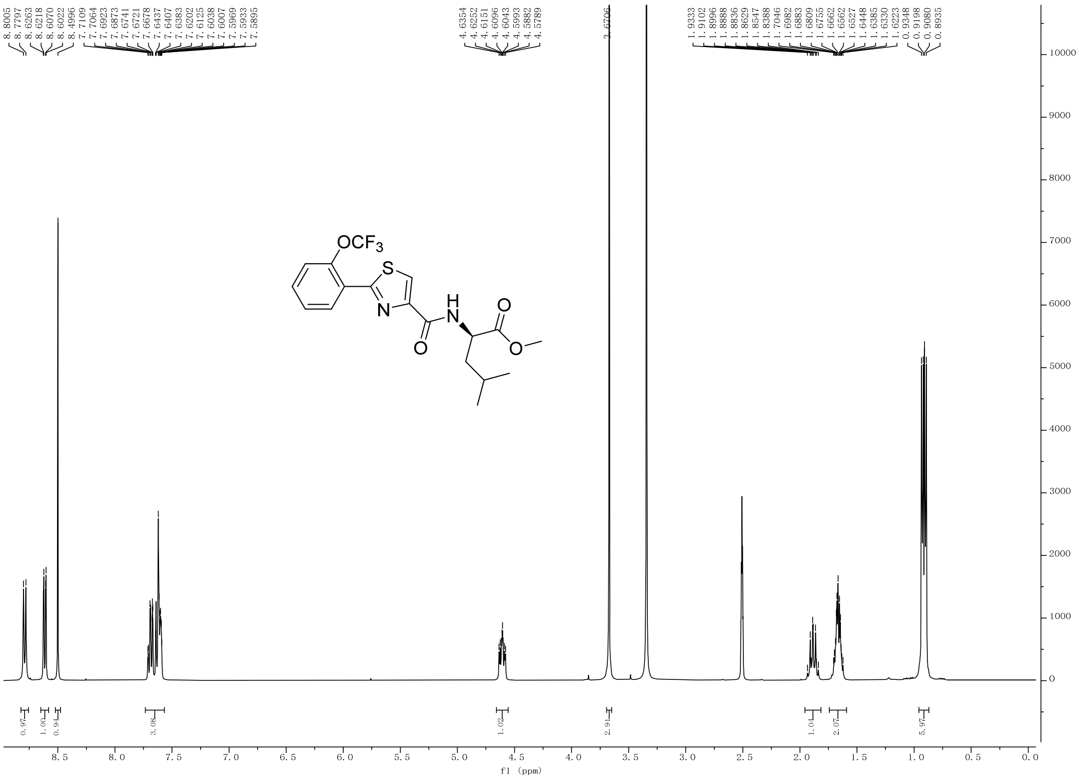

Figure S43. ^{1}H-NMR Spectrum of compound **8o**

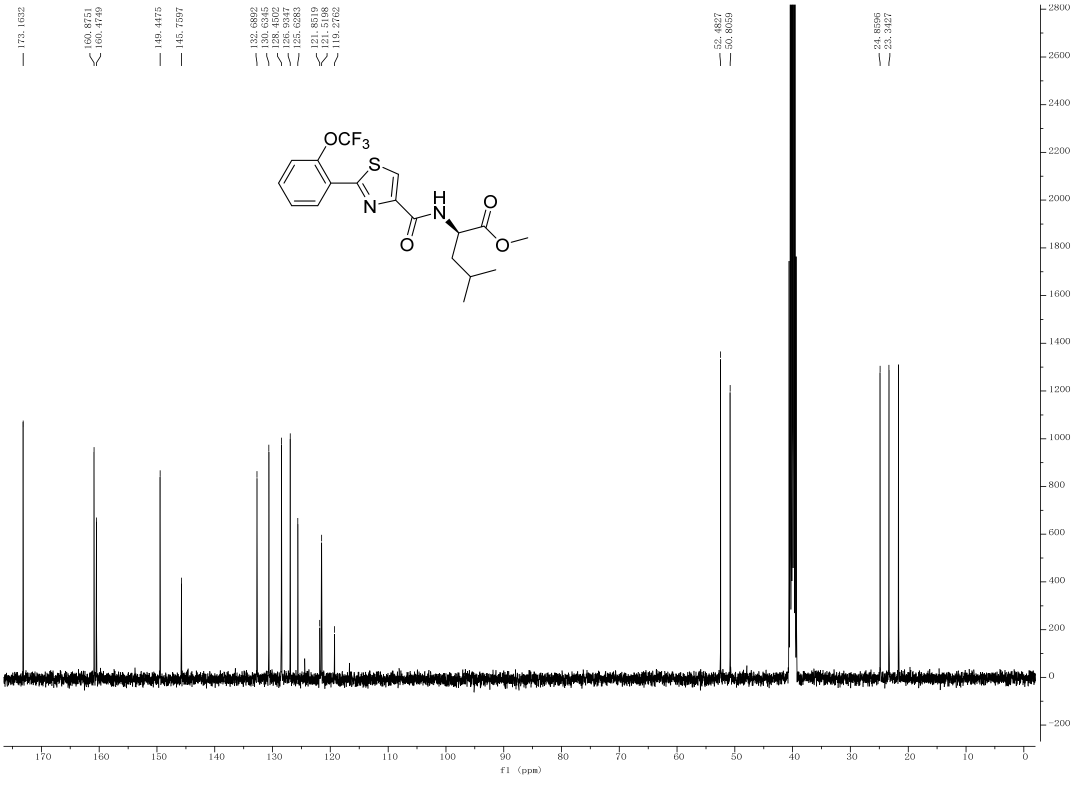

Figure S44. ^{13}C-NMR Spectrum of compound **8o**

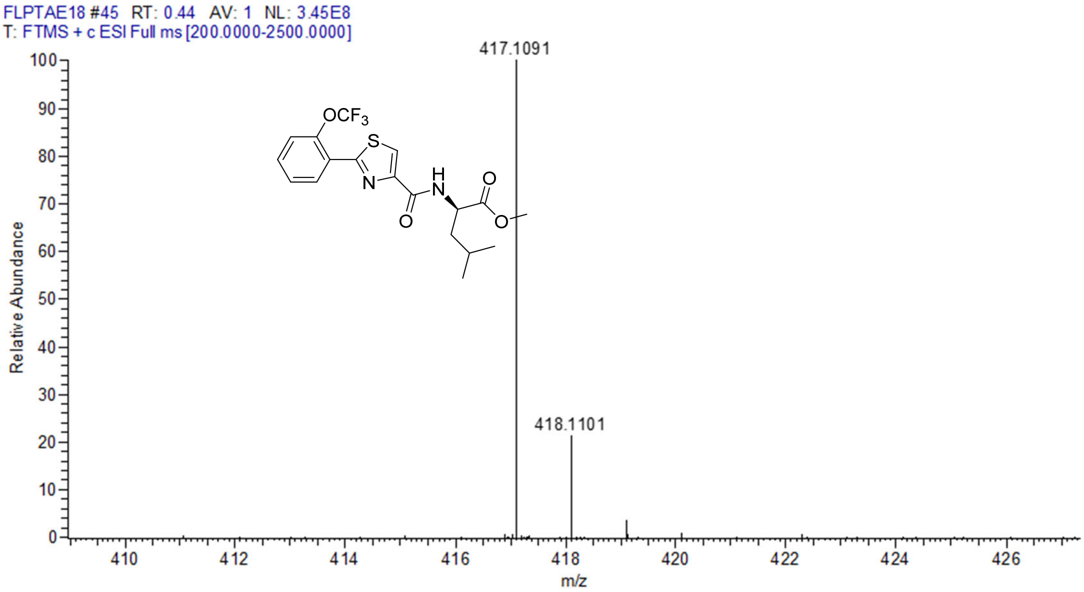

Figure S45. HRMS Spectrum of compound **8o**

8p: white solid; yield 80%, melting point 111.4~113.9 °C; ^{1}H NMR (400 MHz, DMSO-d_6) δ 8.76 (d, J = 8.0 Hz, 1H), 8.54 (dd, J = 2.0, 7.6 Hz, 1H), 8.45 (s, 1H), 7.73 – 7.59 (m, 3H), 7.34 – 7.24 (m, 4H), 7.21 (dd, J = 2.4, 6.0 Hz, 1H), 4.84 – 4.75 (m, 1H), 3.67 (s, 3H), 3.28 – 3.19 (m, 2H); ^{13}C NMR (101 MHz, DMSO-d_6) δ 173.21, 160.54, 160.42, 149.49, 145.79, 145.78, 132.69, 130.57, 128.43, 126.85, 125.62, 124.43, 121.85, 121.49, 119.28, 116.70, 52.49, 48.17, 17.41. HRMS(ESI) calcd for $C_{21}H_{18}F_3N_2O_4S^+$ [M+H]$^+$: 451.0934, found 451.0931.

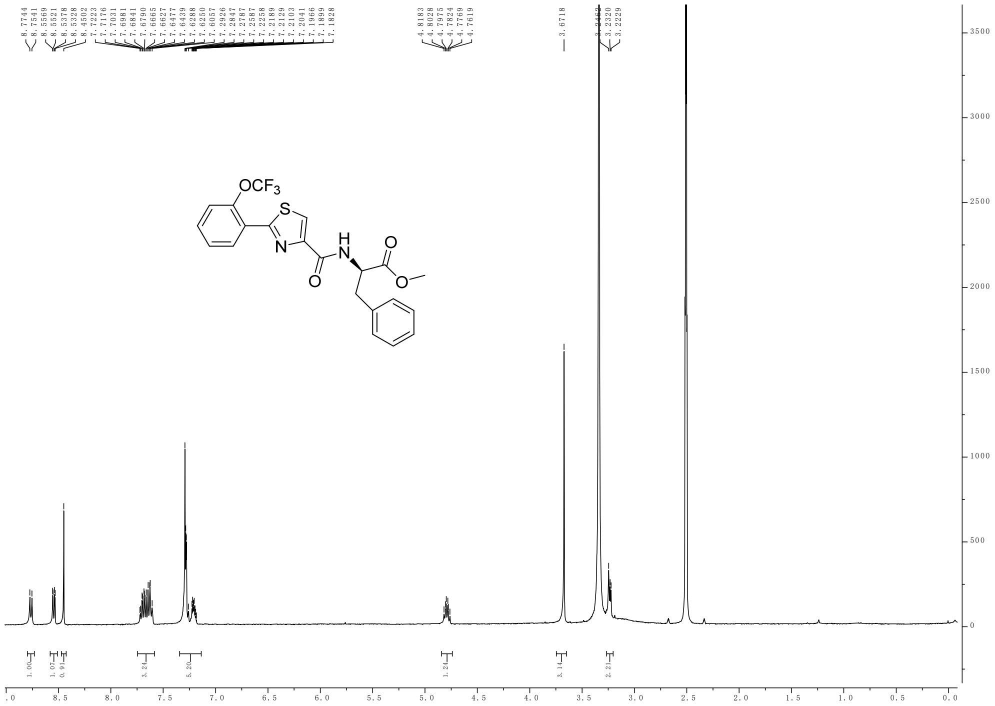

Figure S46. ^{1}H-NMR Spectrum of compound **8p**

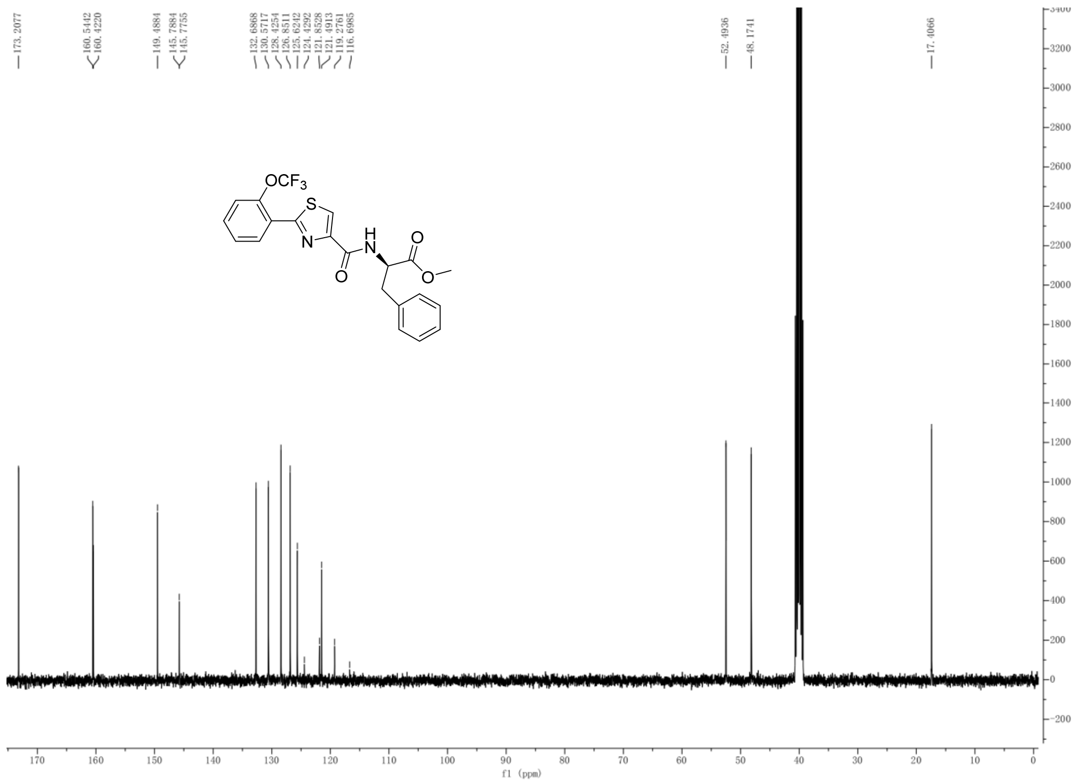

Figure S47. ^{13}C-NMR Spectrum of compound **8p**

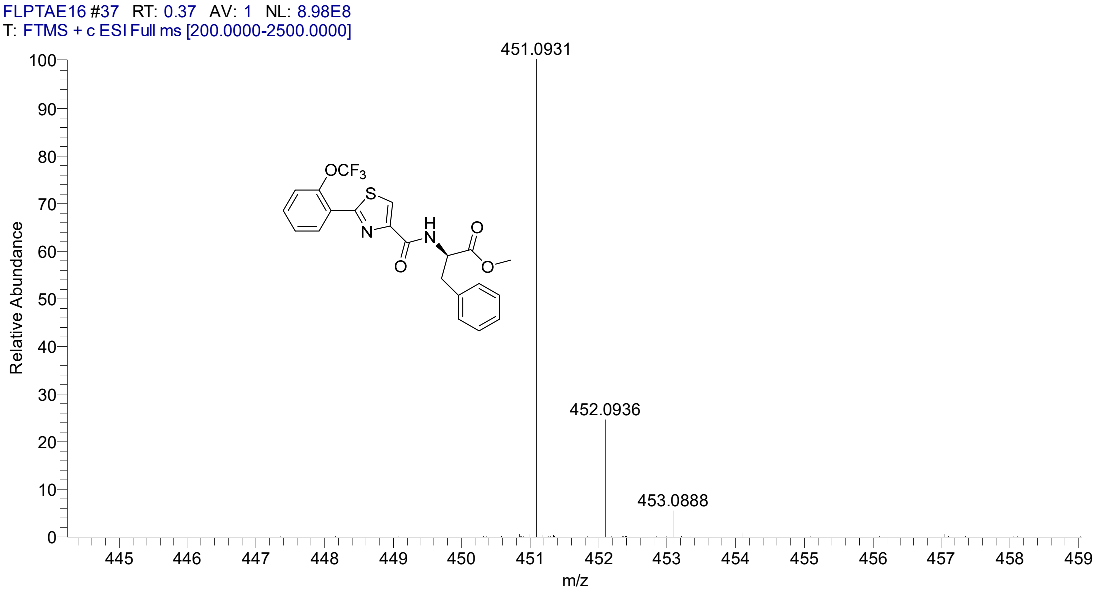

Figure S48. HRMS Spectrum of compound **8p**

8q: white solid; yield 78%, melting point 146.3~148.1 °C; ^{1}H NMR (400 MHz, DMSO-d_6) δ 9.24 (s, 1H), 8.62 (d, J = 8.0 Hz, 1H), 8.53 (dd, J = 2.0, 7.6 Hz, 1H), 8.45 (s, 1H), 7.74 – 7.68 (m, 1H), 7.67 – 7.59 (m, 2H), 7.07 (d, J = 8.4 Hz, 2H), 6.66 (d, J =

8.4 Hz, 2H), 4.69 (q, J = 7.6 Hz, 1H), 3.66 (s, 3H), 3.11 (d, J = 7.2 Hz, 2H); ^{13}C NMR (101 MHz, DMSO-d_6) δ 172.28, 160.55, 160.47, 156.56, 149.28, 145.79, 132.75, 130.55, 130.39, 128.51, 127.56, 126.86, 125.55, 121.84, 121.56, 119.27, 115.62, 55.38, 54.13, 52.51, 36.02. HRMS(ESI) calcd for $C_{21}H_{18}F_3N_2O_5S^+$ [M+H]$^+$: 467.0883, found 467.0875.

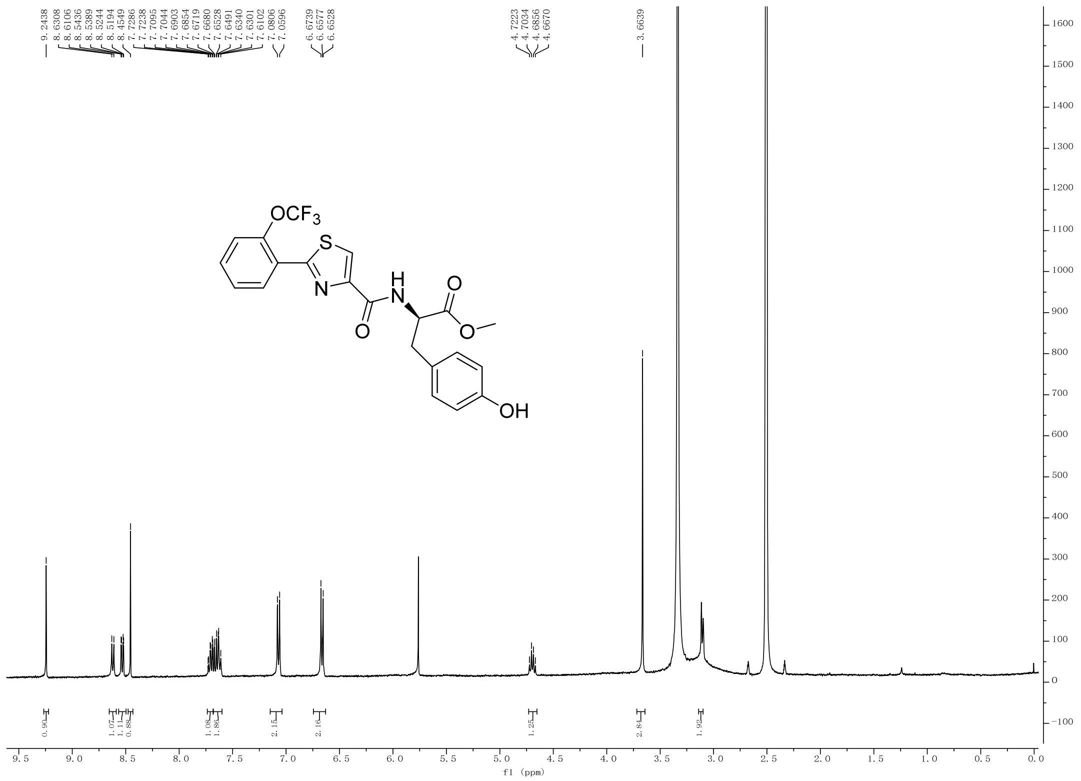

Figure S49. ^{1}H-NMR Spectrum of compound **8q**

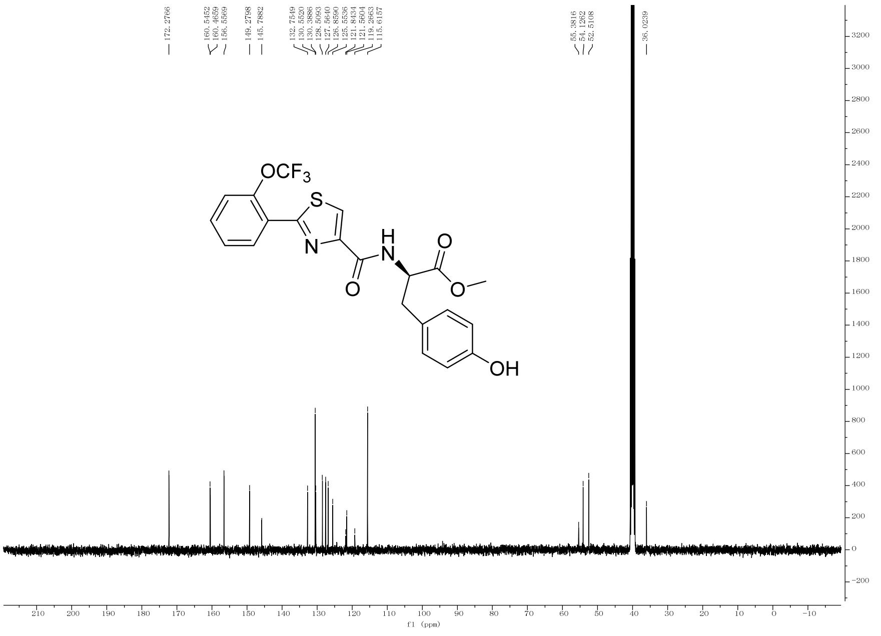

Figure S50. ^{13}C-NMR Spectrum of compound **8q**

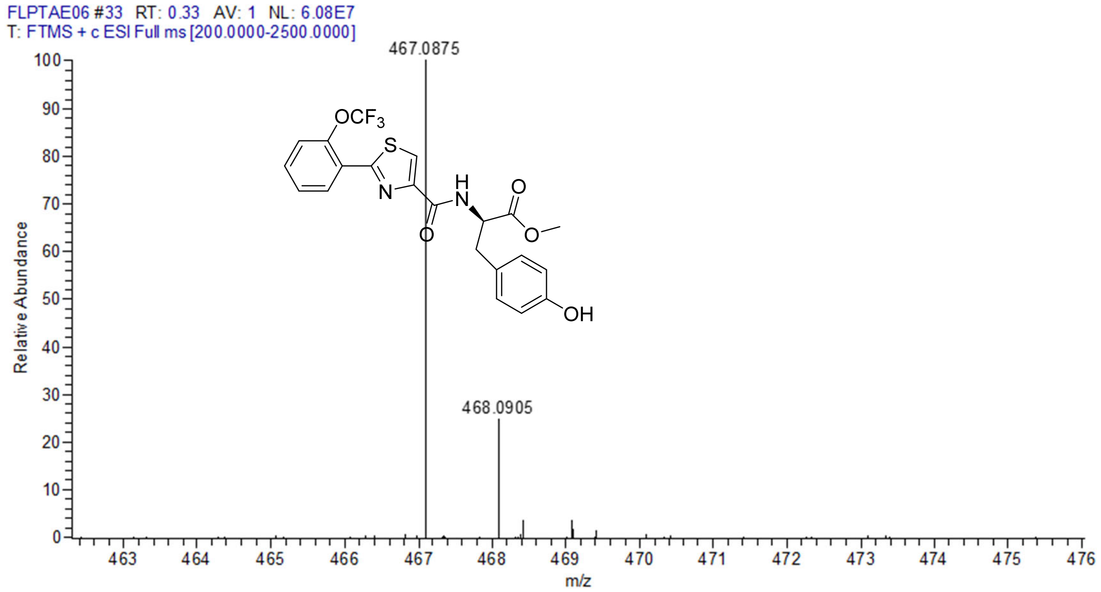

Figure S51. HRMS Spectrum of compound **8q**

8r: white solid; yield 79%, melting point 91~92.8 °C; ^{1}H NMR (400 MHz, DMSO-d_6) δ 10.94 (s, 1H), 8.54 (d, J = 8.0 Hz, 1H), 8.46 (s, 1H), 8.37 (dd, J = 1.6, 7.6 Hz, 1H), 7.73 – 7.66 (m, 1H), 7.65 – 7.55 (m, 3H), 7.36 (d, J = 8.0 Hz, 1H), 7.23 (d, J = 2.4 Hz, 1H), 7.09 (t, J = 7.6 Hz, 1H), 6.98 (t, J = 7.6 Hz, 1H), 4.82 (q, J = 6.8 Hz, 1H), 3.68 (s, 3H), 3.37 (d, J = 6.4 Hz, 2H); ^{13}C NMR (101 MHz, DMSO-d_6) δ 172.43, 160.56, 160.43, 149.21, 145.76, 136.61, 132.71, 130.32, 128.49, 127.70, 126.87, 125.44, 124.29, 121.83, 121.49, 119.25, 119.04, 118.56, 111.99, 109.68, 53.59, 52.57, 27.02. HRMS(ESI) calcd for $C_{23}H_{19}F_3N_3O_4S^+$ [M+H]$^+$: 490.1043, found 490.1041.

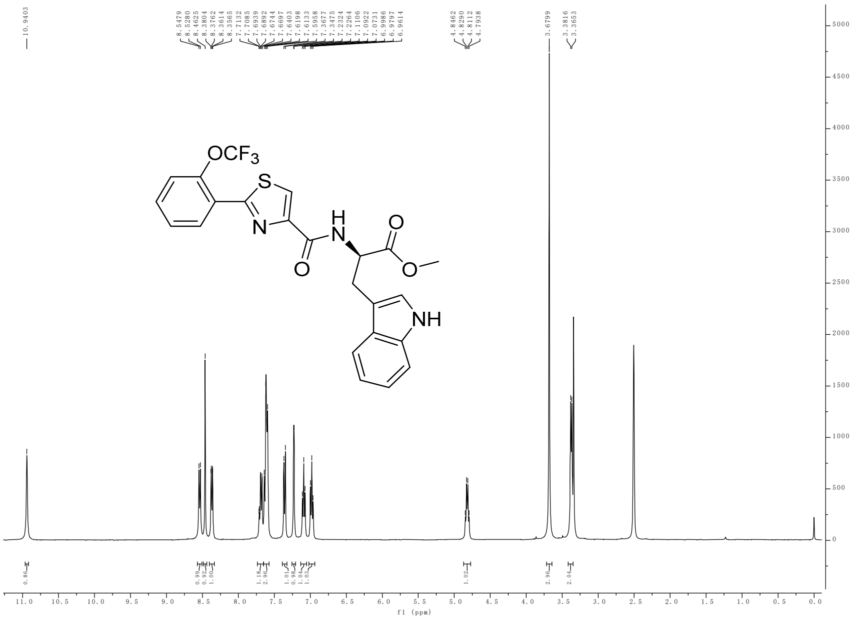

Figure S52. ^{1}H-NMR Spectrum of compound **8r**

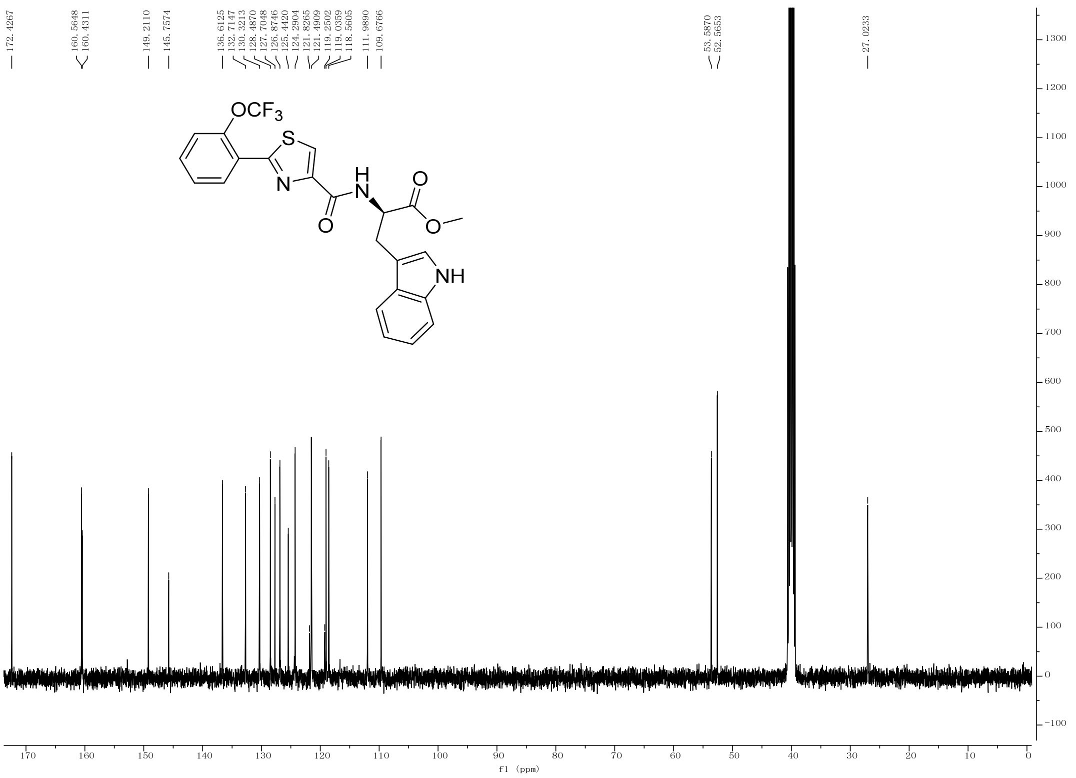

Figure S53. ^{13}C-NMR Spectrum of compound **8r**

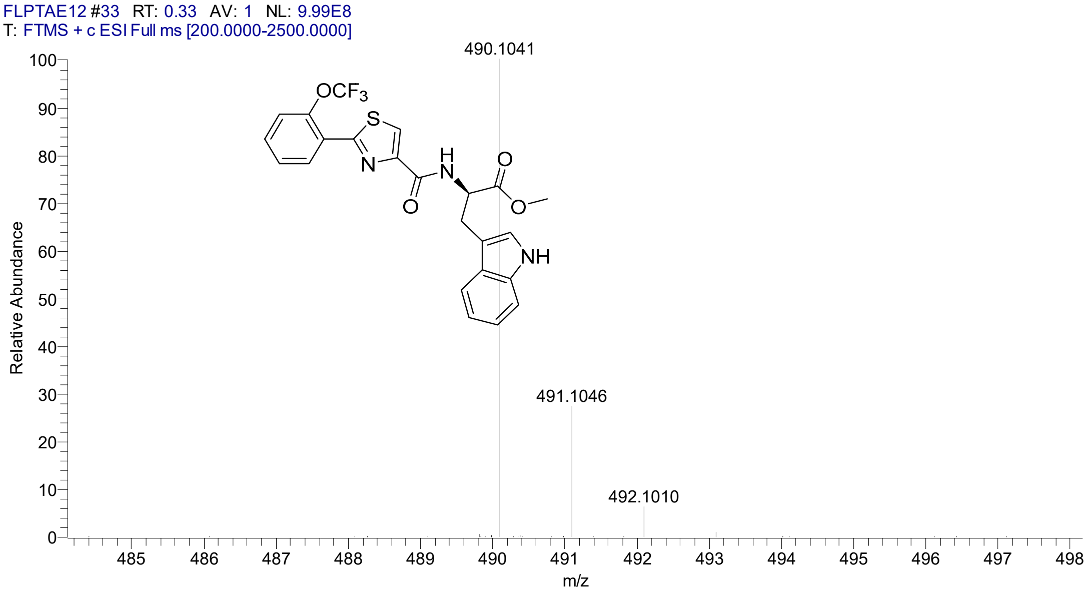

Figure S54. HRMS Spectrum of compound **8r**

9a: white solid; yield 86%, melting point ＞280°C; ^{1}H NMR (400 MHz, DMSO-d_6) δ 8.48 – 8.35 (m, 2H), 8.24 (t, J = 4.8 Hz, 1H), 7.76 – 7.66 (m, 1H), 7.61 (d, J = 8.0 Hz, 2H), 3.54 (d, J = 4.4 Hz, 2H); ^{13}C NMR (101 MHz, DMSO-d_6) δ 170.40, 160.35, 159.59, 150.62, 145.74, 132.63, 130.12, 128.61, 125.72, 121.67, 94.81, 81.24, 44.49. HRMS(ESI) calcd for $C_{13}H_{10}F_3N_2O_4S^+$ [M+H]$^+$: 347.0308, found 347.0297.

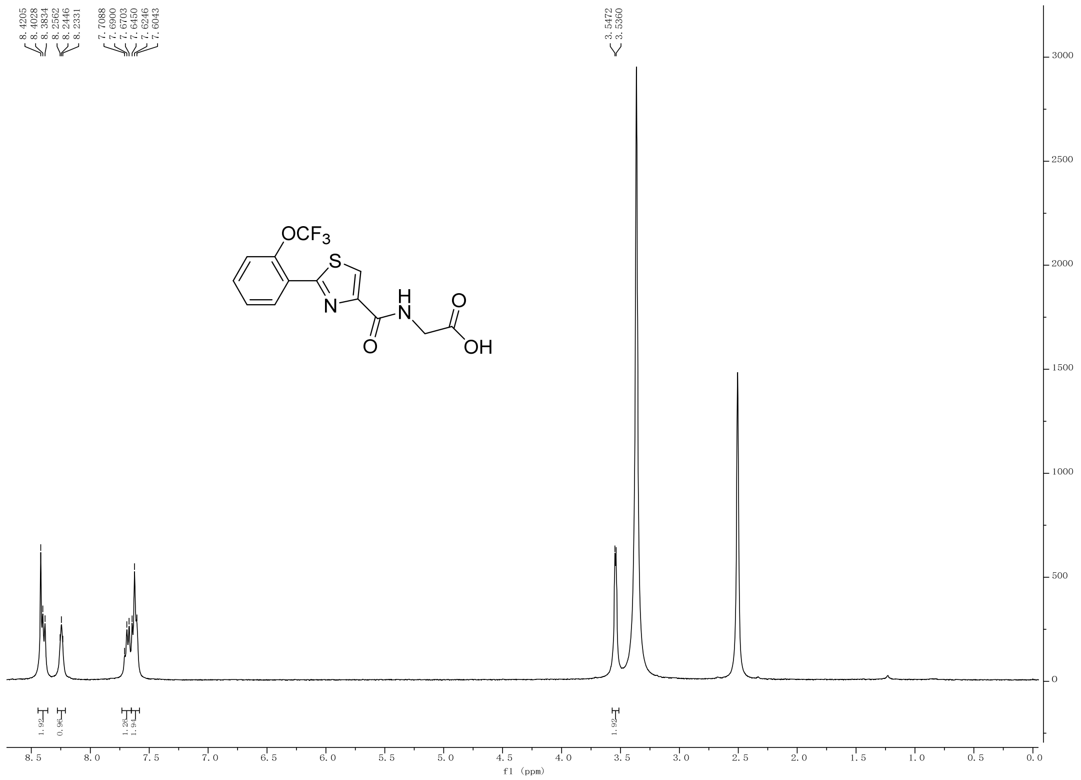

Figure S55. ^{1}H-NMR Spectrum of compound **9a**

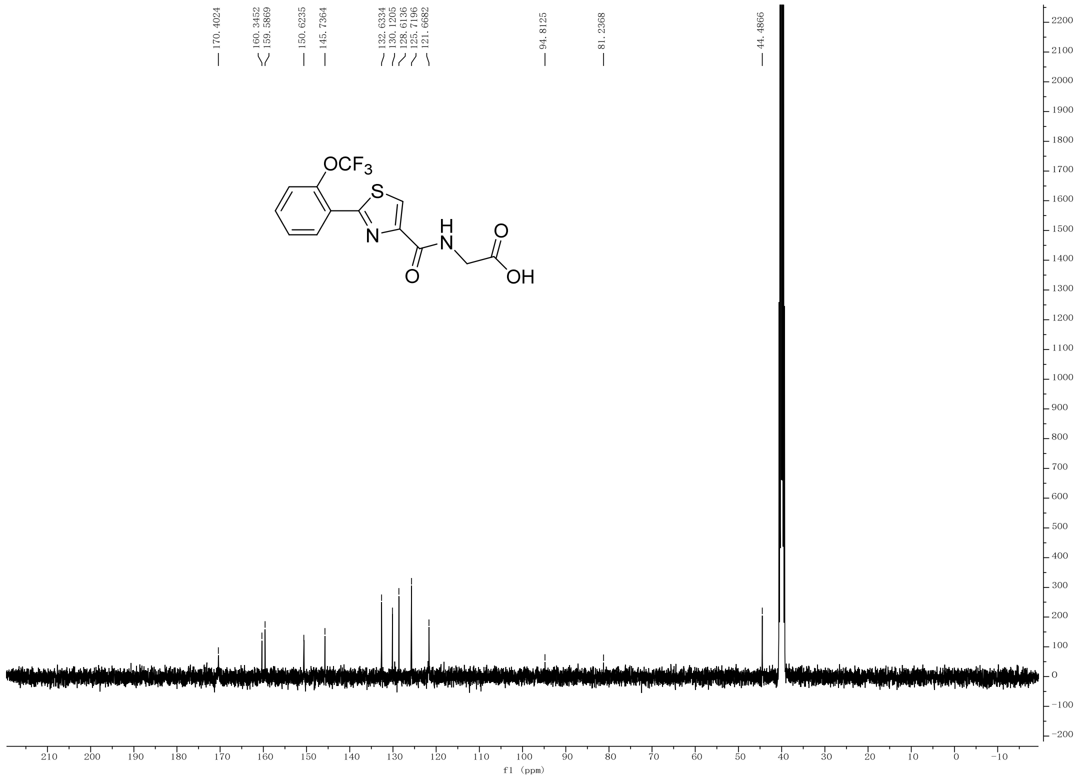

Figure S56. ^{13}C-NMR Spectrum of compound **9a**

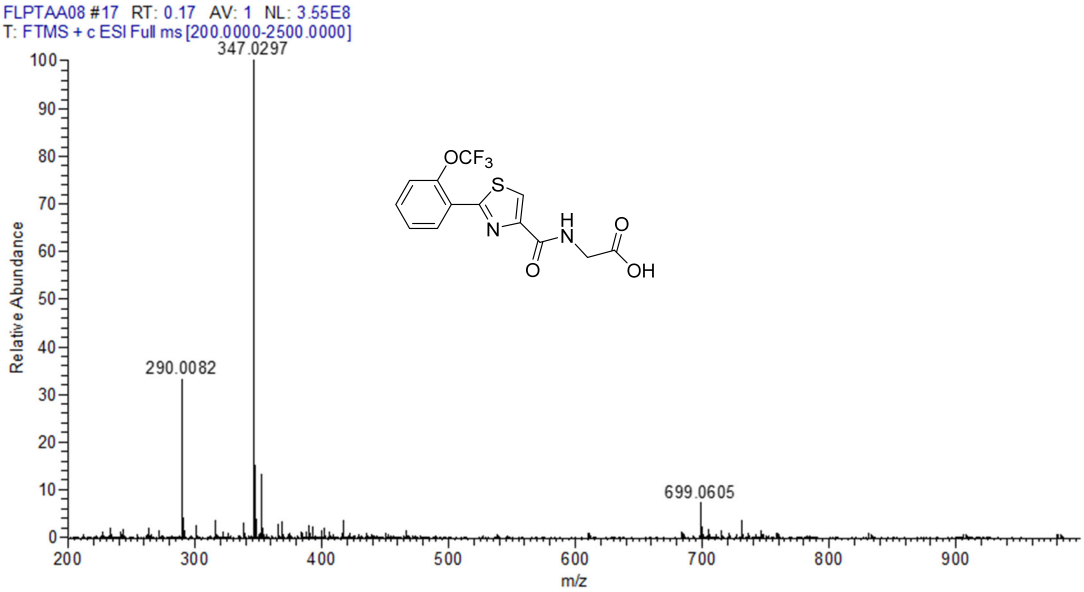

Figure S57. HRMS Spectrum of compound **9a**

9b: white solid; yield 77%, melting point 156.4~160°C; ^{1}H NMR (400 MHz, DMSO-d_6) δ 8.48 (d, J = 6.4 Hz, 1H), 8.43 (s, 1H), 8.38 (d, J = 7.6 Hz, 1H), 7.70 – 7.61 (m, 1H), 7.59 (d, J = 7.2 Hz, 2H), 4.23 – 4.09 (m, 1H), 1.37 (d, J = 6.8 Hz, 3H); ^{13}C NMR (101 MHz, DMSO-d_6) δ 160.37, 159.38, 150.58, 145.73, 132.59, 130.15, 128.53, 125.90, 125.63, 121.82, 121.62, 119.24, 50.17, 19.55. HRMS(ESI) calcd for $C_{14}H_{12}F_3N_2O_4S^+$ $[M+H]^+$: 361.0464, found 361.0461.

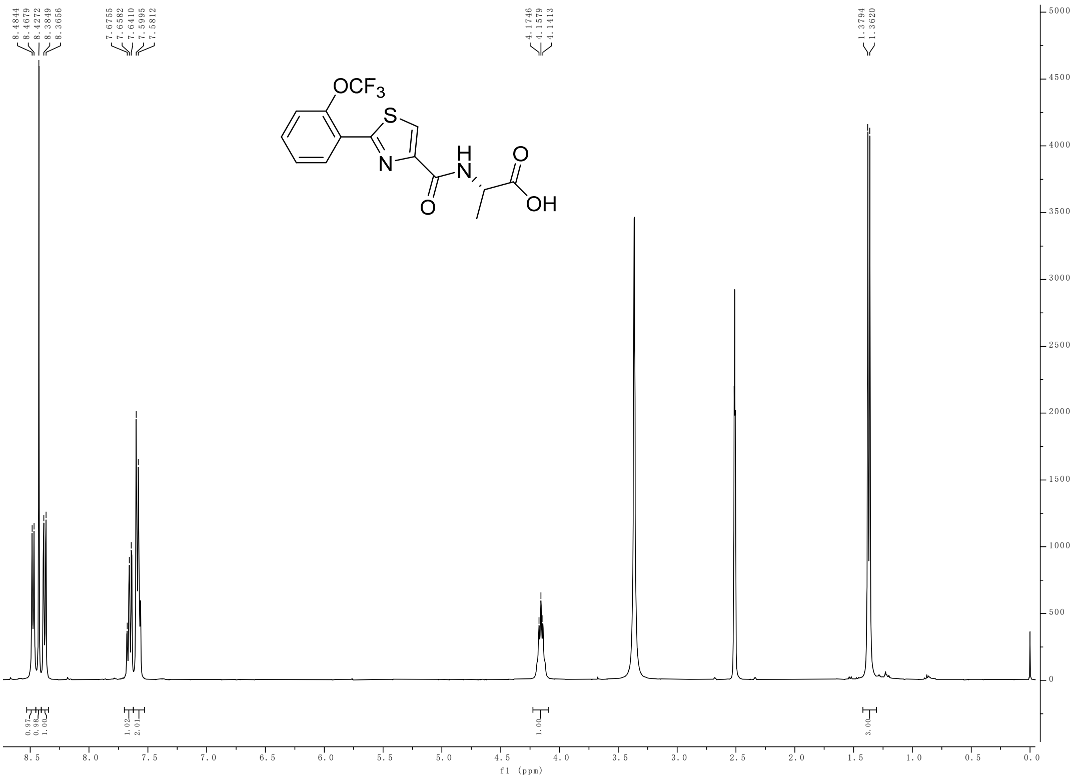

Figure S58. ^{1}H-NMR Spectrum of compound **9b**

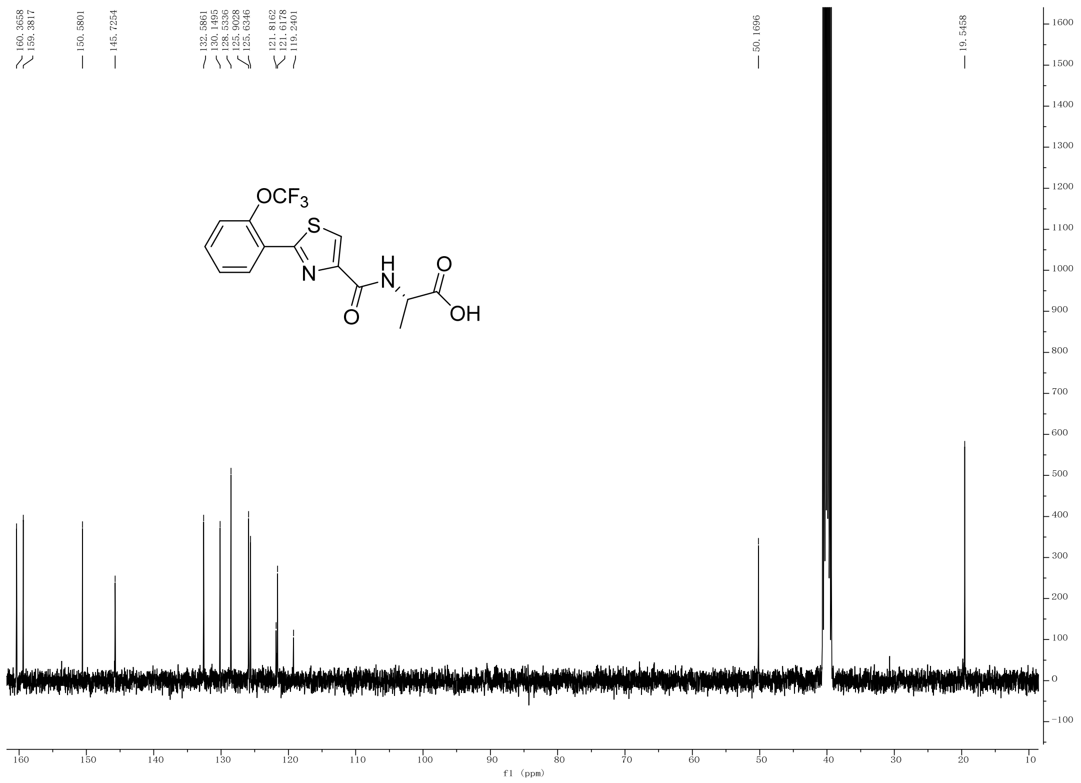

Figure S59. ^{13}C-NMR Spectrum of compound **9b**

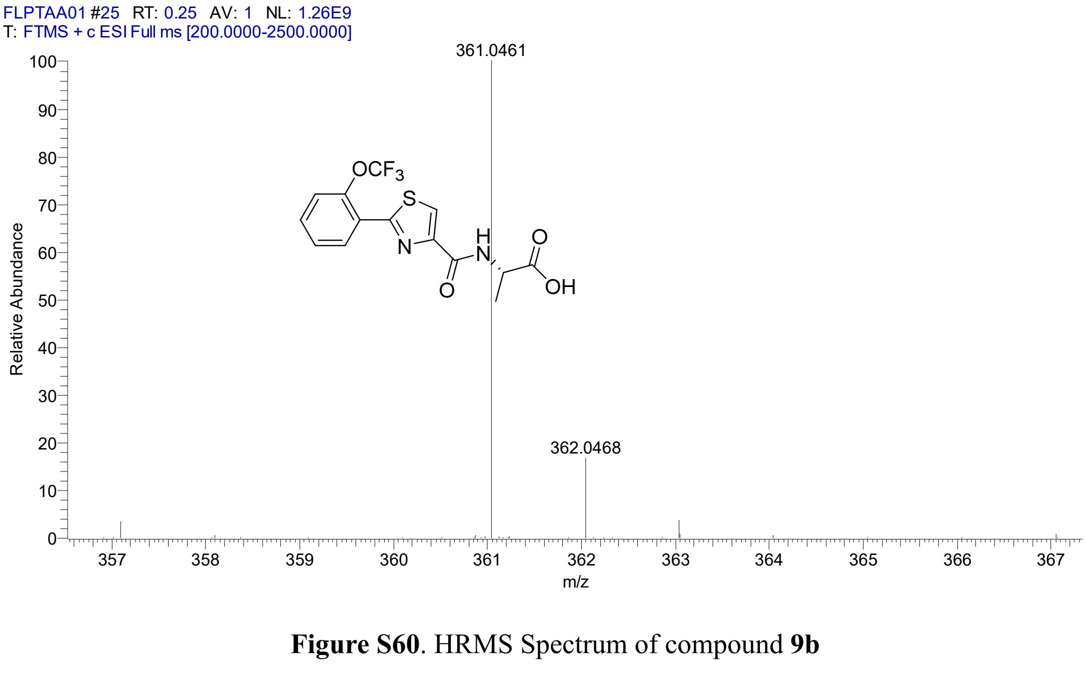

Figure S60. HRMS Spectrum of compound **9b**

9c: white solid; yield 72%, melting point 143.2~145.9°C; ^{1}H NMR (400 MHz, DMSO-*d*6) δ 8.72 (d, J = 8.0 Hz, 1H), 8.59 (dd, J = 1.6, 7.6 Hz, 1H), 8.52 (s, 1H), 7.73 – 7.67 (m, 1H), 7.66 – 7.57 (m, 2H), 4.67 – 4.57 (m, 1H), 2.65 – 2.49 (m, 3H), 2.22 – 2.12 (m, 2H), 2.08 (s, 3H); ^{13}C NMR (101 MHz, DMSO-*d*6) δ 173.61, 160.77, 160.41,

149.74, 145.76, 132.64, 130.50, 128.44, 126.67, 125.64, 121.84, 121.51, 51.87, 31.03, 30.48, 15.05. HRMS(ESI) calcd for $C_{16}H_{16}F_3N_2O_4S_2^+$ $[M+H]^+$: 421.0498, found 421.0490.

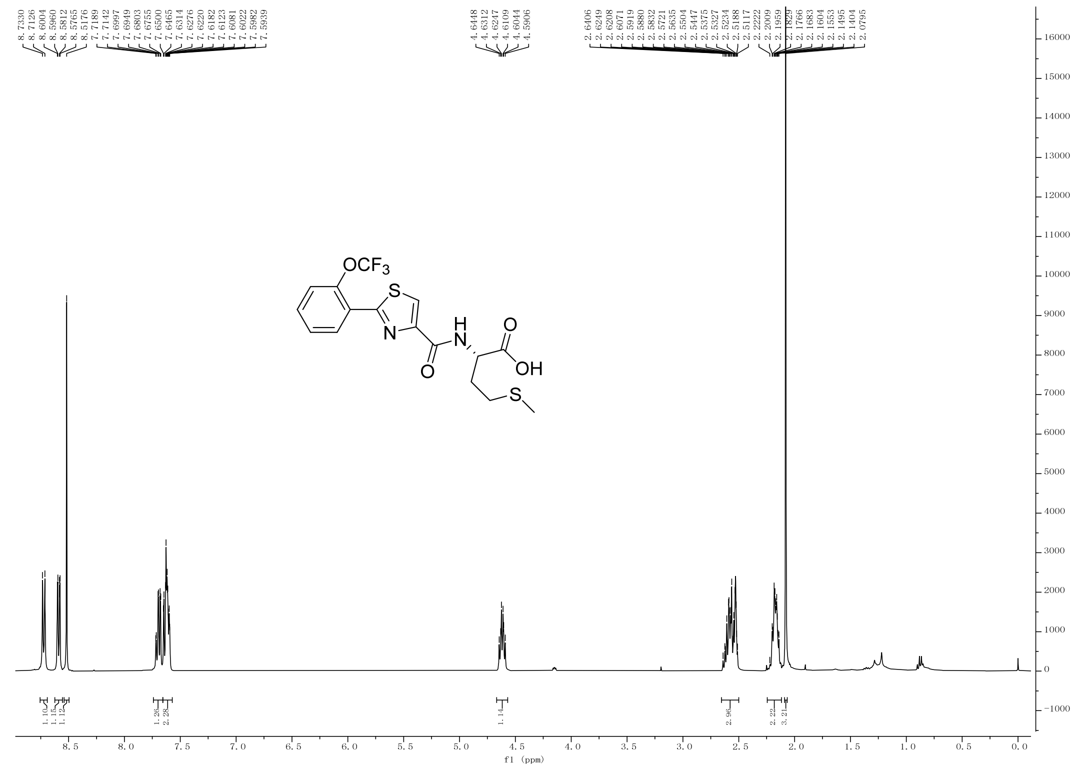

Figure S61. ^{1}H-NMR Spectrum of compound **9c**

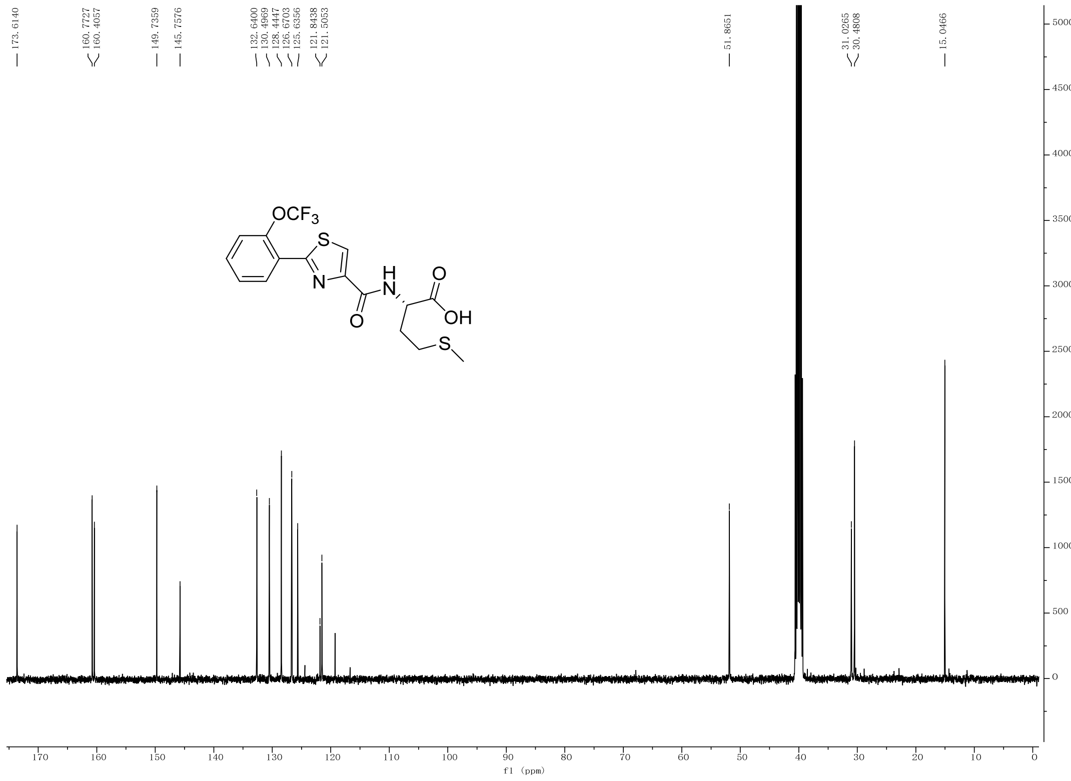

Figure S62. ^{13}C-NMR Spectrum of compound **9c**

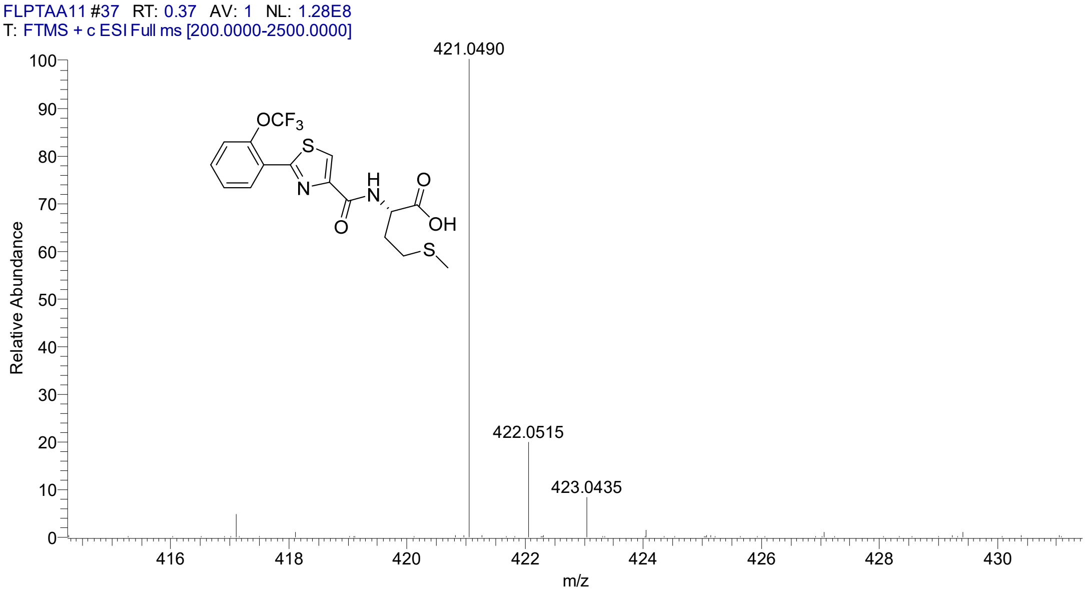

Figure S63. HRMS Spectrum of compound **9c**

9d: white solid; yield 70%, melting point 185.3~187.1°C; ^{1}H NMR (400 MHz, DMSO-d_6) δ 8.45 (s, 1H), 8.41 – 8.27 (m, 2H), 7.73 – 7.65 (m, 1H), 7.65 – 7.55 (m, 2H), 4.18 (dd, J = 4.4, 8.0 Hz, 1H), 2.01 – 1.87 (m, 1H), 1.66 – 1.53 (m, 1H), 1.27 – 1.15 (m, 1H), 0.88 (t, J = 7.6 Hz, 6H); ^{13}C NMR (101 MHz, DMSO-d_6) δ 174.17, 160.45, 159.52, 150.75, 145.69, 132.60, 130.12, 128.61, 125.84, 125.68, 121.82, 121.72, 119.25, 58.83, 38.40, 25.52, 16.37, 12.35. HRMS(ESI) calcd for $C_{17}H_{18}F_3N_2O_4S^+$ [M+H]$^+$: 403.0934, found 403.0930.

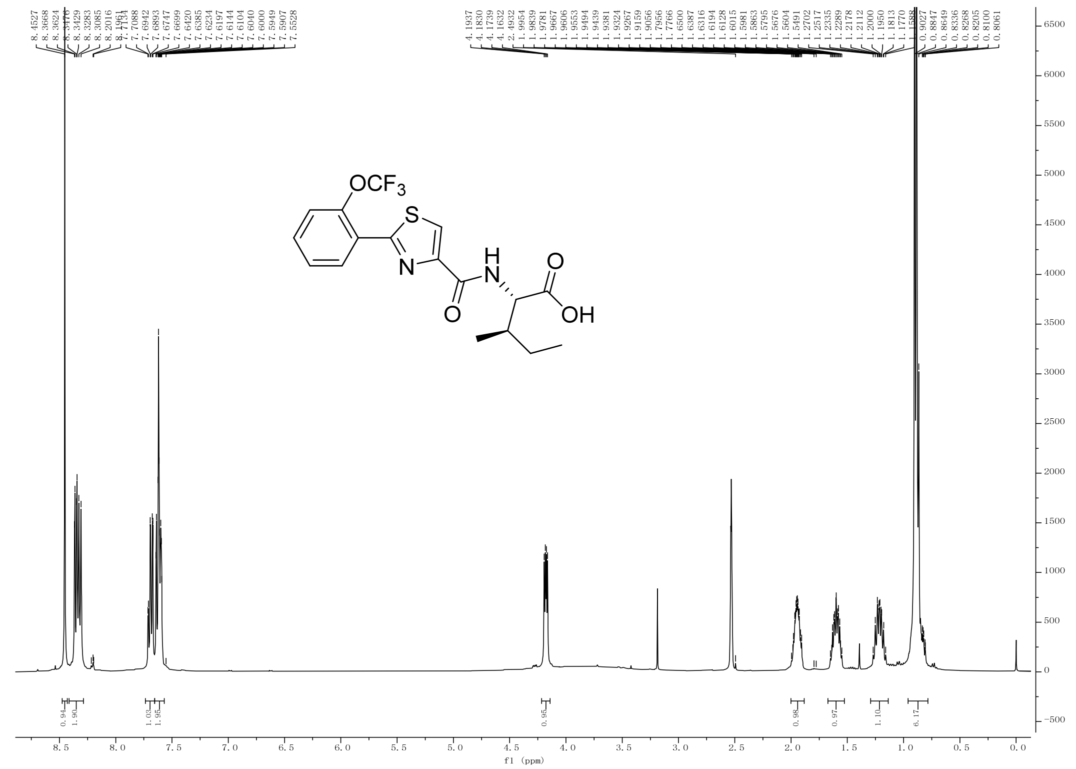

Figure S64. ^{1}H-NMR Spectrum of compound **9d**

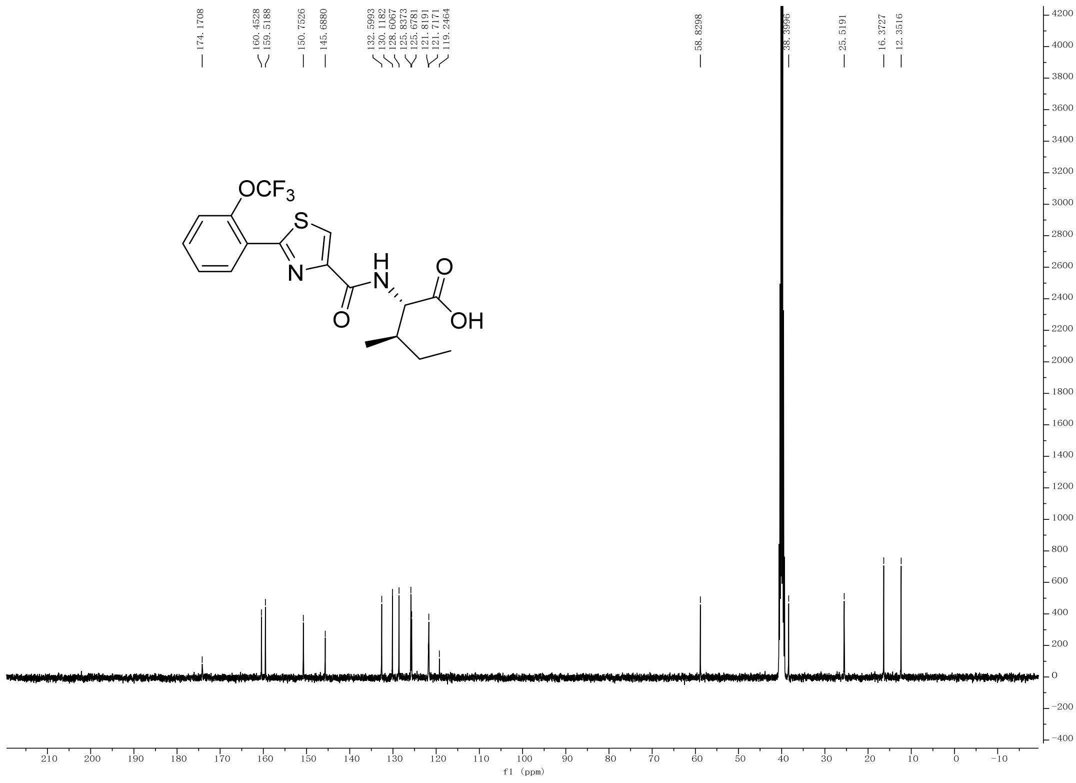

Figure S65. ^{13}C-NMR Spectrum of compound **9d**

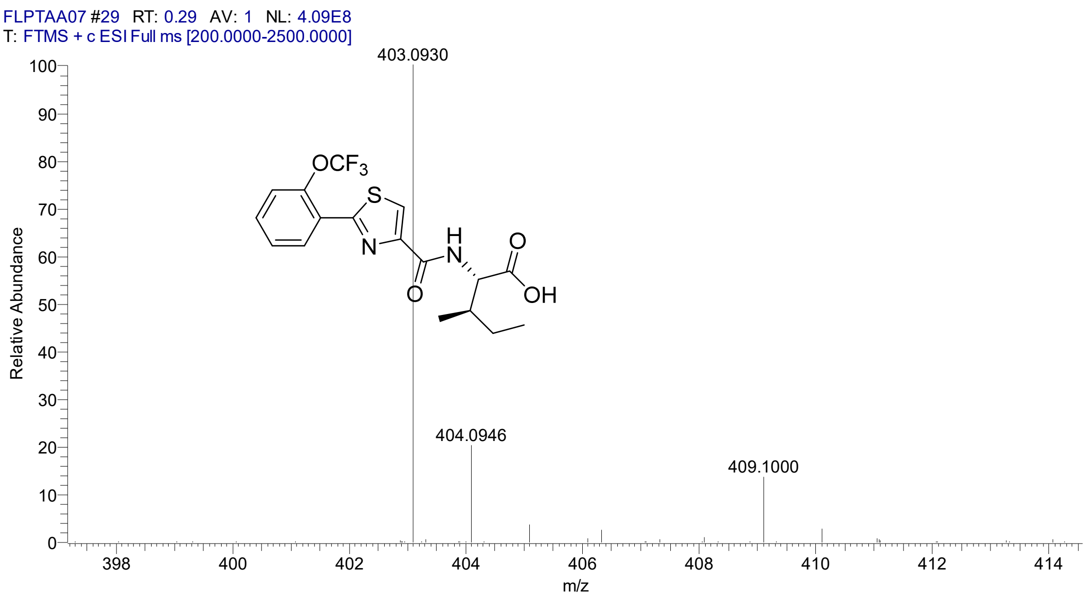

Figure S66. HRMS Spectrum of compound **9d**

9e: white solid; yield 79%, melting point 148.5~149.3°C; ^{1}H NMR (400 MHz, DMSO-d_6) δ 8.53 (s, 1H), 8.47 (dd, J = 1.6, 7.6 Hz, 1H), 8.19 (d, J = 8.8 Hz, 1H), 7.74 – 7.67 (m, 1H), 7.66 – 7.57 (m, 2H), 4.40 (dd, J = 5.6, 8.8 Hz, 1H), 2.32 – 2.21 (m, J = 6.8 Hz, 1H), 0.97 (dd, J = 2.4, 6.8 Hz, 6H); ^{13}C NMR (101 MHz, DMSO-d_6) δ 173.12, 160.91, 160.58, 149.60, 145.68, 132.75, 130.60, 128.67, 126.81, 125.61, 121.80, 119.29, 57.77, 40.61, 40.40, 40.20, 39.99, 39.78, 39.57, 39.36, 30.56, 19.67, 18.57. HRMS(ESI) calcd for $C_{16}H_{16}F_3N_2O_4S^+$ [M+H]$^+$: 389.0777, found 389.0775.

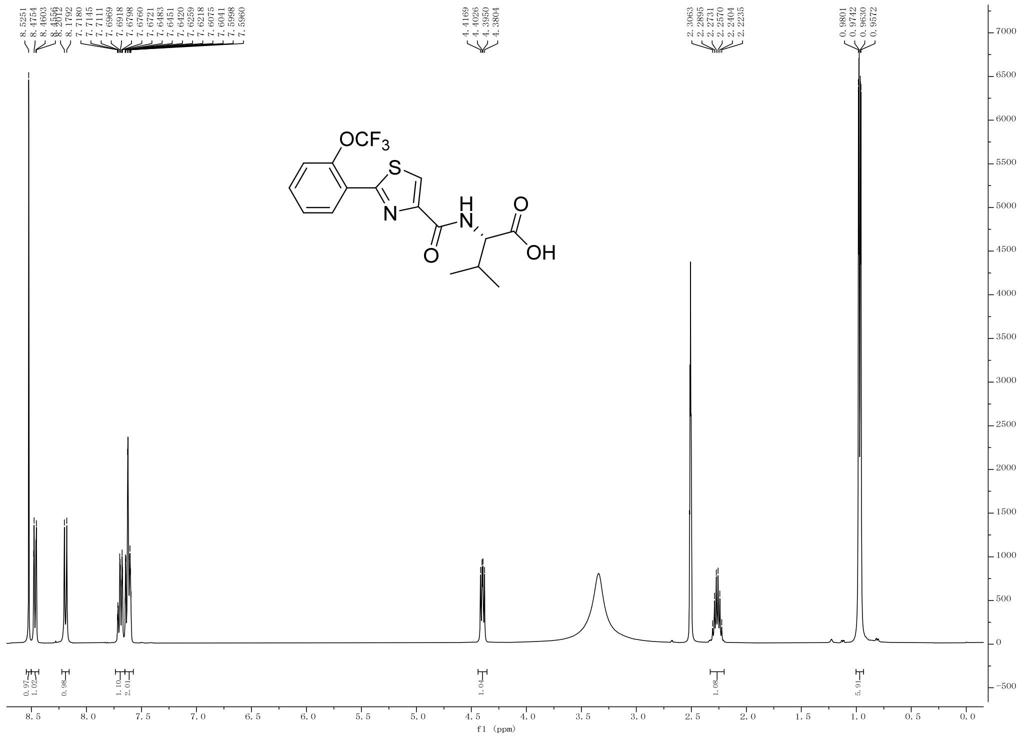

Figure S67. ^{1}H-NMR Spectrum of compound **9e**

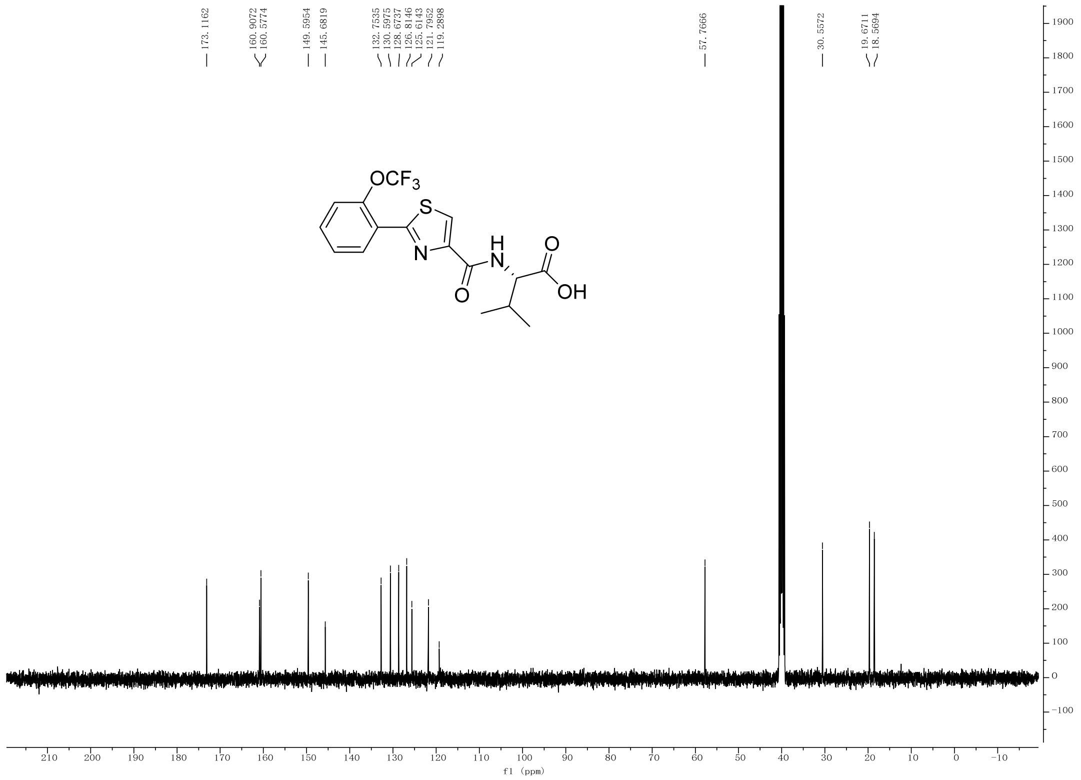

Figure S68. ^{13}C-NMR Spectrum of compound **9e**

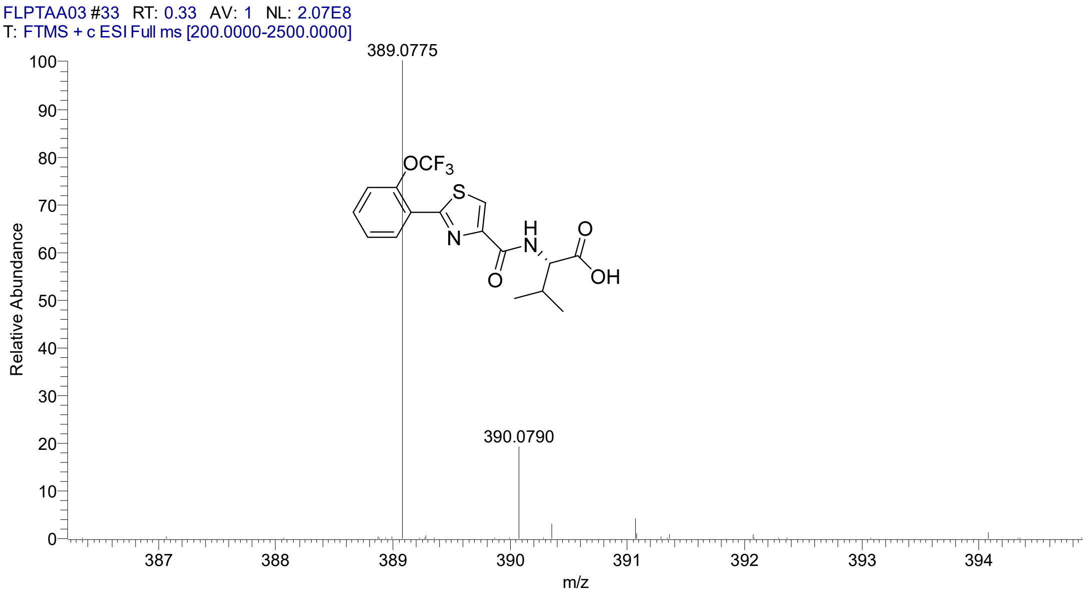

Figure S69. HRMS Spectrum of compound **9e**

9f: white solid; yield 74%, melting point 150.7~152.5 °C; ^{1}H NMR (400 MHz, DMSO-d_6) δ 12.79 (s, 1H), 8.59 (dd, J = 2.0, 8.0 Hz, 2H), 8.49 (s, 1H), 7.73 – 7.65 (m, 1H), 7.67 – 7.53 (m, 2H), 4.57 – 4.47 (m, 1H), 1.92 – 1.77 (m, 1H), 1.74 – 1.58 (m, 2H), 0.92 (dd, J = 6.0, 8.8 Hz, 6H); ^{13}C NMR (101 MHz, DMSO-d_6) δ 174.26, 160.70,

160.47, 149.71, 145.76, 132.68, 130.62, 128.49, 126.70, 125.66, 121.86, 121.58, 119.28, 50.85, 24.97, 23.43, 21.76. HRMS(ESI) calcd for $C_{17}H_{18}F_3N_2O_4S^+$ $[M+H]^+$: 403.0934, found 403.0934.

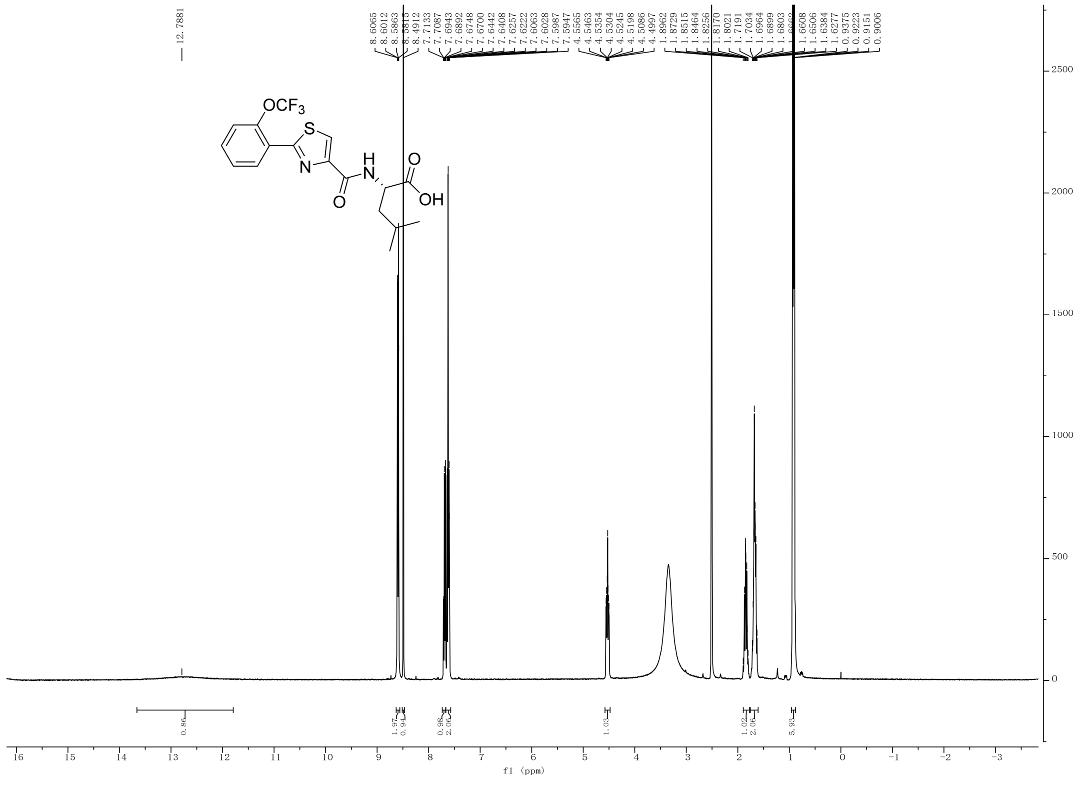

Figure S70. ^{1}H-NMR Spectrum of compound **9f**

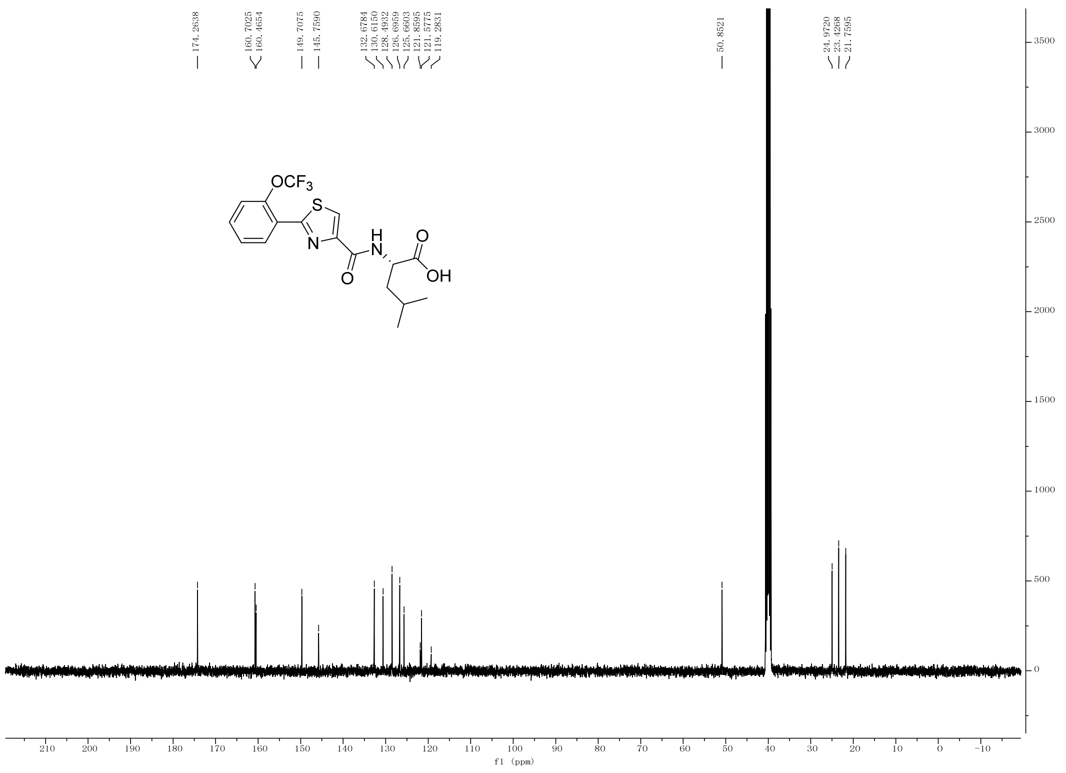

Figure S71. ^{13}C-NMR Spectrum of compound **9f**

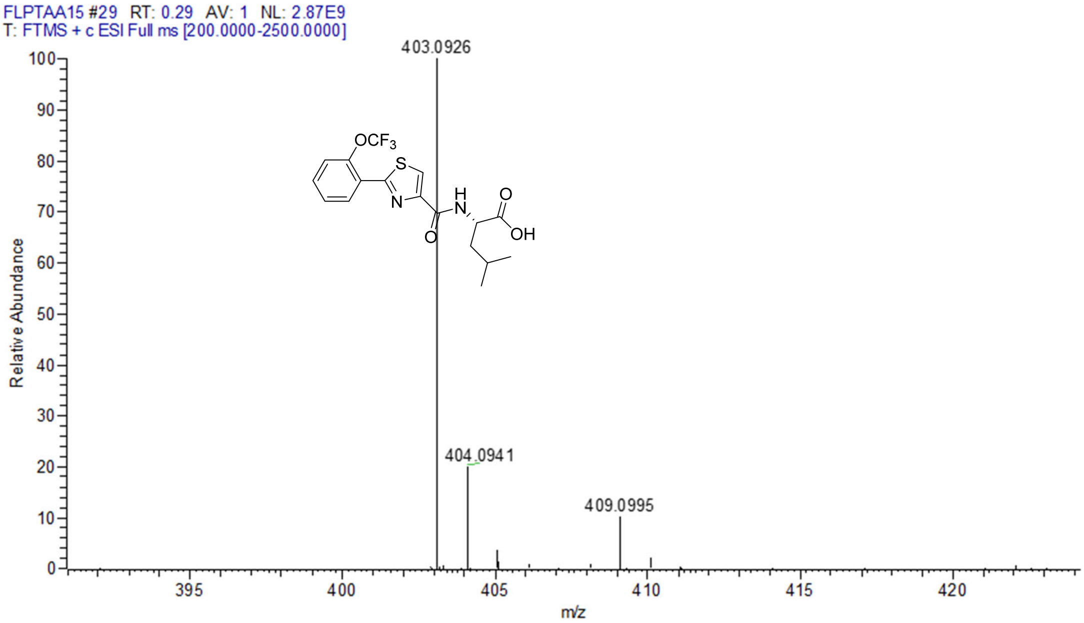

Figure S72. HRMS Spectrum of compound **9f**

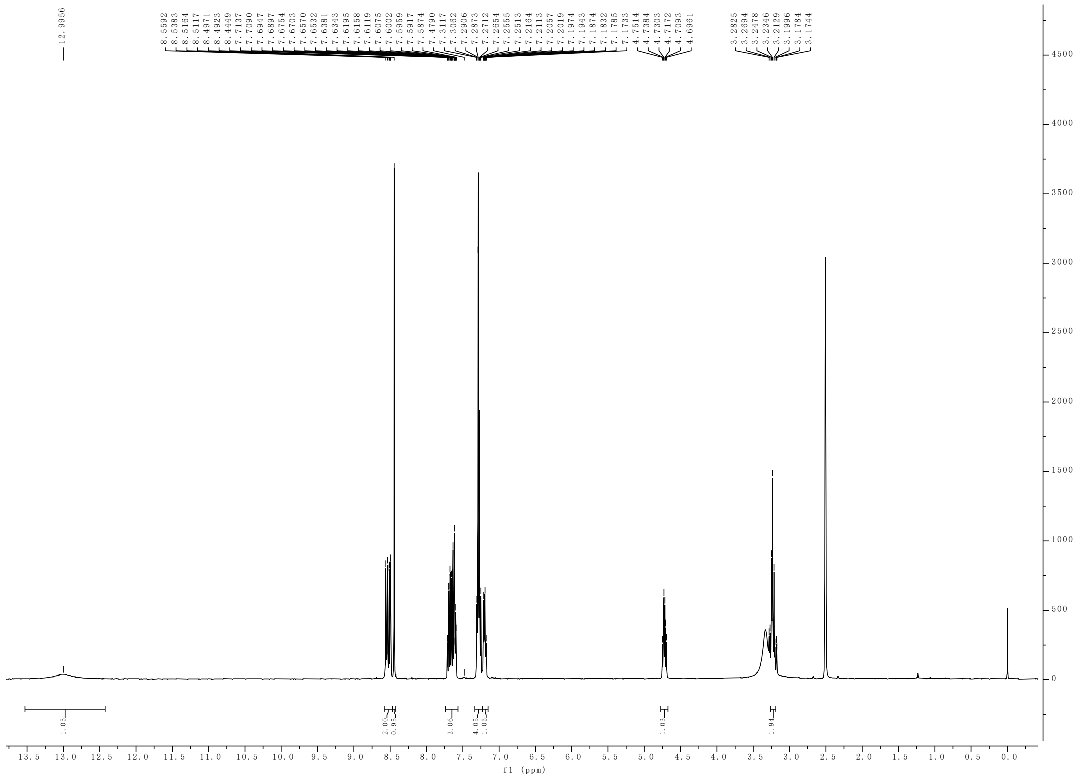

Figure S73. ^{1}H-NMR Spectrum of compound **9g**

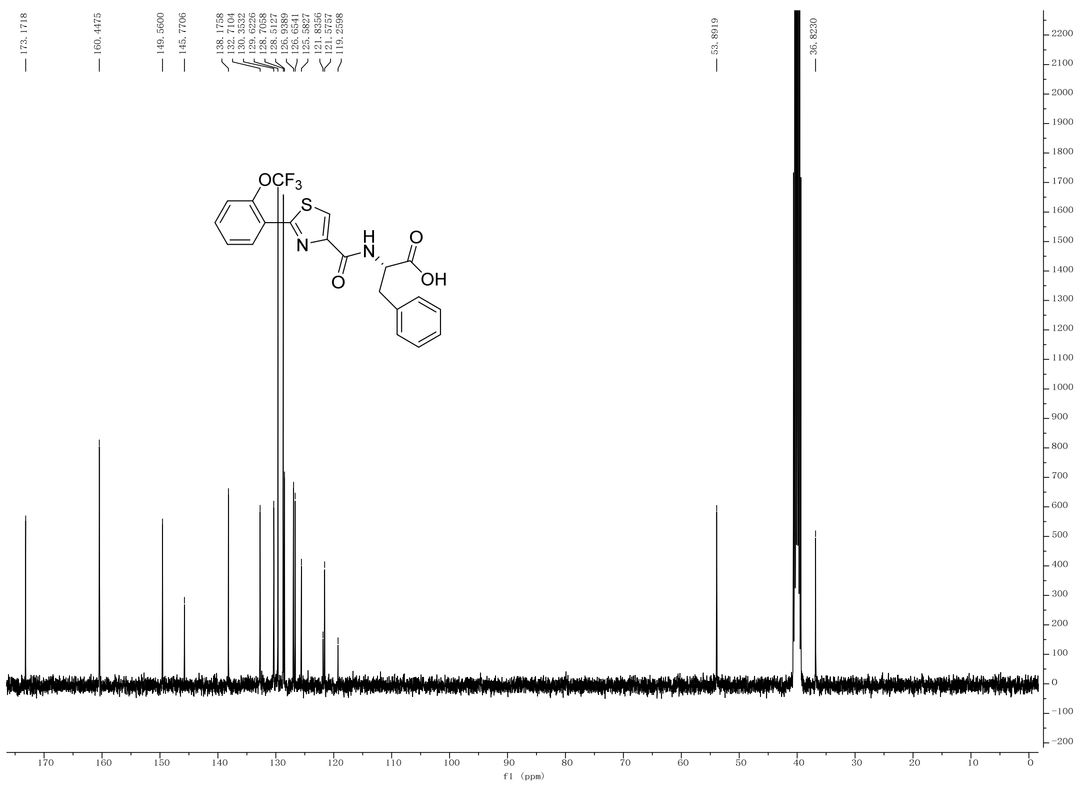

Figure S74. ^{13}C-NMR Spectrum of compound **9g**

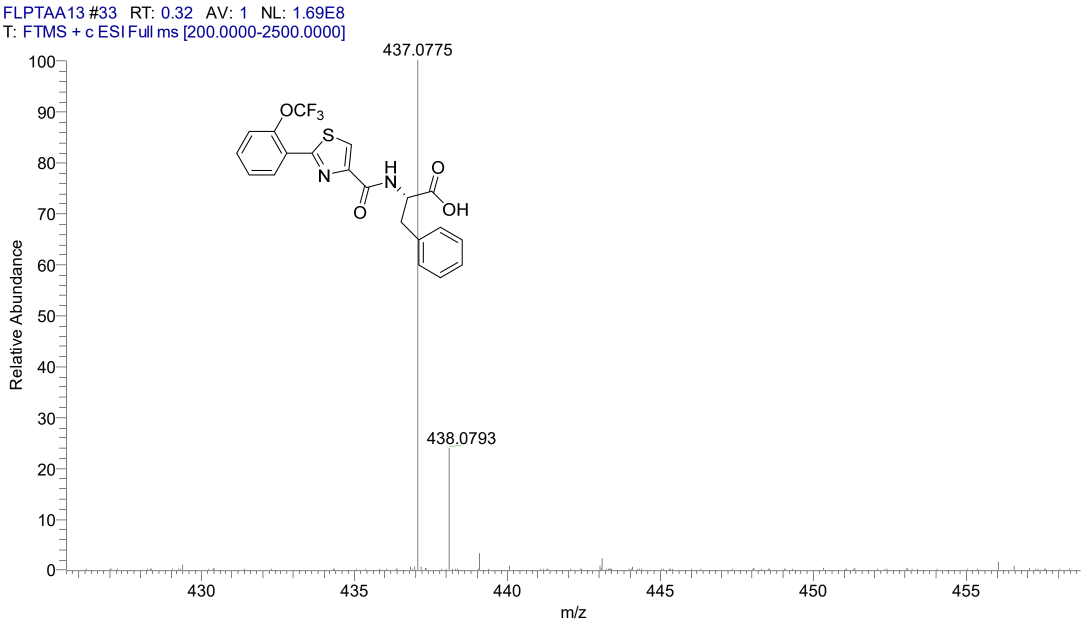

Figure S75. HRMS Spectrum of compound **9g**

9h: white solid; yield 74%, melting point 269.7~272.3°C; ^{1}H NMR (400 MHz, DMSO-d_6) δ 8.42 (s, 1H), 8.35 (dd, J = 1.6, 8.0 Hz, 1H), 8.24 (d, J = 7.6 Hz, 1H), 7.64 (d, J = 7.6 Hz, 1H), 7.57 (t, J = 6.6 Hz, 2H), 7.01 (d, J = 8.0 Hz, 2H), 6.66 – 6.56 (m,

2H), 4.47 (s, 1H), 3.19 – 3.02 (m, 2H); ^{13}C NMR (101 MHz, DMSO-d_6) δ 160.33, 159.81, 156.28, 150.24, 145.72, 132.60, 130.74, 130.15, 128.61, 128.52, 126.05, 125.55, 124.39, 121.82, 121.53, 119.24, 116.67, 115.31, 55.25, 36.73. HRMS(ESI) calcd for $C_{20}H_{16}F_3N_2O_5S^+$ [M+H]$^+$: 453.0727, found 453.0730.

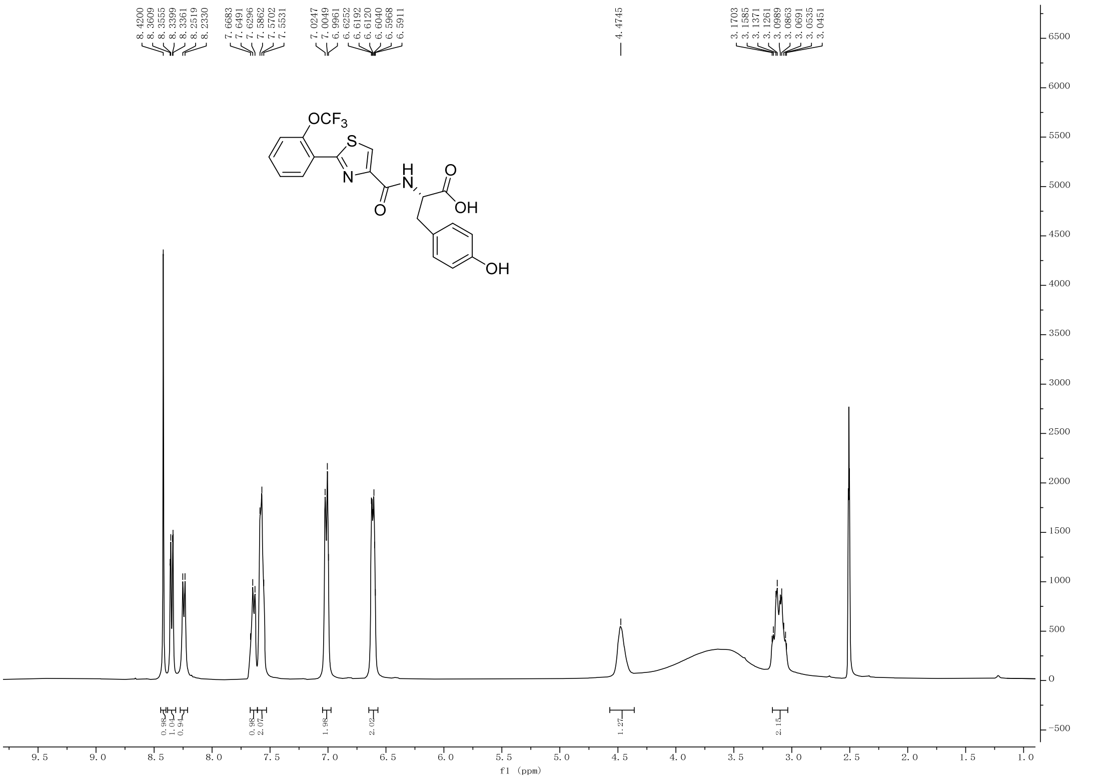

Figure S76. ^{1}H-NMR Spectrum of compound **9h**

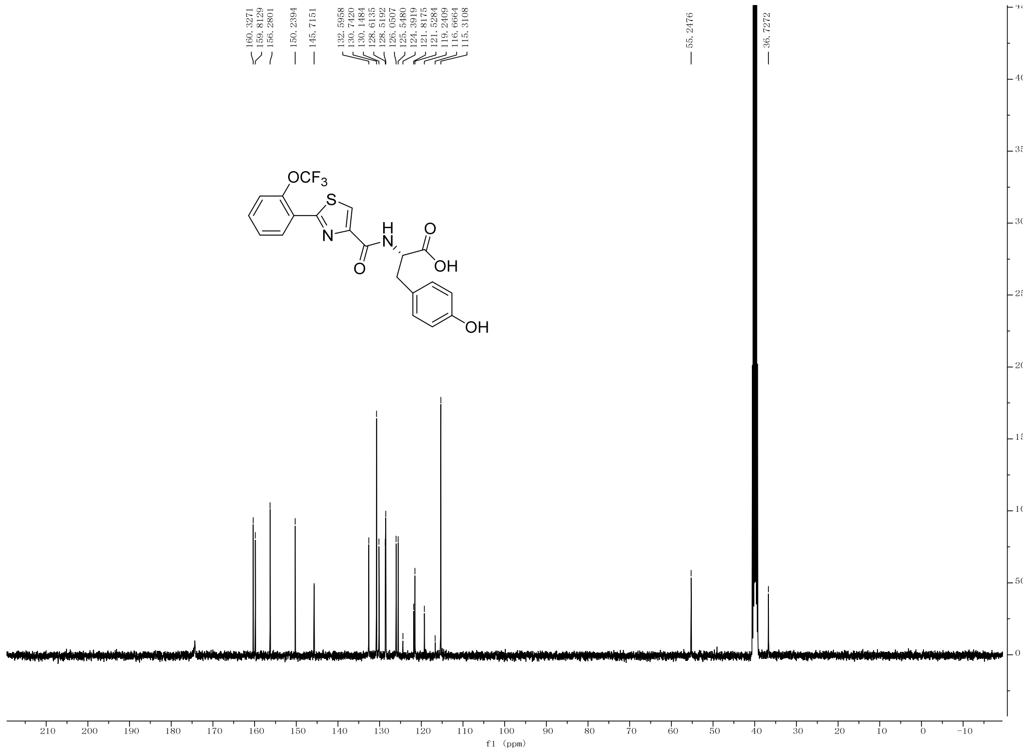

Figure S77. ^{13}C-NMR Spectrum of compound **9h**

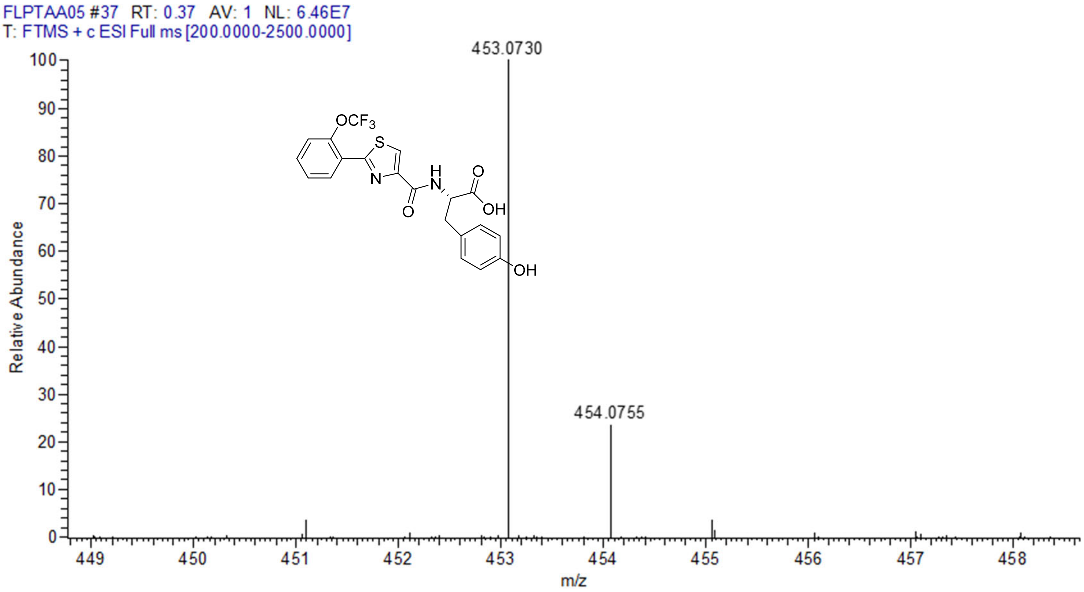

Figure S78. HRMS Spectrum of compound **9h**

9i: white solid; yield 78%, melting point 146.8 ~ 147.8°C; ^{1}H NMR (400 MHz, DMSO-*d*$_6$) δ 10.91 (d, J = 2.6 Hz, 1H), 8.45 (s, 1H), 8.41 – 8.27 (m, 2H), 7.74 – 7.55 (m, 4H), 7.35 (d, J = 8.0 Hz, 1H), 7.21 (d, J = 2.4 Hz, 1H), 7.12 – 7.04 (m, 1H), 7.02 – 6.92 (m, 1H), 4.79 – 4.68 (m, 1H), 3.50 – 3.26 (m, 3H); ^{13}C NMR (101 MHz, DMSO-*d*$_6$) δ 173.50, 160.38, 149.54, 145.75, 136.58, 132.69, 130.27, 128.52, 127.87, 126.60, 125.48, 124.18, 121.83, 121.54, 121.44, 119.25, 118.94, 118.75, 111.90, 110.10, 53.59, 27.13. HRMS(ESI) calcd for $C_{22}H_{17}F_3N_3O_4S^+$ [M+H]$^+$: 476.0886, found 476.0883.

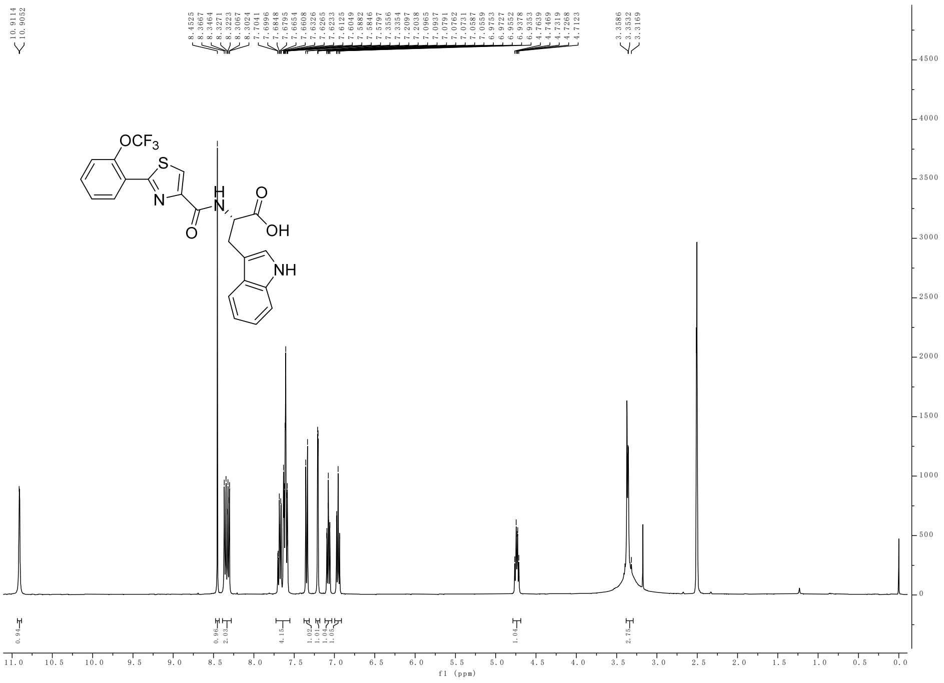

Figure S79. ^{1}H-NMR Spectrum of compound **9i**

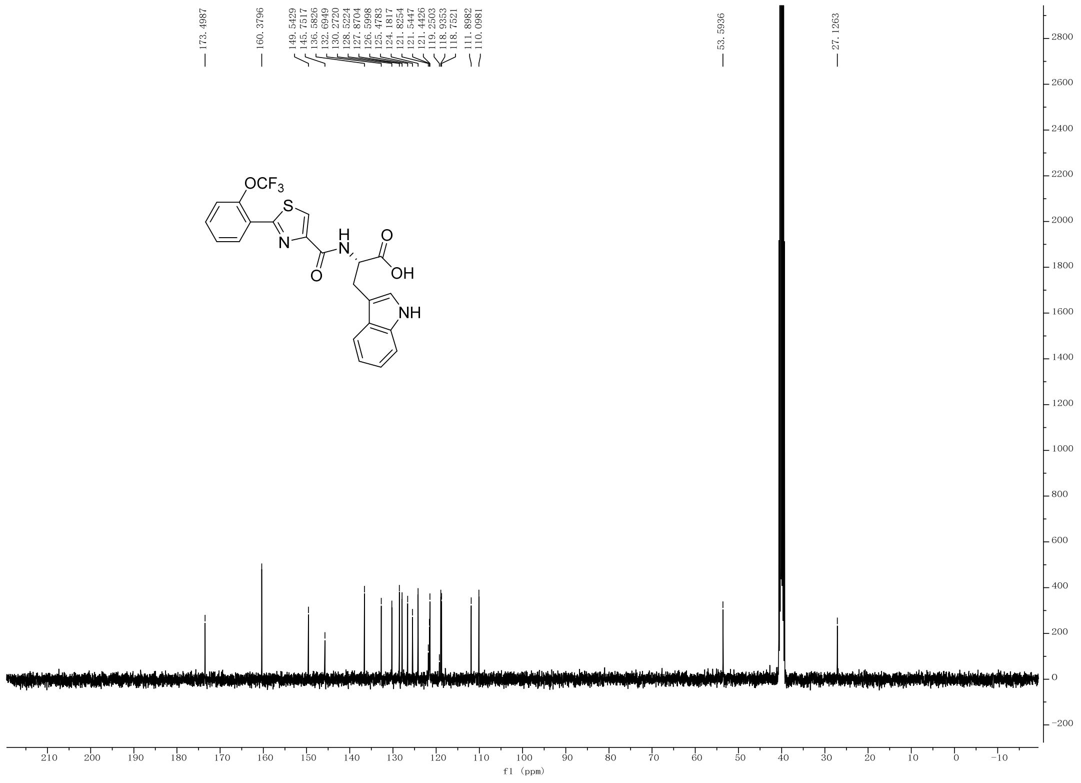

Figure S80. ^{13}C-NMR Spectrum of compound **9i**

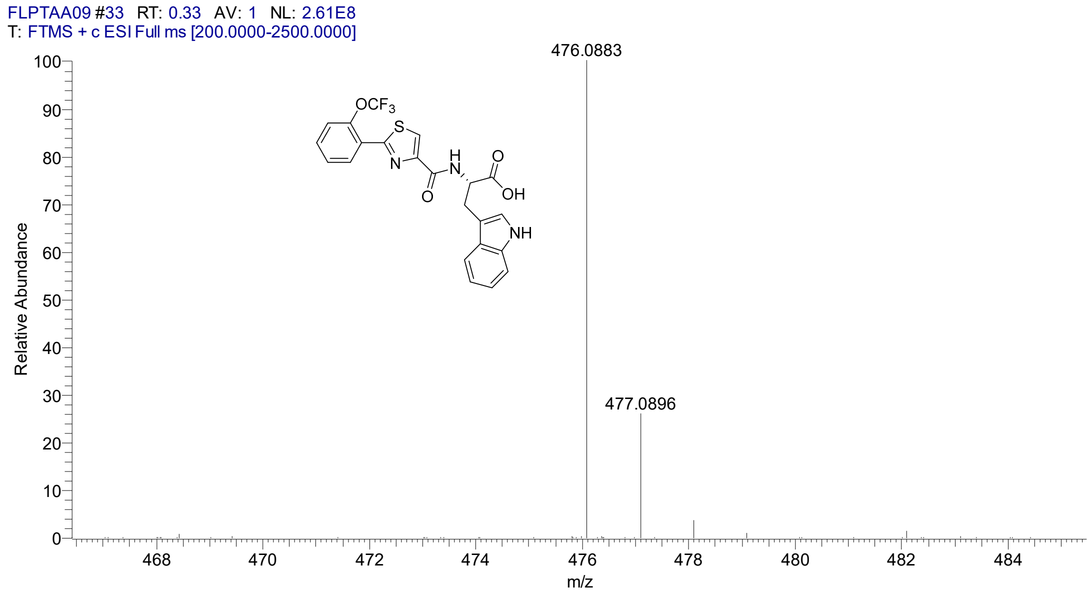

Figure S81. HRMS Spectrum of compound **9i**

9j: white solid; yield 78%, melting point 161.7~163.4°C; ^{1}H NMR (400 MHz, DMSO-d_6) δ 8.48 (d, J = 6.6 Hz, 1H), 8.43 (s, 1H), 8.38 (dd, J = 1.6, 8.0 Hz, 1H), 7.70 – 7.62 (m, 1H), 7.62 – 7.54 (m, 2H), 4.21 – 4.11 (m, 1H), 1.37 (d, J = 6.8 Hz, 3H); ^{13}C NMR (101 MHz, DMSO-d_6) δ 160.37, 159.38, 150.58, 145.73, 132.59, 130.15, 128.54,

125.90, 125.63, 121.82, 121.62, 119.24, 50.18, 19.55. HRMS(ESI) calcd for $C_{14}H_{12}F_3N_2O_4S^+$ $[M+H]^+$: 361.0464, found 361.0464.

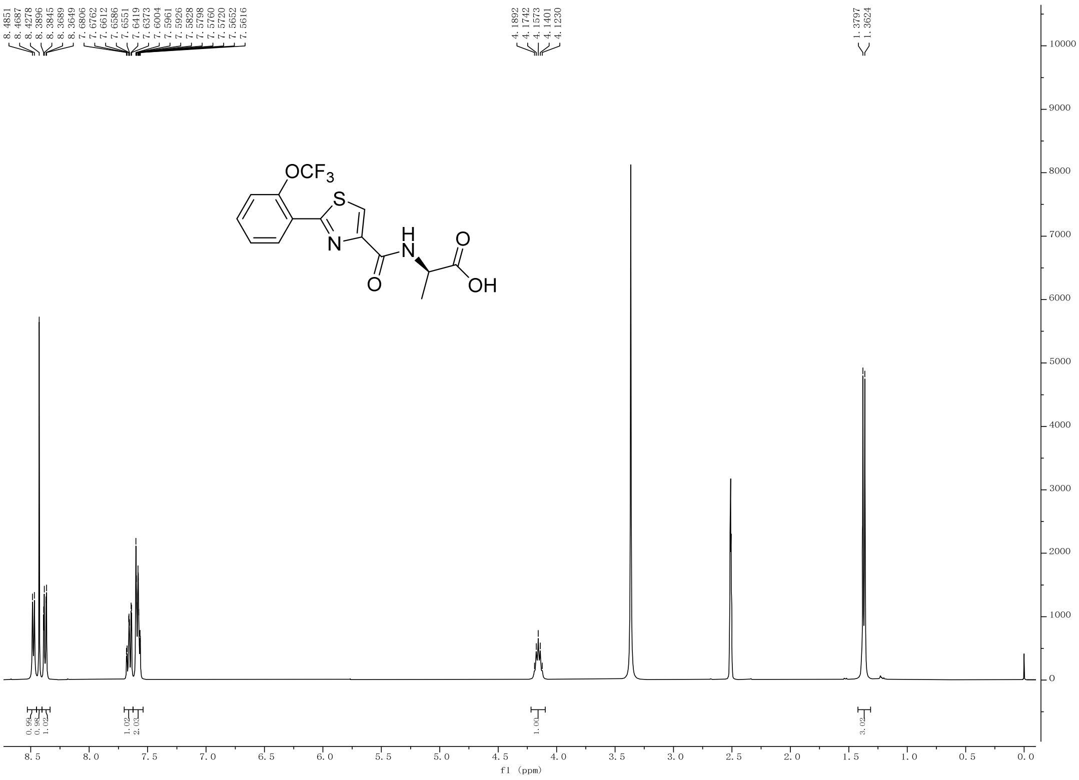

Figure S82. ^{1}H-NMR Spectrum of compound **9j**

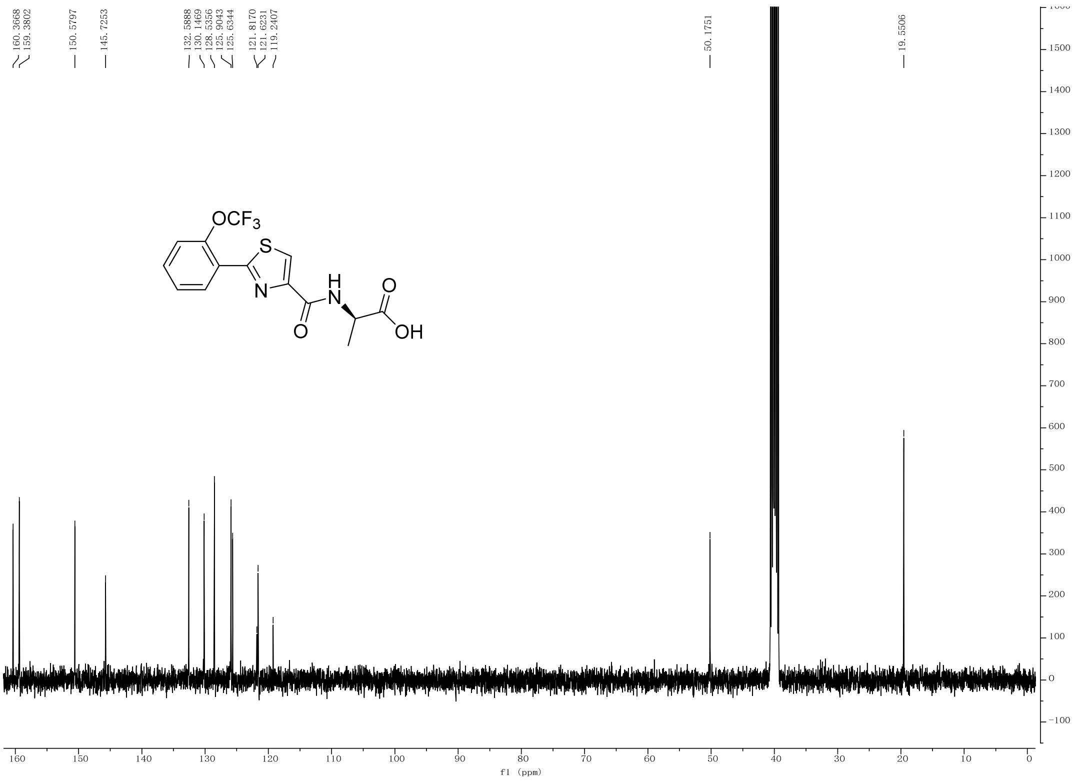

Figure S83. ^{13}C-NMR Spectrum of compound **9j**

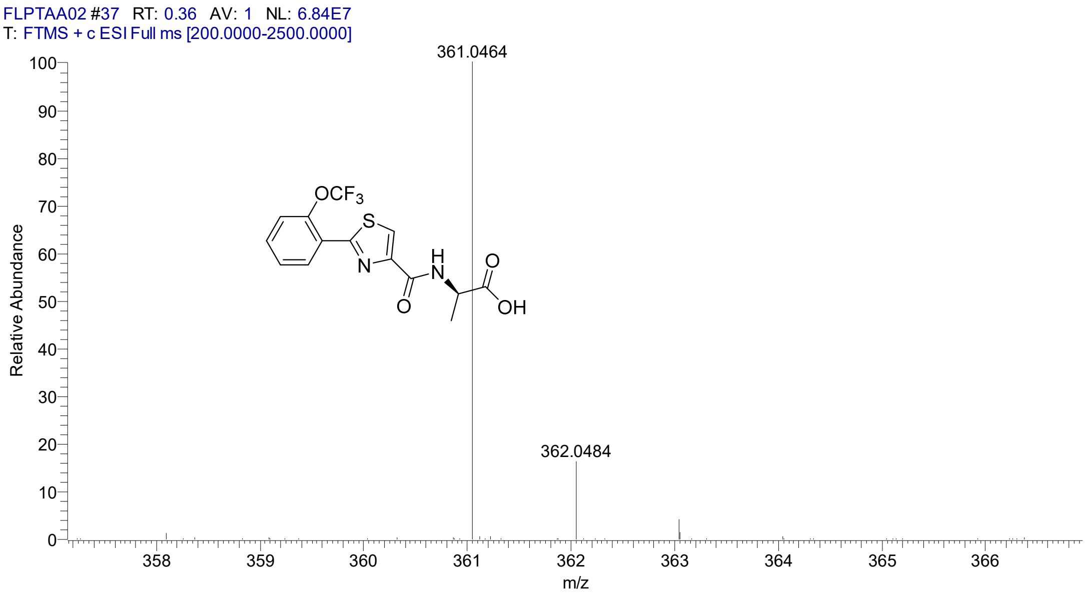

Figure S84. HRMS Spectrum of compound **9j**

9k: white solid; yield 77%, melting point 145.6~147.3 °C; ^{1}H NMR (400 MHz, DMSO-d_6) δ 8.75 (d, J = 8.4 Hz, 1H), 8.60 (dd, J = 1.6, 7.6 Hz, 1H), 8.51 (s, 1H), 7.74 – 7.65 (m, 1H), 7.67 – 7.56 (m, 2H), 4.69 – 4.60 (m, 1H), 2.67 – 2.55 (m, 2H), 2.23 – 2.12 (m, 2H), 2.08 (s, 3H); ^{13}C NMR (101 MHz, DMSO-d_6) δ 173.55, 160.93, 160.42, 149.63, 145.76, 132.66, 130.54, 128.44, 126.77, 125.64, 121.85, 121.52, 51.57, 30.74, 30.50, 15.03. HRMS(ESI) calcd for $C_{16}H_{16}F_3N_2O_4S_2^+$ [M+H]$^+$: 421.0498, found 421.0489.

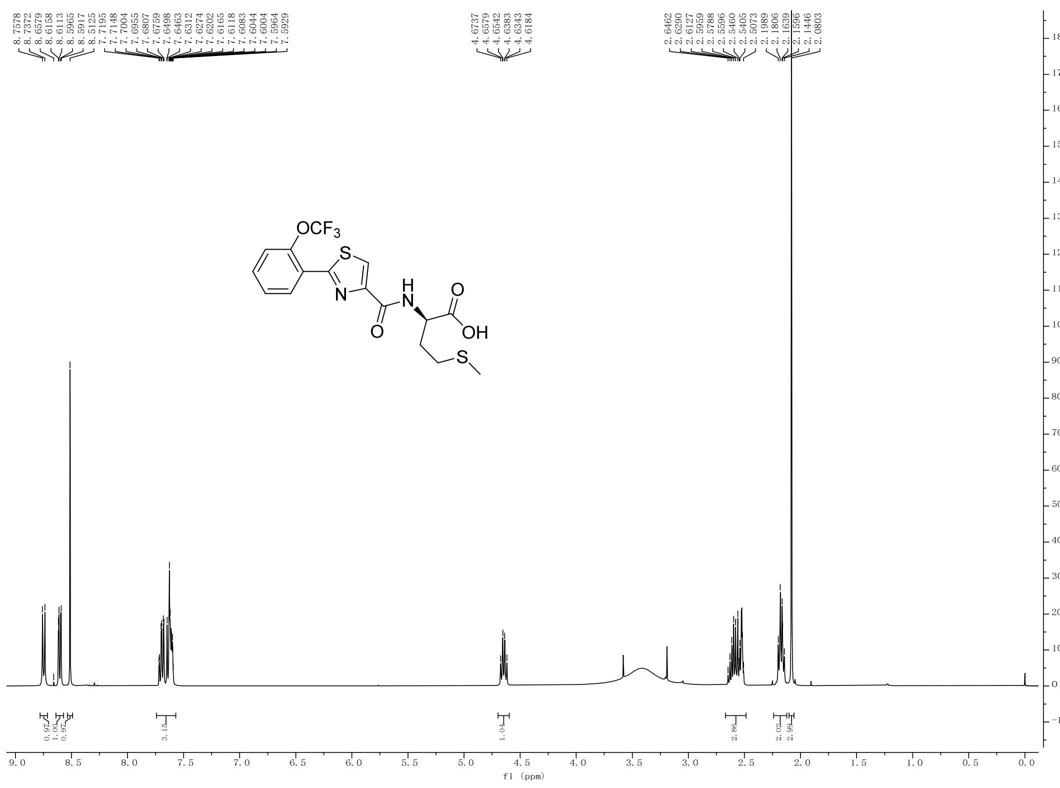

Figure S85. ^{1}H-NMR Spectrum of compound **9k**

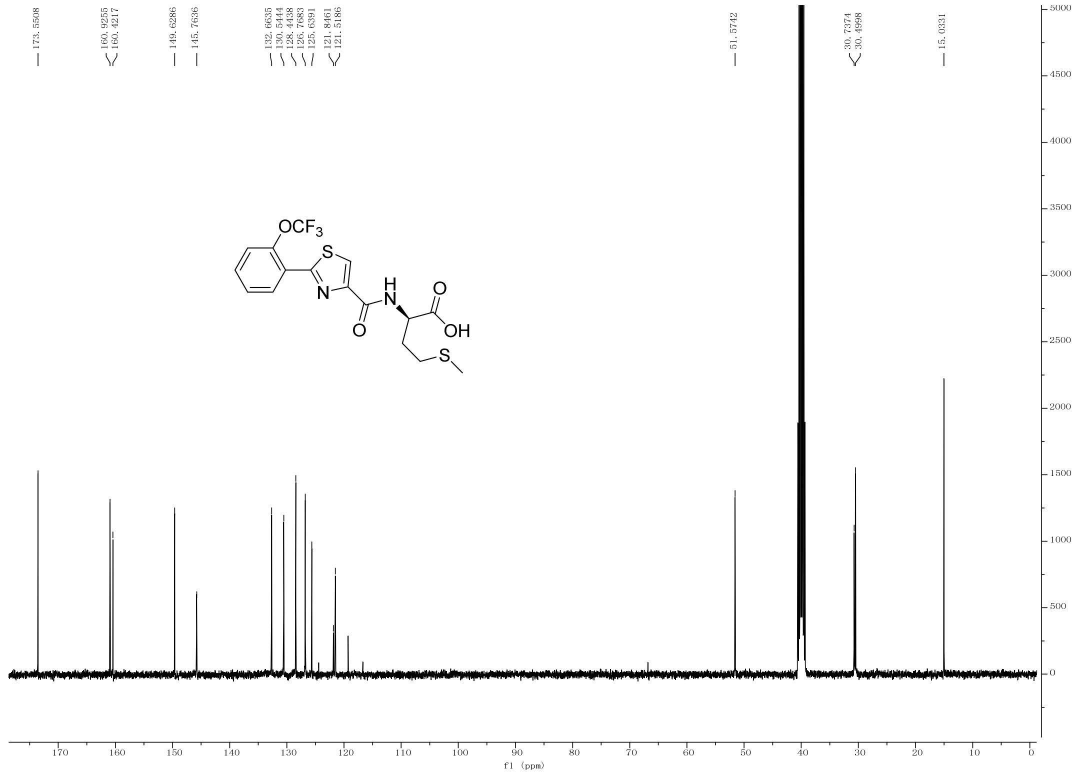

Figure S86. ^{13}C-NMR Spectrum of compound **9k**

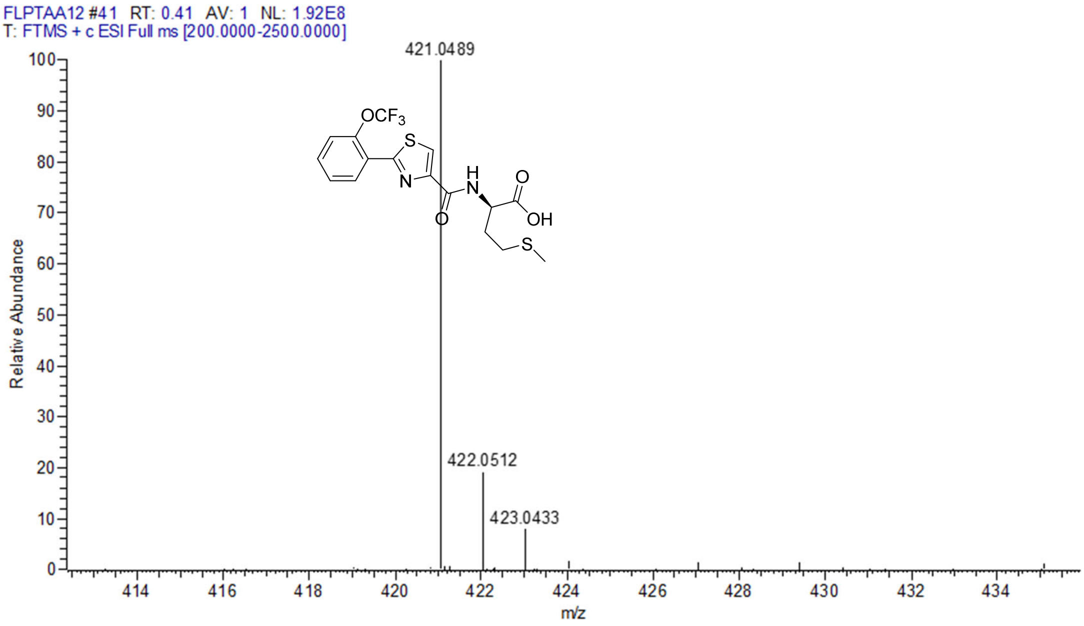

Figure S87. HRMS Spectrum of compound **9k**

9l: yellow solid; yield 74%, melting point171.2~173.2 °C; ^{1}H NMR (400 MHz, DMSO-d_6) δ 8.44 (s, 1H), 8.32 (d, J = 7.6 Hz, 1H), 8.22 (d, J = 8.0 Hz, 1H), 7.69 (t, J = 7.6 Hz, 1H), 7.60 (d, J = 8.0 Hz, 2H), 4.11 (dd, J = 4.4, 8.0 Hz, 1H), 2.28 – 2.16 (m, 1H), 0.90 (d, J = 7.2 Hz, 6H); ^{13}C NMR (101 MHz, DMSO-d_6) δ 160.54, 159.83, 150.62, 145.68, 132.65, 130.15, 128.64, 126.02, 125.67, 121.82, 121.77, 119.25, 59.29, 40.60, 40.39, 40.19, 39.98, 39.77, 39.56, 39.35, 31.66, 20.18, 18.55. HRMS(ESI) calcd for $C_{16}H_{16}F_3N_2O_4S^+$ [M+H]$^+$: 389.0777, found 389.0772.

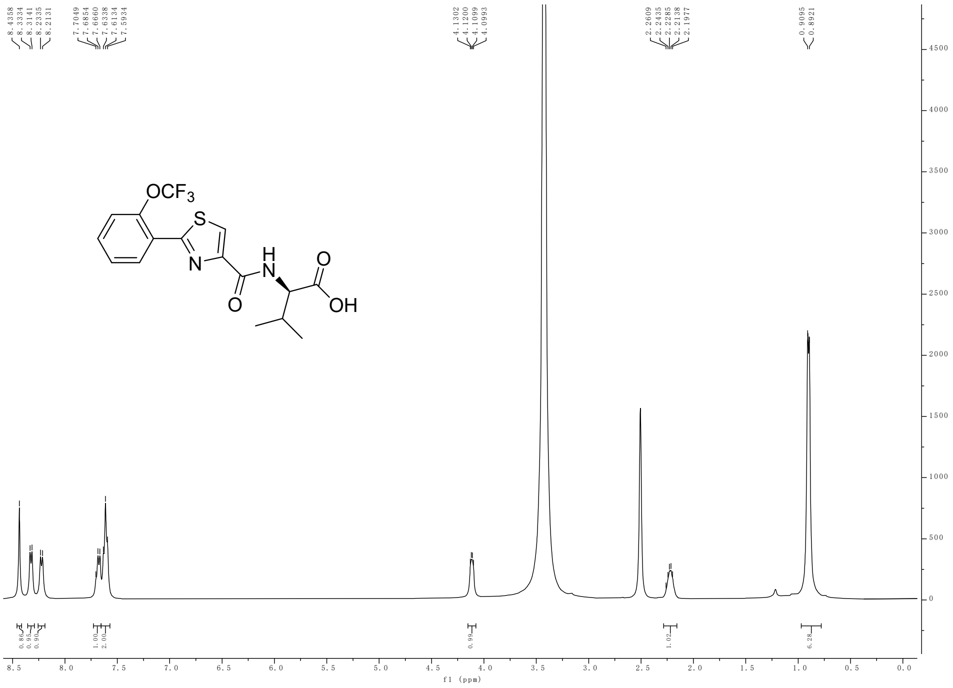

Figure S88. ^{1}H-NMR Spectrum of compound **9l**

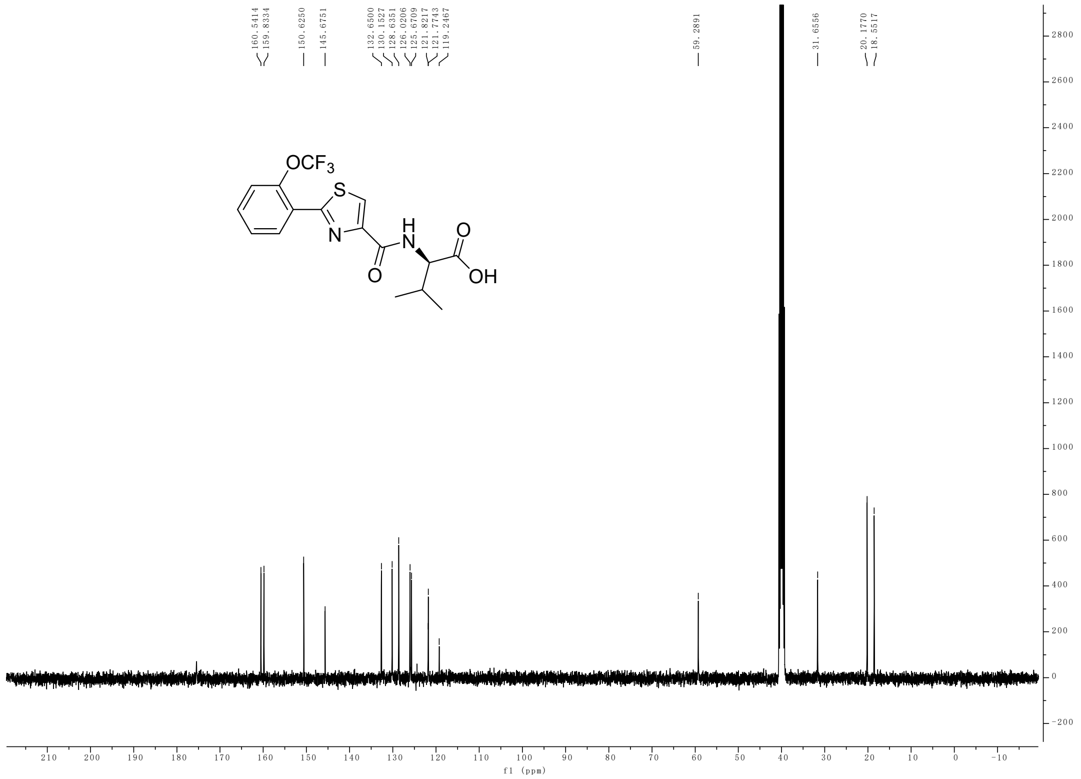

Figure S89. ^{13}C-NMR Spectrum of compound **9l**

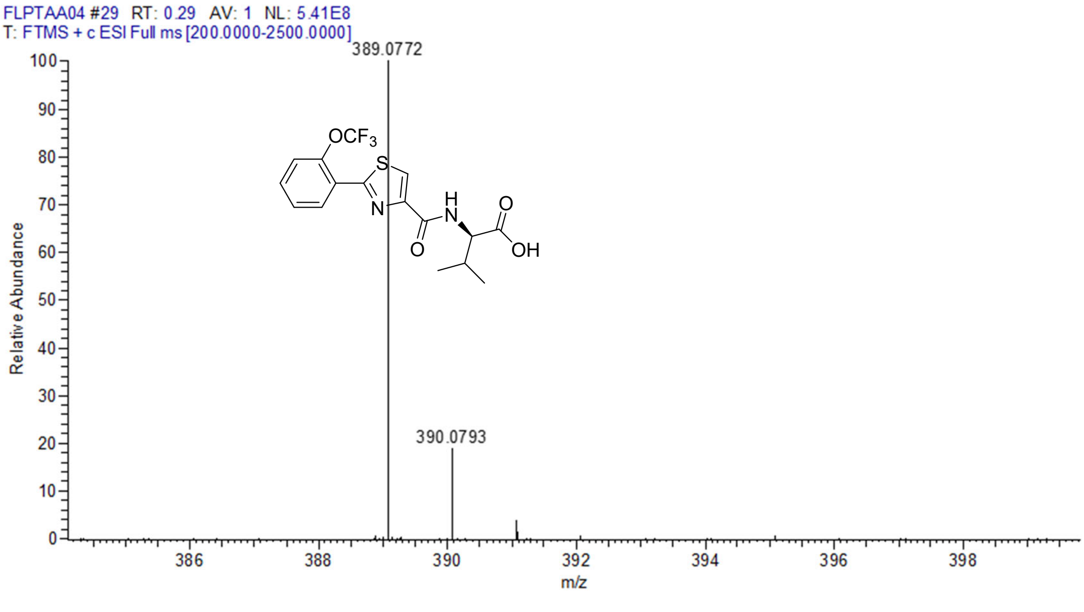

Figure S90. HRMS Spectrum of compound **9l**

9m: white solid; yield 78%, melting point 141.0~144.2 °C; ^{1}H NMR (400 MHz, DMSO-d_6) δ 12.83 (s, 1H), 8.59 (dd, J = 1.6, 8.0 Hz, 1H), 8.49 (s, 1H), 7.74 – 7.66 (m, 1H), 7.66 – 7.54 (m, 2H), 4.60 – 4.45 (m, 1H), 1.92 – 1.77 (m, 1H), 1.76 – 1.60 (m, 2H), 0.92 (dd, J = 6.0, 8.0 Hz, 6H); ^{13}C NMR (101 MHz, DMSO-d_6) δ 174.29, 160.67,

160.46, 149.73, 145.75, 132.67, 130.60, 128.49, 126.67, 125.66, 121.86, 121.56, 119.28, 50.90, 24.98, 23.43, 21.78. HRMS(ESI) calcd for $C_{17}H_{18}F_3N_2O_4S^+$ $[M+H]^+$: 403.0934, found 403.0925.

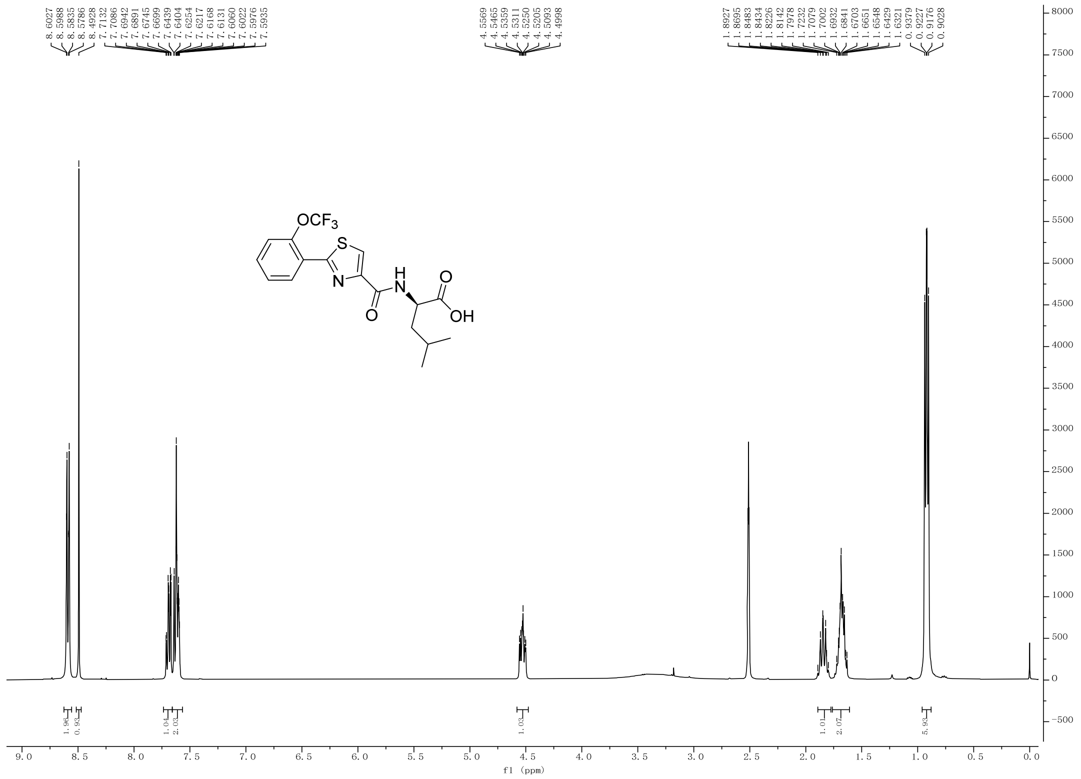

Figure S91. ^{1}H-NMR Spectrum of compound **9m**

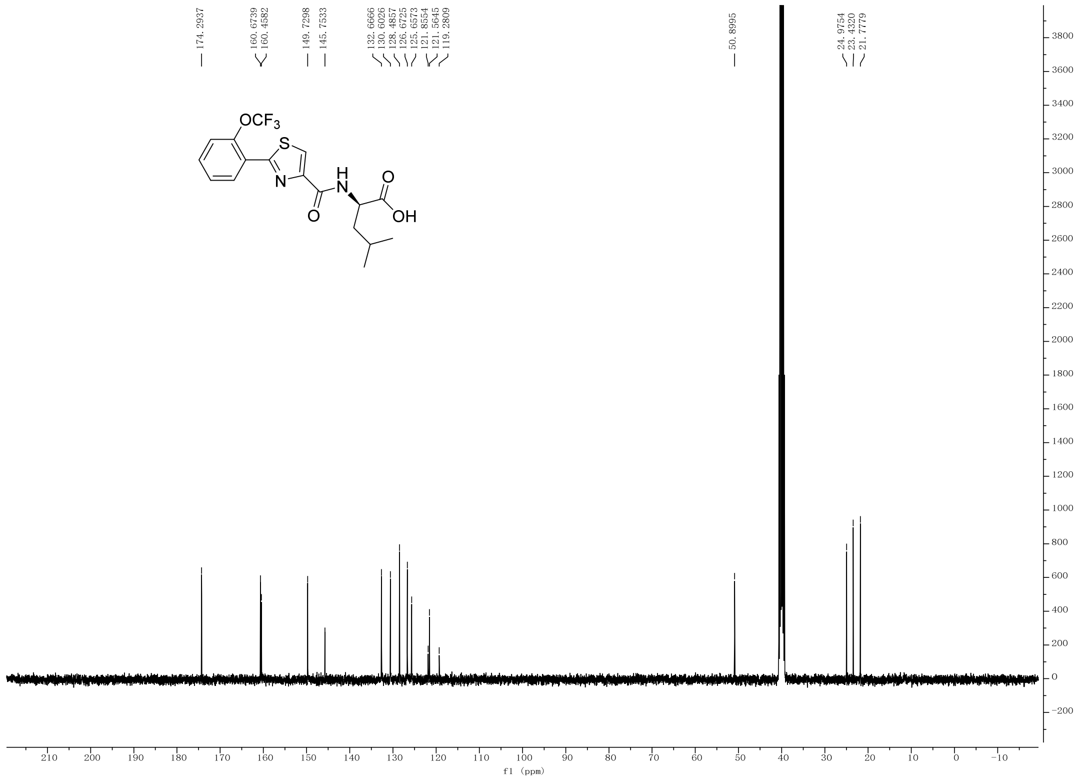

Figure S92. ^{13}C-NMR Spectrum of compound **9m**

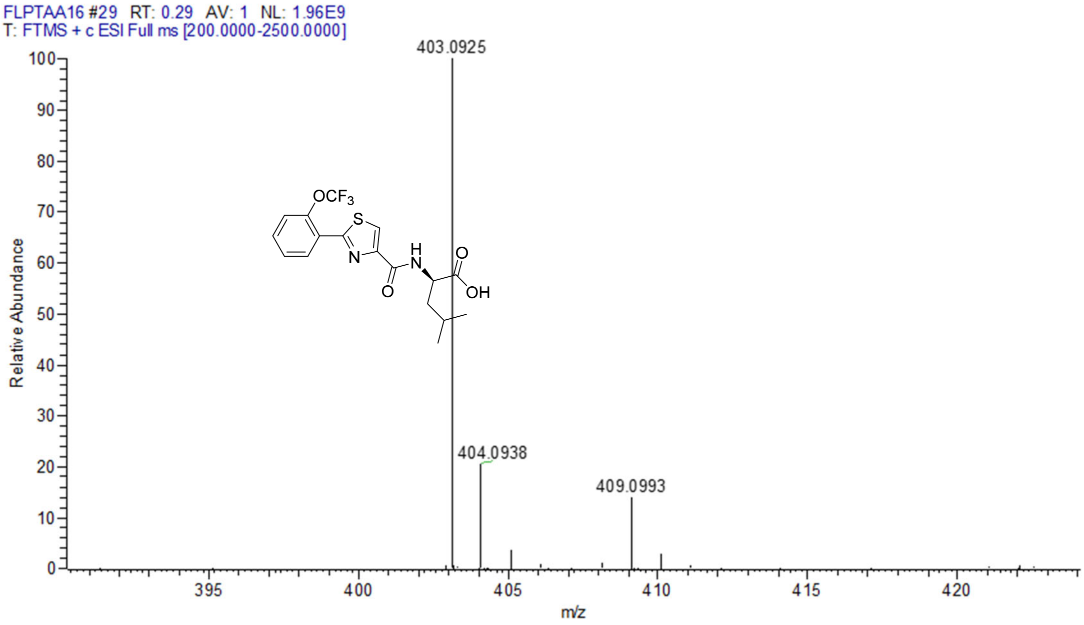

Figure S93. HRMS Spectrum of compound **9m**

9n: white solid; yield 74%, melting point 159.6~162.5 °C; ^{1}H NMR (400 MHz, DMSO-d_6) δ 13.00 (s, 1H), 8.61 – 8.47 (m, 2H), 8.44 (s, 1H), 7.75 – 7.55 (m, 3H), 7.33 – 7.22 (m, 4H), 7.23 – 7.15 (m, 1H), 4.78 – 4.67 (m, 1H), 3.26 – 3.10 (m, 2H); ^{13}C NMR (101 MHz, DMSO-d_6) δ 173.13, 160.49, 149.51, 145.76, 138.13, 132.73, 130.37, 129.60, 128.73, 128.52, 126.97, 126.70, 125.59, 121.84, 121.59, 119.26, 53.81, 36.77. HRMS(ESI) calcd for $C_{20}H_{16}F_3N_2O_4S^+$ [M+H]$^+$: 437.0777, found 437.0770.

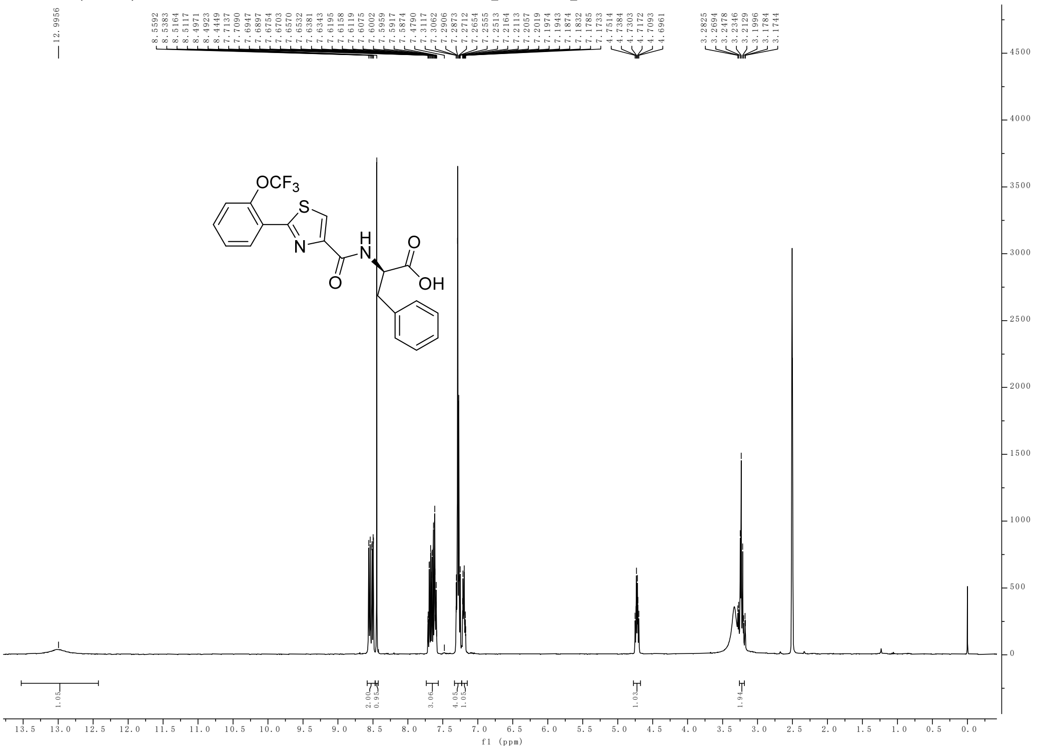

Figure S94. ^{1}H-NMR Spectrum of compound **9n**

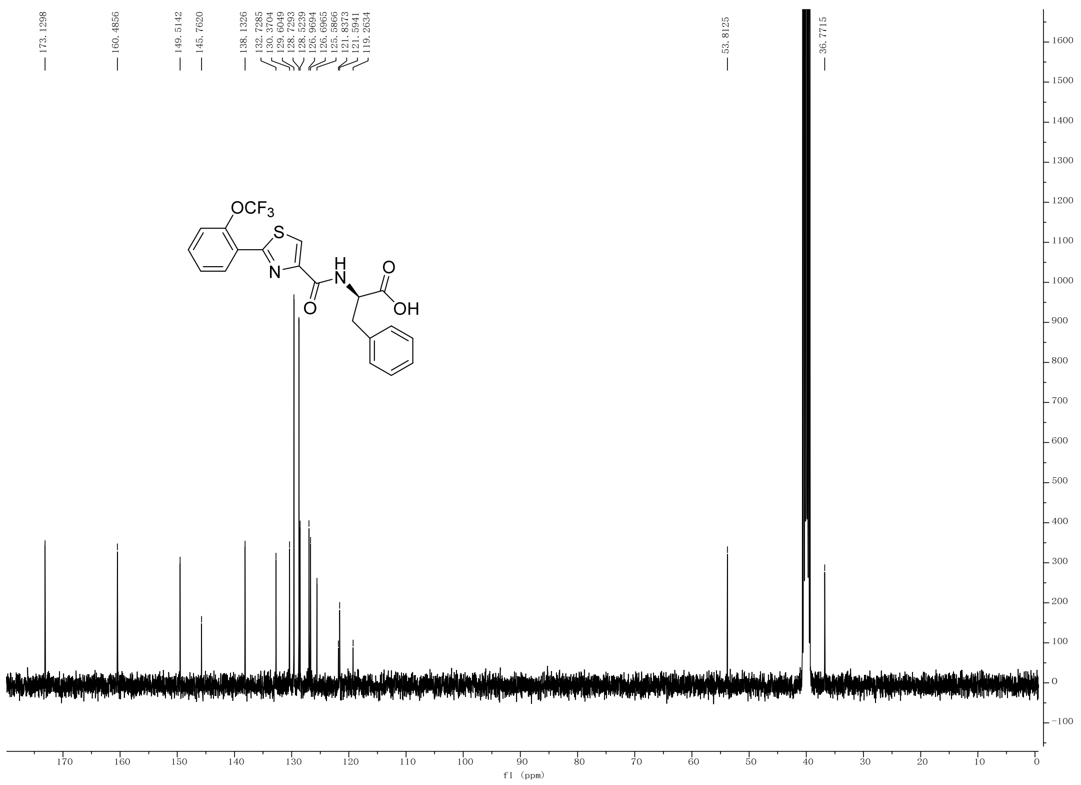

Figure S95. ^{13}C-NMR Spectrum of compound **9n**

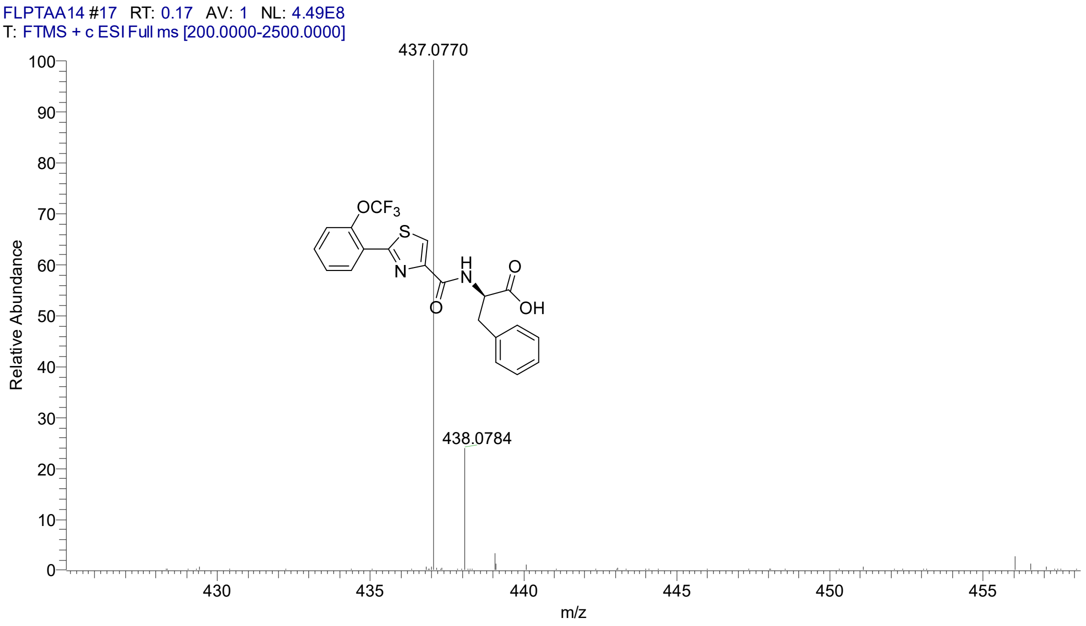

Figure S96. HRMS Spectrum of compound **9n**

9o: white solid; yield 76%, melting point 159.5~160.7 °C; ^{1}H NMR (400 MHz, DMSO-d_6) δ 8.43 (d, J = 9.2 Hz, 2H), 8.35 (d, J = 8.0 Hz, 1H), 7.70 – 7.63 (m, 1H), 7.63 – 7.54 (m, 2H), 7.11 – 7.02 (m, 2H), 6.69 – 6.60 (m, 2H), 4.64 – 4.53 (m, 1H),

3.18 – 3.03 (m, 2H); ^{13}C NMR (101 MHz, DMSO-d_6) δ 173.65, 160.40, 160.14, 156.41, 149.83, 145.74, 132.64, 130.66, 130.22, 128.50, 128.20, 126.36, 125.54, 124.40, 121.83, 121.53, 119.25, 115.45, 54.56, 36.36. HRMS(ESI) calcd for $C_{20}H_{16}F_3N_2O_5S^+$ $[M+H]^+$: 453.0727, found 453.0729.

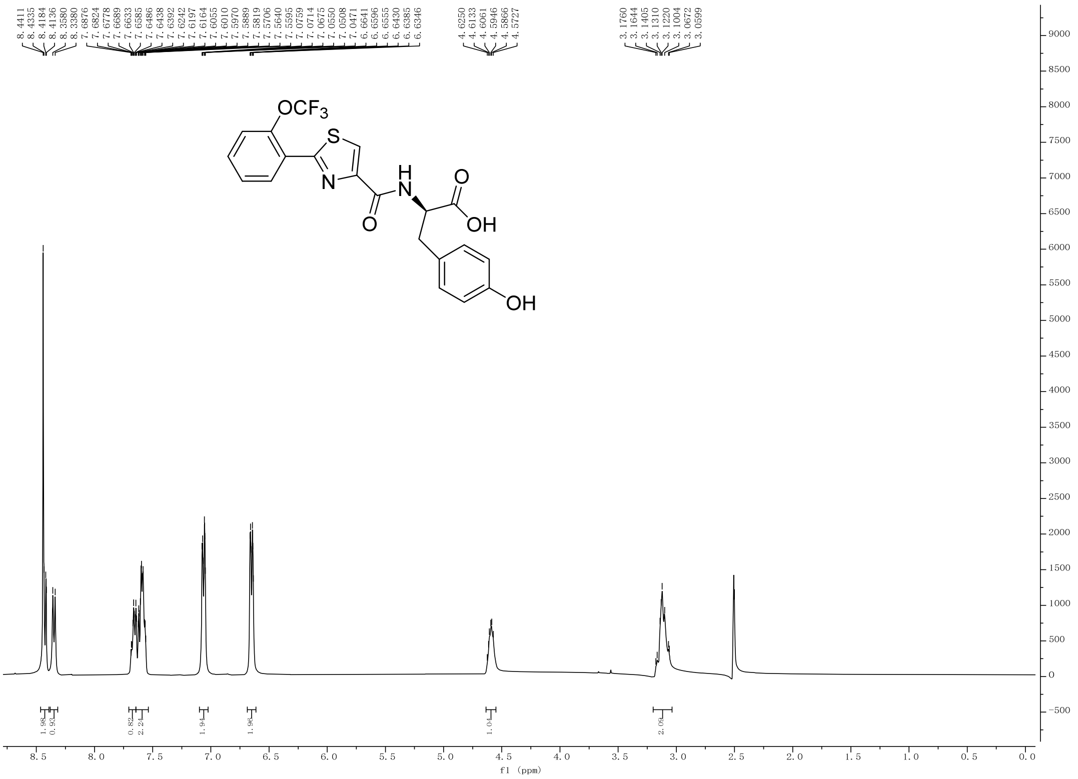

Figure S97. ^{1}H-NMR Spectrum of compound **9o**

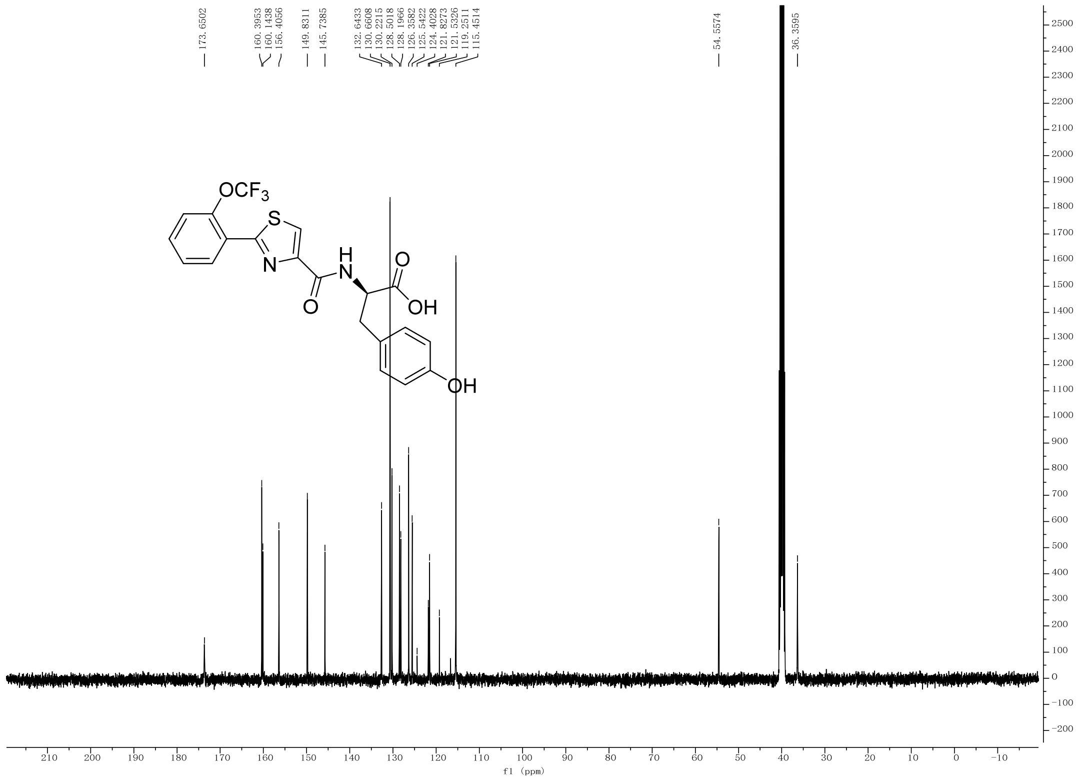

Figure S98. ^{13}C-NMR Spectrum of compound **9o**

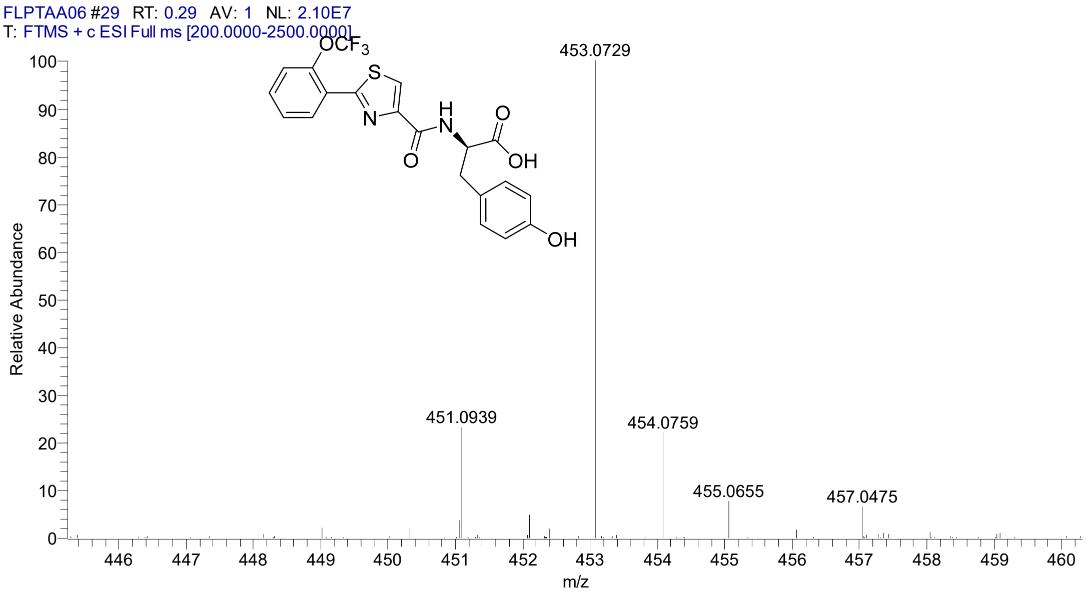

Figure S99. HRMS Spectrum of compound **9o**

9p: white solid; yield 75%, melting point 179.9~181.4 °C; ^{1}H NMR (400 MHz, DMSO-d_6) δ 10.91 – 10.81 (m, 1H), 8.44 (s, 1H), 8.30 (d, J = 8.0 Hz, 1H), 8.24 (d, J = 7.6 Hz, 1H), 7.71 – 7.62 (m, 1H), 7.61 – 7.49 (m, 3H), 7.32 (d, J = 8.0 Hz, 1H), 7.20 (d, J = 2.4 Hz, 1H), 7.03 (t, J = 7.6 Hz, 1H), 6.90 (t, J = 7.6 Hz, 1H), 4.66 (q, J = 6.4 Hz, 1H), 3.46 – 3.28 (m, 3H); ^{13}C NMR (101 MHz, DMSO-d_6) δ 160.27, 160.07, 150.07,

145.71, 136.48, 132.61, 130.19, 128.49, 128.23, 126.21, 125.50, 124.10, 121.81, 121.53, 121.19, 119.24, 118.91, 118.68, 111.72, 110.72, 54.45, 27.51. HRMS(ESI) calcd for $C_{22}H_{17}F_3N_3O_4S^+$ $[M+H]^+$: 476.0886, found 476.0877.

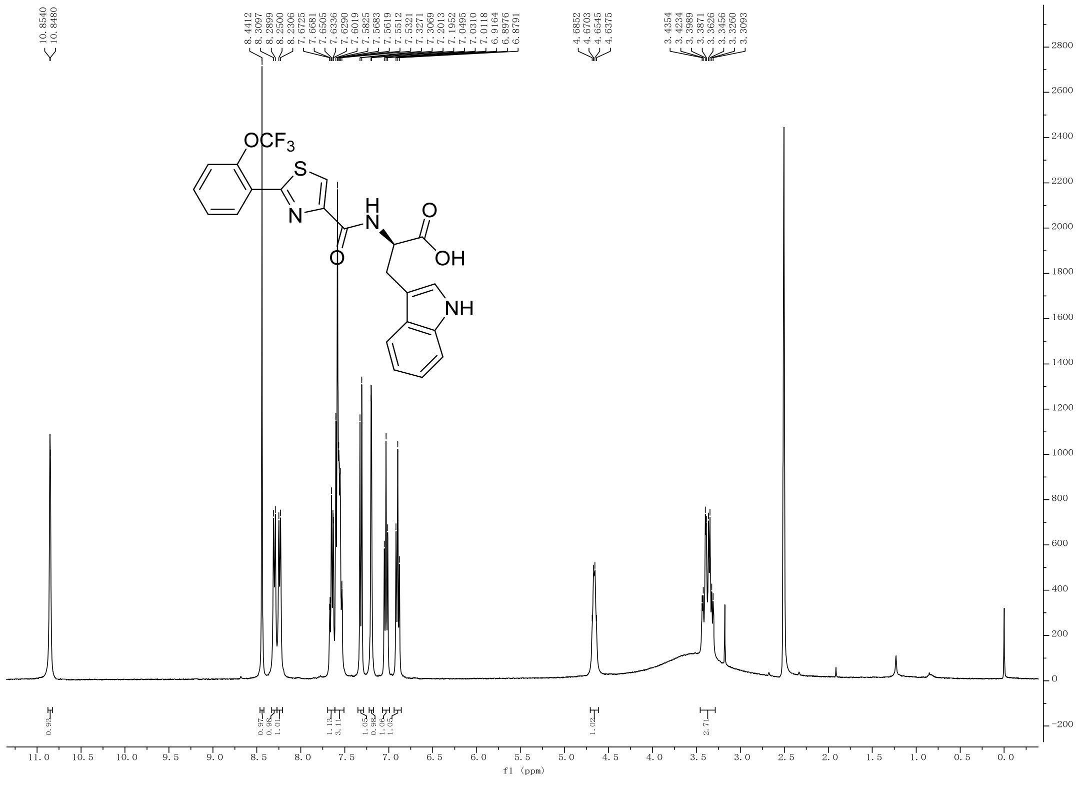

Figure S100. ^{1}H-NMR Spectrum of compound **9p**

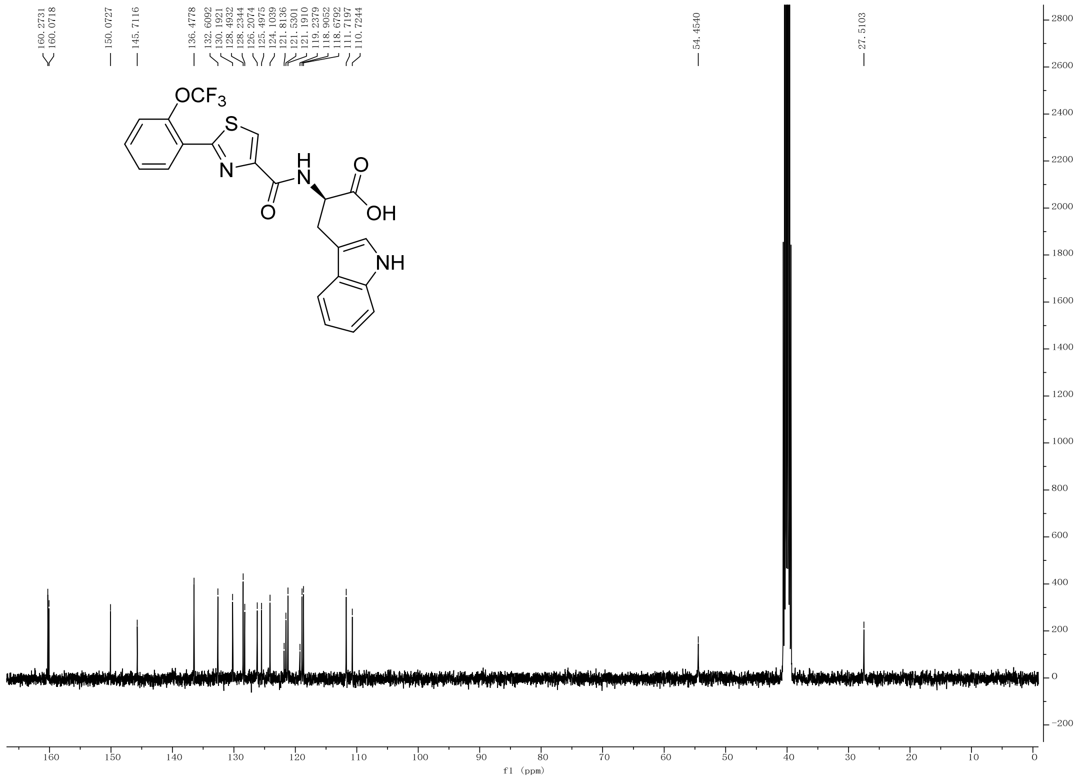

Figure S101. ^{13}C-NMR Spectrum of compound **9p**

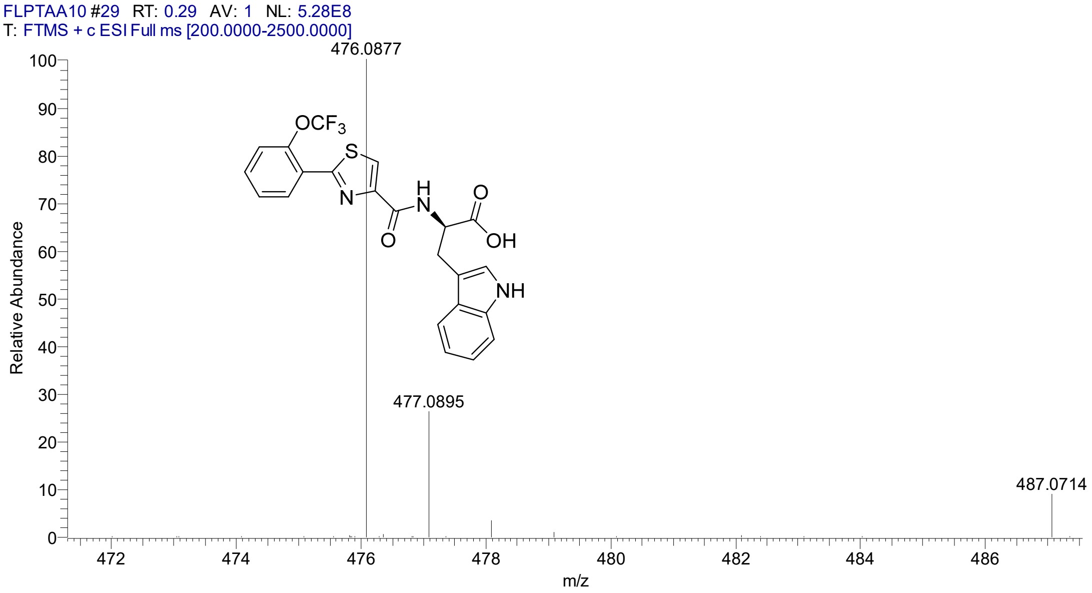

Figure S102. HRMS Spectrum of compound **9p**
